# Dissecting the autism-associated 16p11.2 locus identifies multiple drivers in neuroanatomical phenotypes and unveils a male-specific role for the major vault protein

**DOI:** 10.1186/s13059-023-03092-8

**Published:** 2023-11-15

**Authors:** Perrine F. Kretz, Christel Wagner, Anna Mikhaleva, Charlotte Montillot, Sylvain Hugel, Ilaria Morella, Meghna Kannan, Marie-Christine Fischer, Maxence Milhau, Ipek Yalcin, Riccardo Brambilla, Mohammed Selloum, Yann Herault, Alexandre Reymond, Stephan C. Collins, Binnaz Yalcin

**Affiliations:** 1 Institute of Genetics and Molecular and Cellular Biology, UMR7104, University of Strasbourg, CNRS, INSERM, IGBMC, U964, 67400 Illkirch, France; 2 Center for Integrative Genomics, University of Lausanne, CH-1015 Lausanne, Switzerland; 3 Inserm UMR1231, Université de Bourgogne, 21000 Dijon, France; 4 Institute of Cellular and Integrative neuroscience, CNRS, UPR321267000 Strasbourg, France; 5 School of Biosciences, Neuroscience and Mental Health Innovation Institute, Cardiff University, Cardiff, CF24 4HQ UK; 6 Dipartimento di Biologia e Biotecnologie “Lazzaro Spallanzani”, Università degli Studi di Pavia, Pavia, Italy; 7 University of Strasbourg, CNRS, INSERM, CELPHEDIA, PHENOMIN, ICS, 67400 Illkirch, France; 8 Current address: Université de Bourgogne, Inserm UMR1231, 21000 Dijon, France

**Keywords:** Mouse genetic studies, Autism spectrum disorders, Brain anatomy, Major vault protein, Sex differences

## Abstract

**Background:**

Using mouse genetic studies and systematic assessments of brain neuroanatomical phenotypes, we set out to identify which of the 30 genes causes brain defects at the autism-associated 16p11.2 locus.

**Results:**

We show that multiple genes mapping to this region interact to regulate brain anatomy, with female mice exhibiting far fewer brain neuroanatomical phenotypes. In male mice, among the 13 genes associated with neuroanatomical defects (*Mvp*, *Ppp4c*, *Zg16*, *Taok2*, *Slx1b*, *Maz*, *Fam57b*, *Bola2*, *Tbx6*, *Qprt*, *Spn*, *Hirip3*, and *Doc2a*), *Mvp* is the top driver implicated in phenotypes pertaining to brain, cortex, hippocampus, ventricles, and corpus callosum sizes. The major vault protein (MVP), the main component of the vault organelle, is a conserved protein found in eukaryotic cells, yet its function is not understood. Here, we find MVP expression highly specific to the limbic system and show that *Mvp* regulates neuronal morphology, postnatally and specifically in males. We also recapitulate a previously reported genetic interaction and show that *Mvp*^*+/−*^*;Mapk3*^*+/−*^ mice exhibit behavioral deficits, notably decreased anxiety-like traits detected in the elevated plus maze and open field paradigms.

**Conclusions:**

Our study highlights multiple gene drivers in neuroanatomical phenotypes, interacting with each other through complex relationships. It also provides the first evidence for the involvement of the major vault protein in the regulation of brain size and neuroanatomy, specifically in male mice.

**Supplementary Information:**

The online version contains supplementary material available at 10.1186/s13059-023-03092-8.

## Background

Autism spectrum disorders (ASDs) are a group of complex neurodevelopmental diseases characterized by restricted/repetitive behaviors and a deficit in social communication. Affected children usually express autistic behaviors after 24 months of age. ASDs are well known to be sex-biased with two to four males diagnosed for every female (reviewed in [1–4]). There are two main theories for the male predominance in ASDs. The extreme male brain theory, first proposed in 1997 [5], relates to the idea that ASDs could be extreme manifestations of the male brain and male-typical traits [6] that may be leading to a male ascertainment bias [7]. The female protective theory highlights the female’s brain being able to carry higher levels of genetic damage than the male’s brain without getting ASDs [8–11]. Apart from a study on the impact of hormones on human neural stem cells [12], the underlying sex-specific biology remains largely unknown.

The human 16p11.2 locus is susceptible to a 600-kb deletion (OMIM #611913) which is among the most frequent known etiologies of ASDs [13] with a male-female ratio of about two [14]. This 16p11.2 deletion usually arises de novo and partly associates with abnormal brain size and other structural defects including a decreased cortical thickness restricted to male carriers [15, 16]. Several deletion mouse models of the entire 16p11.2 locus also displayed reduced cortical areas [17–19]. One of the main challenges in the field is to decipher which of the 30 protein-coding genes mapping to this region underlies NeuroAnatomical Phenotypes (NAPs) and how they interact with each other leading to ASDs.

To evaluate the contribution of each gene within the 16p11.2 locus, systematic morpholino-mediated knockdown methods in the zebrafish model identified *KCTD13* (potassium channel tetramerization domain containing 13) as a main driver of brain size [20]. However, studies in the mouse model did not confirm this association [21, 22]. Intriguingly, *MVP*, which encodes the 100-kDa major vault protein in the interval, was found to modify NAPs via *cis* genetic epistasis with *KCTD13* [20, 21] and had synergistic effects in combination with *Kctd13 and Mapk3* for craniofacial measurements in mice [23].

Described in 1986 by Leonard H. Rome, the vault is the biggest ribonucleoprotein organelle present in many eukaryotic cells [24], three times the size of a ribosome. MVP is the main constituent of the vault organelle and is sufficient to give the vault its hollow barrel-like shape [25]. Tanaka et al. have resolved the structure of the rat vault at a 3.5-Å resolution as a particle made of 78 identical MVP [26]. MVP is not visible as free monomers at any given time in cells but instead appears only as vault particles [27]. MVP/vault is expressed in the cytoplasm, neurites, and growth cone of rat neurons [28].

Despite its discovery more than 30 years ago, the function of the vault organelle remains largely elusive. In vivo, MVP/vault associated with cortical plasticity in a monocular deprivation mouse model [29]. In vitro, the vault organelle binds to microtubules [30], and MVP interacts with ERK through epidermal growth factor signaling [31, 32] and associates with Aurora-A to enhance synapse formation and activity by the regulation of ERK signaling [33]. Interestingly, MVP has been coimmunoprecipitated and colocalized with steroid hormone receptors in human cancer cells [34, 35] and is involved in cellular trafficking for the modulation of Src-dependent ERK signal transduction induced by sex steroid hormones [31].

Here, we set out to assess, in an unbiased and systematic manner, the neuroanatomical implication of each individual gene of the 16p11.2 interval in males and females using mouse heterozygous mutants to inform the sex-specific genetic architecture of this autism-associated locus.

## Results

### Mouse genetic studies unravel the implications of multiple main drivers underlying neuroanatomical phenotypes at the 16p11.2 locus in a sex-specific manner

To identify which gene(s) regulate(s) mammalian brain architecture at the de novo 16p11.2 deletion locus, we aim to assess the alterations in NeuroAnatomical Phenotypes (named NAPs by us [36]), independently in male and female heterozygous (het) knockout (KO) mice, in the autism-associated B6N Del(7Sult1a1-Spn)6Yah mouse model of the entire 16p11.2 deletion (hereafter named as *Del/+*) as well as individually for each of the 30 protein-coding genes within the interval.

**Fig. 1:**
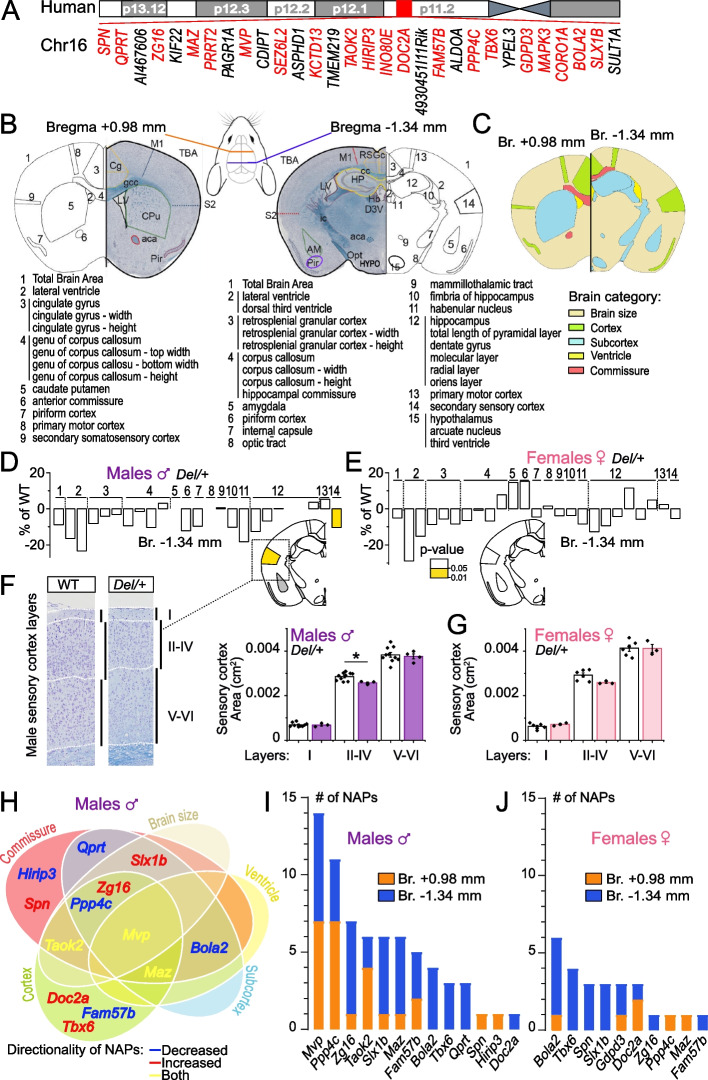
Mouse neuroanatomical studies for the identification of NAP genes at the 16p11.2 autism-associated locus. **A** Schematic representation of the human 16p11.2 region showing gene content and order with genes that underwent mouse neuroanatomical studies in red. **B** At bregma + 0.98 mm and bregma − 1.34 mm, 22 and 45 parameters were measured, respectively. **C** These 67 parameters (listed in Additional file 2: Table S2) are grouped into five categories (brain size, ventricle, cortex, commissure, and subcortex) on the two coronal sections. **D** Bar plots of the percentage change of male *Del/+* relative to male WT mice for each of the parameters measured. The colored region indicates the presence of a significant parameter at the 0.05 level. White indicates a *p*-value > 0.05. **E** Bar plots of percentage change of female *Del/+* relative to female WT. **F** Left: representative brain image stained with Nissl-luxol of adult male WT and *Del/+* mice showing the cortical layers of the somatosensory cortex at bregma − 1.34 mm. Right: histograms showing the thickness of the upper and lower layers of the somatosensory cortex in male *Del/+* mice. **G** Histograms showing the thickness of the upper and lower layers of the cortex in female *Del/+* mice. **H** Venn diagram illustrating NeuroAnatomical Phenotype (NAP) genes (genes whose mutations yield neuroanatomical phenotypes) in male mice positioned in each category. Phenotypic directionality is color-coded. Blue corresponds to a reduction in the size of the brain regions affected, red to increase, and yellow occurring of both. **I**, **J** Bar plots showing the number of NAPs per section (bregma + 0.98 mm in orange and bregma − 1.34 mm in blue) for each gene assessed for male (**I**) and female (**J**), respectively. Genes are listed on the *x*-axis and sorted according to the number of NAPs

We developed, or acquired through collaborations, 243 adult mutant and 221 colony-matched littermate wild-type (WT) mice, corresponding to the deletion of the entire locus as well as 20 unique gene deletions (highlighted in red in Fig. 1A), each studied with an average of four biological replicates, a number which we previously showed suitable to detect NAPs with effect size superior to 5% [36]. For the remaining 10 genes (in black in Fig. 1A), the germline transmission of the mutation failed despite multiple attempts or no mouse model was available during the course of the study. For one gene of interest (*Kctd13*), multiple allelic strategies were used. To ensure high comparability between the results, mouse mutants assessed in this study were all processed on identical genetic backgrounds (C57BL/6) and at the same age using the same standardized pipelines [37–40]. A detailed description of study samples and allelic constructions is provided in Additional file 2: Table S1 and Additional file 9.

Using a highly robust approach for the assessment of 67 coronal neuroanatomical parameters per brain sample (Fig. 1B and Additional file 1: Fig. S1), described in detail elsewhere [38], we systematically quantified the same two coronal brain sections at bregma + 0.98 mm and bregma − 1.34 mm and collected neuroanatomical measurements blind to the genotype (Additional file 3: Table S7). These parameters were grouped into five main categories: brain size, commissure, ventricle, cortex, and subcortex (Fig. 1C and Additional file 2: Table S2). After multiple quality control steps and critical evaluation of each phenotype, gene association was carried out within our internal database using a standardized statistical pipeline (Additional file 9). Heat maps of the assessed genes comprising percentage change relative to WTs and *p*-value are provided in Additional file 1: Fig. S2 for males, Additional file 1: Fig. S3 for females, and Additional file 4: Table S8.

Overall, the neuroanatomical profile associated with the male *Del/+* mice indicated a decreased size of the majority of regions assessed, but only the somatosensory cortex reached a significance level and was reduced in size by 10% (*p* < 0.05) (Fig. 1D). We asked whether alterations in specific cortical layers may contribute to this phenotype and found that it stemmed from the upper II–IV cortical layers that were thinner by 10% (*p* < 0.0091) (Fig. 1F). We did not detect any significant NeuroAnatomical Phenotypes in the female *Del/+* mice (Fig. 1E, G).

At the single-gene level, we identified 13 genes associated with NAPs (hereafter named NAP genes), when using a relaxed significance threshold of 0.05, associated with defects in commissure (ten genes), cortex (eight genes), subcortical structures (seven genes), brain size (five genes), and ventricle (three genes) in males (Fig. 1H). Eight genes (*Bola2*, *Qprt*, *Maz*, *Mvp*, *Ppp4c*, *Slx1b*, *Taok2*, and *Zg16*) gave significant results affecting two or more categories. *Mvp* was the only gene affecting all five main categories. The remaining five genes (*Doc2a*, *Fam57b*, *Hirip3*, *Spn*, and *Tbx6*) presented specific phenotypes in one brain category. In the 13 NAP genes, 38.5% decreased the size of the affected brain structures, while 38.5% increased their sizes, and 23% had bidirectional effects (genes in blue, red, and yellow, respectively, in Fig. 1H). When using a stringent significance threshold of 0.0001 to account for multiple comparisons, *Mvp* and *Tbx6* remained significant NAP genes.

Figure 1I shows the number of NAPs for each of the 21 individually deleted alleles in males. While *Mvp* still stood out as the strongest candidate gene based on the number of affected parameters (*n* = 14; all decreased in size except the lateral ventricles that were enlarged in size when compared to littermate WT mice), *Ppp4c* showed ten NAPs all decreased in size, *Zg16* implicated seven NAPs all increased in size, and *Taok2* showed six NAPs with three increased and three decreased in size. A heat map is provided in Additional file 1: Fig. S2, and neuroanatomical phenotypes are described in detail for each gene in Additional file 10.

Next, sex differences were assessed to determine the impact of each mutation on the female brain. Overall, female mice displayed a reduced number of NAP genes (10 as opposed to 13 in males), and for each of the nine genes in common between males and females, there were less affected brain parameters in females (Fig. 1J). Directionality of the phenotypes was consistent between sexes, but NAPs were significantly different with an excess of parameters at bregma − 1.34 mm for females (*p* = 0.00006, Fisher test) (Fig. 1I, J). *Gdpd3* was a female-only NAP gene, *Bola2* showed more severe anomalies in females, and *Mvp* showed no anomalies in females (Additional file 1: Fig. S4A). Female NAPs are described for each gene in detail in Additional file 1: Fig. S3 and Additional file 10.

Four genes (*Coro1a*, *Ino80e*, *Mapk3*, and *Kctd13*) were categorized as non-NAP, both in males and females. Considering the discrepancies in the literature [20–22], heterozygous *Kctd13*^*+/−*^ mice were assessed twice using two independent allelic constructions, which gave identical results with no NAPs (Additional file 9). We also studied the homozygous (hom) *Kctd13*^*−/−*^ mice and found a reduction in the size of the hippocampus by 10% (*p* = 0.015) for males (Additional file 1: Fig. S2 and Additional file 1: Fig. S4C-D) and 6% (*p* = 0.014) for females (Additional file 1: Fig. S3). This reinforces the existing link between *Kctd13* and hippocampal biology [21, 22].

To summarize, our systematic neuroanatomical screen demonstrates the implication of multiple main genes that drive NAPs at the 16p11.2 locus with the major vault protein, *Mvp*, gene being one of the top drivers. Our work also highlights the profound sex differences with NAPs being more prominent in males.

### The major vault protein is expressed in the limbic system in both sexes

Focusing on *Mvp*, the one gene that gave the most widespread NAPs across multiple brain categories in males (no NAPs in females), we thought to establish its expression distribution independently in male and female WT mouse brains to determine whether *Mvp* sex-specific NAPs are correlated to sex-specific changes in MVP expression levels.

**Fig. 2:**
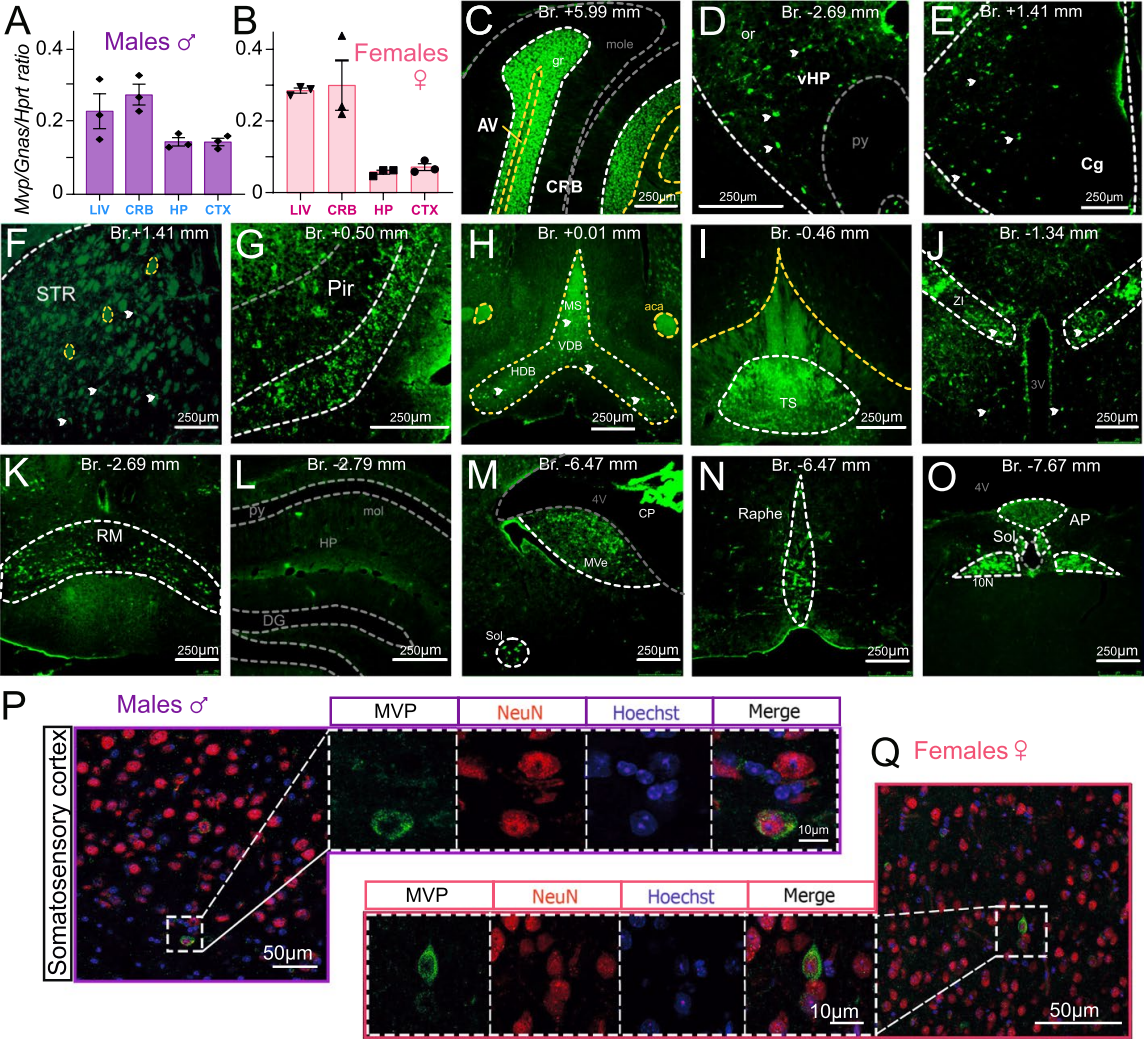
MVP expression is highly specific to the limbic system. **A**, **B** *Mvp* qRT-PCR expression in the liver (LIV), cerebellum (CRB), hippocampus (HP), and cortex (CTX), in male (**A**) and female (**B**) WT mice. Normalization was done using the *Gnas*/*Hprt* ratio. Plots are shown as mean ± SEM. Immunofluorescence of MVP (green) in CRB (**C**), ventral HP (**D**), and deep layers of the cingulate gyrus (Cg) (**E**). Arrowheads point to MVP-positive cells. AV, arbor vitae; gr/mole, granular/molecular layer of CRB; or/py, oriens/pyramidal layer of HP. **F**–**O** Selection of MVP immuno-histo-fluorescence (IHF) images on the coronal brain sections, each of 30 µm in thickness, performed throughout the entire brain from bregma + 1.41 mm to bregma − 7.67 mm. MVP presence was revealed using an anti-vault antibody (N2-B15) and secondary antibodies fused to Alexa488 fluorochrome (green). Dashed lines delineate regions with MVP-positive cells (white), MVP-positive tract (yellow), and MVP-negative cells (gray). It is noteworthy to mention that the experiment was repeated on a new set of brain samples on sagittal orientation, ranging from lateral + 0.24 mm to lateral + 0.84mm. Quantitative analysis both on the coronal and sagittal sections was done, and MVP expression was scored as mild (+), moderate (++), or strong (+++) across all the positive brain regions. The complete analysis is available in Additional file 2: Tables S3-S4. Pir, piriform cortex; MS, medial septum; DBB, diagonal band of Broca; TS, triangular septal nucleus; ZI, zona incerta; RM, retromammillary nucleus; py/mol, pyramidal/molecular layer of the hippocampus (HP); DG, dentate gyrus of the hippocampus; 4V, fourth ventricle; CP, choroid plexus; MVe, medial vestibular nucleus; Sol, solitary nucleus; AP, area postrema; 10N, vagus nucleus; Raphe, raphe magnus nucleus. **P**, **Q** IHF of MVP, NeuN neurons, and Hoechst on the brain sections from male (**P**) and female (**Q**) WT mice. The dashed square delineates the region presented at a higher magnification on individual channels for MVP, NeuN, and Hoechst, and merge of the three. In all structures, the MVP signal came from the cytoplasm

MVP transcripts, assessed at several developmental stages from embryonic day 16.5 (E16.5) to 30 weeks of age, were constant between males and females with a postnatal peak in line with an existing resource of expression profiles across multiple organs and developmental stages, which additionally revealed no expression of *Mvp* expression before E16.5 [41]. Expression was higher in peripheral tissues and the cerebellum than in the cortex and the hippocampus, both in males and females (Fig. 2A, B and Additional file 1: Fig. S5A-C).

The neuroanatomical spatial distribution of the major vault protein was then quantified by immunofluorescence using an anti-vault antibody, throughout four entire adult brains on consecutive histological sections by systematically counting the number of MVP/vault-positive cells and scoring as mild (+), moderate (++), or strong (+++). A total of 36 brain regions showed a positive signal (Additional file 2: Table S3) with equal scores between the sexes summarized in Additional file 2: Table S4. More specifically, MVP/vault signal was refined to the granular layer and the arbor vitae of the cerebellum (Fig. 2C), the oriens layer of the CA3 region of the ventral hippocampus (Fig. 2D), and the deep layers of the cingulate gyrus (Fig. 2E). Interestingly, we noticed MVP/vault signal pertained to specific nuclei of the limbic system (Fig. 2F–O), for example, the triangular septal nucleus (Fig. 2I), the zona incerta (Fig. 2J), the solitary nucleus (Fig. 2M), the vagal nucleus (Fig. 2O), the paraventricular hypothalamic nuclei, and the dorsal medial thalamic nucleus (Additional file 2: Table S4). At the subcellular level, MVP/vault signal was limited to the cytoplasm of neurons both in males and females (Fig. 2P, Q and Additional file 1: Fig. S5D-F). It was noteworthy that the patterns observed were consistent across sectioning planes (coronal and sagittal) and replicates.

To sum up, our data show no apparent sex differences in the pattern of MVP/vault expression that could explain the sex differences in NAPs. We cannot however exclude possible sex differences in the levels of MVP expression in other cell types of the brain that will require further investigations.

### The major vault protein is implicated in the regulation of brain size and neuronal morphology in males only

**Fig. 3:**
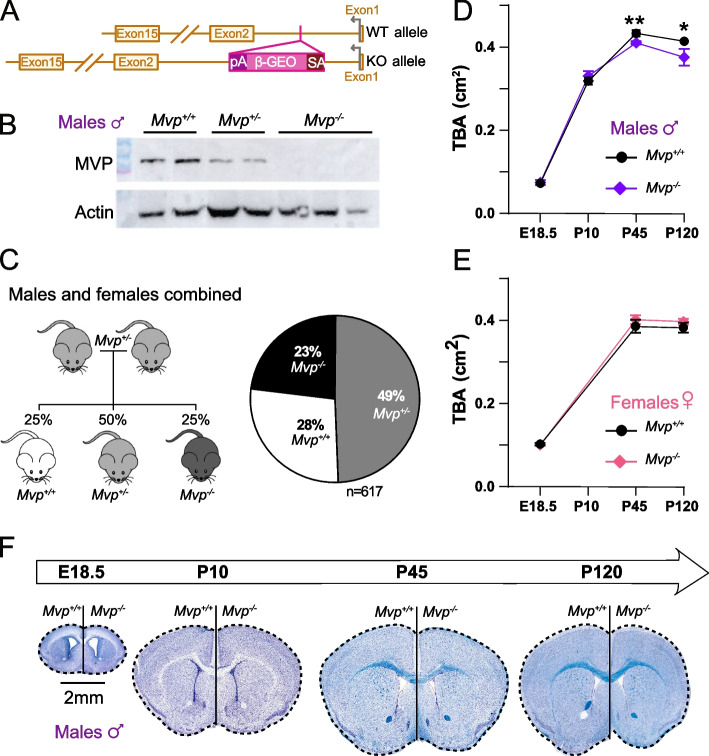
Validation and phenotyping of *Mvp*-deficient mice. **A** Schematic representation of *Mvp* knockout model construction. The knockout mutants were obtained by a promoter gene trap design, resulting in the insertion of a β-galactosidase/neomycin phosphotransferase (β-GEO) cassette within the first intron of the *Mvp* gene. The polyA-tail (pA) serves to end the transcription and thus discard the transcription of *Mvp* gene. **B** Validation of *Mvp* knockout at the protein level using Western blot (WB) from liver extracts of *Mvp*^*+/+*^ (*n* = 2), *Mvp*^*+/−*^ (*n* = 2), and *Mvp*^*−/−*^ (*n* = 3) mice. Actin was used as a loading control for normalization. For quantification, see Additional file 1: Fig. S6D. **C** Viability screen of *Mvp* knockout mice. Left: schematic representation of heterozygous-by-heterozygous breeding scheme, with expected Mendelian ratio of segregation of the offspring. Right: graph represents the ratio of each genotype of successfully genotyped mice obtained from heterozygous-by-heterozygous breeding (*n* = 617). **D**, **E** Graph showing the total brain area (1_TBA) measured at four time points, embryonic age 18.5 (E18.5), postnatal day 10 (P10), P45, and P120, in male (**D**) and female (**E**) *Mvp*^*−/−*^ mice (*n* = 5–7 in each group). **F** Half images of the coronal brain sections stained with Nissl-luxol at the four time points from male *Mvp*^*+/+*^ and *Mvp*^*−/−*^ mice. Plots are shown as mean ± SEM. Two-tailed Student *t* test equal variance (**D**, **E**). *p < 0.05, **p < 0.01

The MVP mouse model used in this study was validated as loss-of-function (LoF) of *Mvp*, assessed at the transcript and protein levels (Fig. 3A, B, Additional file 1: Fig. S6A-D and Additional file 9). We also verified that the expression of neighboring genes was unaffected (Additional file 1: Fig. S6E). Among 617 successfully genotyped animals, we observed normal Mendelian ratio inheritance, indicating that the loss of *Mvp* has no effect on survival (Fig. 3C and Additional file 1: Fig. S6F).

We studied the dynamics of brain size across four time points: embryonic day 18.5 (E18.5), postnatal day 10 (P10), P45, and P120, using our systematic and standardized procedures [37–40]. At E18.5 and P10, the total brain area measurement in male *Mvp*^*−/−*^ mice was normal but smaller at P45 (− 7%, *p* = 0.0061) and P120 (− 9%, *p* = 0.046), suggesting that the microcephaly is acquired between P10 and P45 (Fig. 3D, F). Interestingly, a recent study found that the protein level of MVP became higher at P35 [33], which could explain why the microcephaly phenotype became visible after this point at P45 in our study. Female *Mvp*^*−/−*^ brain size was normal across the time points assessed (Fig. 3E). In addition to the total brain area, we quantified 40 other parameters at E18.5 (Additional file 2: Table S5) and did not find any phenotypes at this stage in both sexes.

**Fig. 4:**
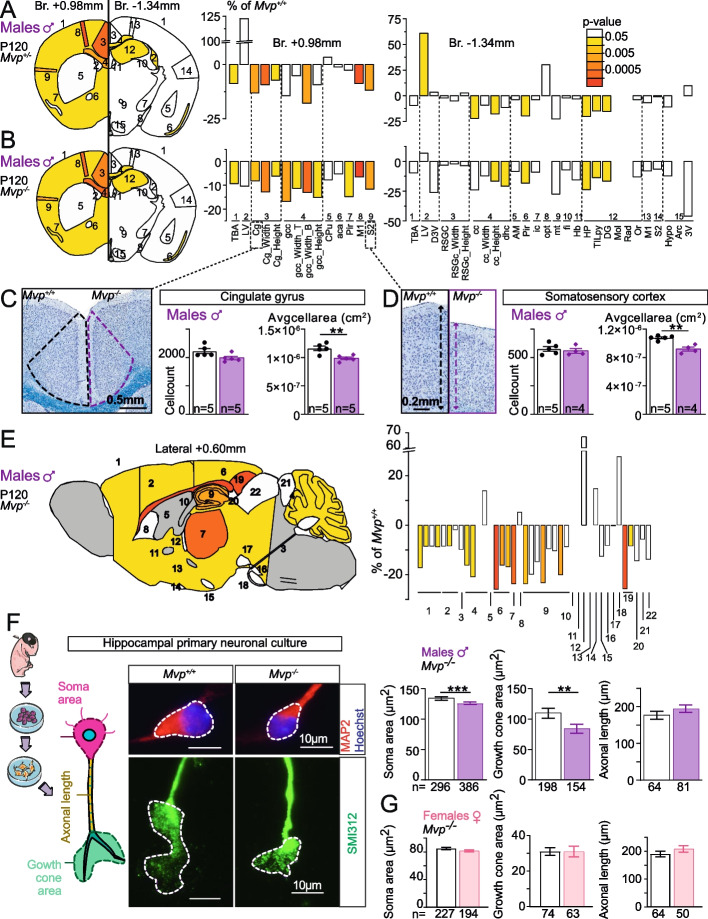
Involvement of the major vault protein in male-specific neuroanatomical phenotypes. **A**, **B** Left: schematic representation of the 24 brain regions assessed at bregma + 0.98 mm and − 1.34 mm in male *Mvp*^*+/−*^ (**A**) and male *Mvp*^*−/−*^ (**B**) mice at P120. Colored regions indicate the presence of at least one significant parameter within the brain region at the 0.05 level. White indicates a *p*-value > 0.05, and gray shows not enough data to calculate a *p*-value. Right: histograms of percentage change relative to *Mvp*^*+/+*^ for each of the 42 parameters (see Additional file 2: Table S2). **C**, **D** Left: coronal sections of Cg (**C**) and somatosensory cortex S2 (**D**) from *Mvp*^*+/+*^ and *Mvp*^*−/−*^ at P120. Right: cell count and average cell area measures within Cg and S2. **E** Left: schematic representation of the 22 brain regions quantified at lateral + 0.60 mm on the parasagittal section from *Mvp*^*+/+*^ and *Mvp*^*−/−*^. Colored regions indicate the presence of at least one significant parameter within the brain region at the 0.05 level. White indicates a *p*-value > 0.05, and gray shows not enough data to calculate a *p*-value. Right: histograms of percentage change relative to *Mvp*^*+/+*^ (set as 0) for each of the measured parameters (listed in Additional file 2: Table S6). **F** Left: schematic representation of a hippocampal neuron in culture and the different measures taken. Middle: neurons stained with MAP2 (red) and SMI312 (green). Right: graphs showing measures of soma area, growth cone area, and axonal length from hippocampal neurons derived from multiple male *Mvp*^*+/+*^ and *Mvp*^*−/−*^ embryos. **G** Graphs showing measures of soma area, growth cone area, and axonal length from hippocampal neurons derived from multiple female *Mvp*^*+/+*^ and *Mvp*^*−/−*^ embryos (*n* > 3 embryos per group, Additional file 5: Table S9). Plots are shown as mean ± SEM (except for **A**, **B**, and **E**). Two-tailed Student *t* test equal variance (**C**, **D**, **F**, and **G**). *p < 0.05, **p < 0.01, ***p < 0.001

At P120, het male *Mvp*^*+/−*^ mice showed the same reduction in total brain size (− 9%, *p* = 0.016) when compared to hom male *Mvp*^*−/−*^ mice (− 9%, *p* = 0.046) (Fig. 4A, B). Consistently, both *Mvp*^*+/−*^ and *Mvp*^*−/−*^ male mice showed small brain nuclei associated to the limbic system such as the cingulate gyrus (*Mvp*^*+/−*^: − 13%, *p* = 0.0016; *Mvp*^*−/−*^: − 8%, *p* = 0.007), the somatosensory cortex (*Mvp*^*+/−*^: − 12%, *p* = 0.0025; *Mvp*^*−/−*^: − 12%, *p* = 0.0016), and the hippocampus (*Mvp*^*+/−*^: − 20%, *p* = 0.023; *Mvp*^*−/−*^: − 24%, *p* = 0.038). In female *Mvp*^*−/−*^ mice, no change was detected at any given time (Additional file 1: Fig. S7A). The number and effect size of NAPs being similar between male *Mvp*^*−/−*^ and *Mvp*^*+/−*^ mice (Fig. 4A, B: 17 NAPs versus 14, respectively), we performed subsequent studies specifically comparing *Mvp*^*−/−*^ to Mvp^*+/+*^. NAPs in male *Mvp*^*−/−*^ mice were also confirmed on sagittal planes using a previously described procedure [37] with 15 parameters significantly smaller in size at P120 including the cingulate gyrus (− 25%, *p* = 0.000065), the hippocampus (− 24%, *p* = 0.0069), the corpus callosum (− 26%, *p* = 0.000013), and the thalamus (− 24%, *p* = 0.00033) (Fig. 4E).

To investigate whether neuronal morphology may contribute to brain size phenotype in male *Mvp*^*−/−*^ mice, we took advantage of the high resolution of our approach and developed a suite of automated tools to count the number of cells and calculate the average cell size within each brain region (Additional file 9). No significant change was detected in the number of cells in male *Mvp*^*−/−*^ mice; however, cells were significantly smaller in size in all affected brain regions including the cingulate gyrus (− 15%, *p* = 0.0053) (Fig. 4C) and somatosensory cortex (− 15%, *p* = 0.001) (Fig. 4D). Cell size was normal in unaffected brain region such as the retrosplenial cortex (Additional file 1: Fig. S8F). The same set of studies were conducted in female *Mvp*^*−/−*^ mice, but no defects in cell count and size were found (Additional file 1: Fig. S7B-D). To further characterize the cellular phenotype, we conducted hippocampal neuronal cultures, independently in males and females (Fig. 4F, Additional file 9 and Additional file 5: Table S9). Consistently, neurons derived from male *Mvp*^*−/−*^ mice showed a reduction of the soma size by 6% (*p* = 0.0003). The growth cones were smaller by 23% (*p* = 0.0081), and no differences were detected for the axonal length (Fig. 4F). Neurons derived from female *Mvp*^*−/−*^ showed no cell morphological defects (Fig. 4G).

Finally, to test if the anatomical changes relate to neural connectivity defects in male *Mvp*^*−/−*^ mice, we measured miniature excitatory postsynaptic currents (mEPSCs) in pyramidal neurons of the anterior cingulate gyrus (Additional file 9). The mEPSC amplitude was smaller (*p* = 0.023) (Additional file 1: Fig. S9A), and the density of postsynaptic dendritic spines was reduced (− 5%, *p* = 0.02) (Additional file 1: Fig. S9D), indicating functional and morphological changes in synaptic connections. Neuronal ultrastructure was examined, but no anomalies were seen in the cingulate gyrus (Additional file 1: Fig. S9E-F). To identify potential biological pathways that might explain NAPs, we carried out transcriptomic analyses in the cingulate gyrus, and although the complete loss of *Mvp* was confirmed, no differentially expressed genes were found (Additional file 1: Fig. S9G), suggesting that *Mvp* has no major role on bulk transcriptional regulation that could explain the phenotypes at the time point sampled.

Altogether, these findings show that *Mvp* is not essential for survival and is not implicated in neuronal proliferation processes but instead in the regulation of neuronal size, morphology, and function, as well as in the maintenance of brain homeostasis after birth, specifically in males.

### The double hemideletion of Mvp and Mapk3 alters behavioral performances in male mice

To determine if NAPs in male *Mvp*^*−/−*^ mice lead to specific behavioral phenotypes, we assessed a broad range of sixteen behavioral tests evaluating eleven core behaviors (anxiety, depression, anhedonia, memory, locomotion, coordination, motricity, sociability, schizophrenia, autism, and epilepsy). The raw data is available in Additional file 6: Tables S10, Additional file 7: Tables S11, and Additional file 8: Tables S12, and the behavioral pipeline is described in detail in Additional file 9.

**Fig. 5:**
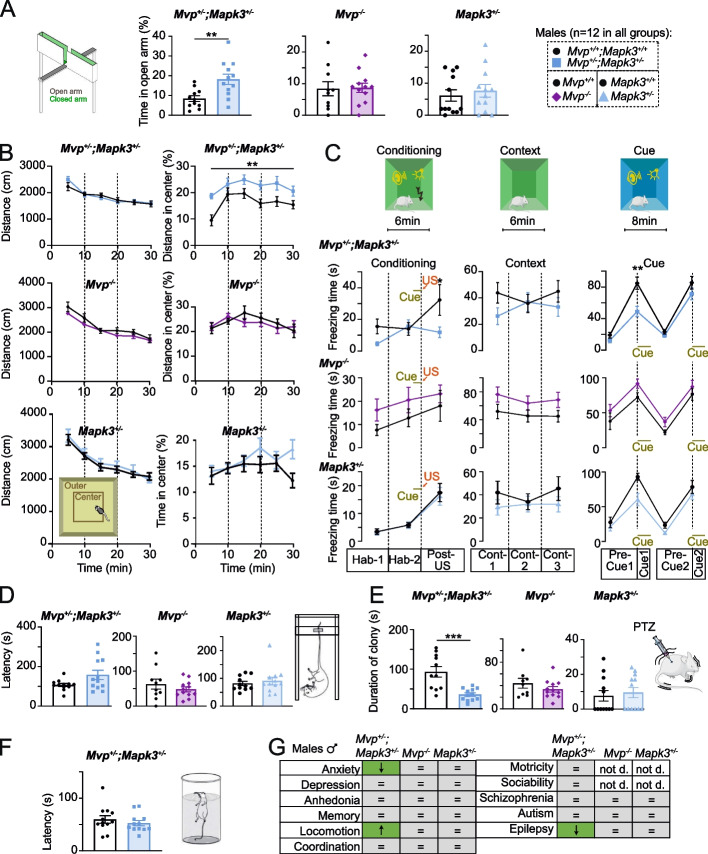
Behavioral analysis of *Mvp* and *Mapk3* genes. Selection of behavioral paradigms among 17 tested in 12 *Mvp*^*+/+*^*;Mapk3*^*+/+*^ and 12 *Mvp*^*+/−*^*;Mapk3*^*+/−*^ mice, from 11 to 25 weeks of age. Elevated plus maze (**A**). Open field (**B**). Fear conditioning (**C**). Tail suspension (**D**). PTZ test (**E**). Forced swim (**F**). **G** Summary table of core functions assessed in various cohorts. Green indicates significance, gray no difference, and white indicates not done (not d.). Arrows indicate the directionality of the effect, and the equal signs indicate no changes. A comprehensive description of behavioral tests, raw datasets, and additional findings are provided in Additional file 9, Additional file 6: Tables S10, Additional file 7: Tables S11, Additional file 8: Tables S12, and Additional file 1: Figs. S10-S12, respectively. The mean and standard error of the mean are shown in the graphs. Two-tailed Student *t* test equal variance (**A**, **D**–**F**) and two-way ANOVA with Sidak post hoc (**B**, **C**). *p < 0.05, **p < 0.01, ***p < 0.001

Intriguingly, we found no major behavioral anomalies in male *Mvp*^*−/−*^ mice (Fig. 5 and Additional file 1: Fig. S10). We thought to test whether introducing an additional stressor might underscore behavioral implications. To explore this idea, we generated and characterized the behavior of double male KOs of *Mvp* and *Mapk3* (Additional file 1: Fig. S12 and Additional file 9) since studies have consistently shown MVP-mediated regulation of ERK signaling pathway [31–33], which is known to be one of the main pathways disrupted at the 16p11.2 locus [23].

Double *Mvp*^*+/−*^;*Mapk3*^*+/−*^ male mice showed normal body weight (Additional file 1: Fig. S12A) as well as normal motricity, coordination, reward-seeking behaviors, working and short-term memory, associative learning, circadian activity, and no signs of autism and schizophrenic-like behaviors (Additional file 1: Fig. S12B-K). However, double *Mvp*^*+/−*^;*Mapk3*^*+/−*^ male mice spent more time in the open arm of the elevated plus maze (Fig. 5A). While the open-field test indicated no increase in traveled distance suggesting normal basic activity, it showed increased time spent in the center of the apparatus (Fig. 5B) and decreased latency to enter the center (Additional file 1: Fig. S12L), suggesting resistance to anxiogenic behavior. Fear conditioning in contextual and cued testing showed decreased freezing time (Fig. 5C). The tail suspension test showed a trend of increased latency to immobility (Fig. 5D), while the forced swim test did not show any apparent differences (Fig. 4F). The duration of clonic seizures was dramatically reduced in the pentylenetetrazol (PTZ)-induced seizure paradigm (Fig. 5E), supporting the idea of a resistance to epilepsy also. Additionally, we studied the same series of eleven core functions and assessed the phenotypes of male *Mapk3*^*+/−*^ mice. Like male *Mvp*^*−/−*^ mice, we found no behavioral anomalies in male *Mapk3*^*+/−*^ mice (Fig. 5 and Additional file 1: Fig. S11), suggesting that *Mvp* or *Mapk3* alone is not sufficient to potentiate the behaviors seen in the double *Mvp*^*+/−*^;*Mapk3*^*+/−*^ male mice. A summary of behavioral findings is provided in Fig. 5G. Taken together, these results confirm the in vivo interaction between MVP and ERK1. Finally, preliminary findings, where we examined ERK activity in the cortex of our various mouse models in males and females, indicate that ERK activity is not associated with NAPs or behavioral phenotypes presented in this study (Additional file 1: Fig. S12M-P and Additional file 10).

## Discussion

Our findings are important in at least three respects. First, our main aim was to identify which of the 30 single-gene(s) at the 16p11.2 locus cause(s) NeuroAnatomical Phenotypes (NAPs) reported in various deletion mouse models of the locus [17–19].

We found multiple single-gene drivers of a plethora of NAPs with opposite effects within the 16p11.2 interval that likely undergo a combination of additive and synergistic interactions as reported in this region [42–44] and other loci [45] (reviewed in [46]). Our neuroanatomical study of the *Del/+* mice confirms complex relationships between the genes as we only found one significant phenotype pertaining to the somatosensory cortex, suggesting that interactions between genes at the locus can hide NAPs driven by individual genes. Among the 20 syntenic single-genes assessed in this study (of note, all 20 met the expression detection threshold in mouse brain tissues [47]), two were associated with decreased (− 9% for *Mvp* and − 10% *Ppp4c*) and three with increased (+ 7% for *Slx1b*, + 6% for *Taok2*, and + 7% for *Zg16*) size of the brain. This alone could explain the variable MRI findings in patients harboring the 16p11.2 deletion as individuals do not share the same genetic background and each has a unique set of complex gene effects [48, 49]. We did not find any brain morphological changes in *Kctd13*^*+/−*^ mice, consistent with two recent mouse studies [21, 22]. With the exception of *Taok2*, linked to enlarged brain size in a recent report [50] and this study, NAP genes identified were new from the literature, both in mice and humans, potentiating neurodevelopmental disease gene discovery. Among non-NAP genes, findings were consistent with a report of *Prrt2*^*−/−*^ mice that did not find any overt abnormalities in brain size [51].

Second, we provide the first evidence that MVP modulates brain and neuronal size in key structures of the limbic system including the cingulate and somatosensory cortices. Interestingly, MVP has a highly specific pattern of expression in the limbic system which is well-known to regulate behavioral responses to emotional stimuli [52]. We found no differential expression changes in the cingulate gyrus, arguing against a bulk transcriptional effect of the loss of *Mvp*. Because MVP appears only as vault particles at any given time in cells [27], MVP findings directly implicate the vault organelle. The vault has previously been associated with synaptic transmission in various cell models [29, 33, 53]. The vault might also be implicated in the transport of mRNAs along neurites for local translation at the synapse [28], through Aurora-A and small noncoding vault RNA that has been shown to modulate synapse formation by amplifying ERK signaling [33], although the totality of mRNA species that bind to the vault organelle remains to be identified. In this study, we found a decrease in postsynaptic spine density and mEPSC amplitude in pyramidal cingulate neurons, suggesting that the loss of *Mvp* also disrupts neural connections, which in turn could explain alterations in neuronal size and homeostasis.

Third, we provide insights into the determinants of sex differences at the 16p11.2 locus associated with decreased cortical thickness restricted to male carriers [15, 16] in a suitable model, sexing being not feasible in fruit fly [44] and zebrafish embryos [20, 43]. From an anatomical perspective, our study shows that the female mouse brain is more resistant when a genetic mutation is involved while the male brain is more vulnerable. This brings experimental evidence on the “female protective effect” previously predicted in human neurodevelopmental disorders [54]. Our findings on MVP neuronal co-expression patterns exclude differences at the anatomical level between males and females as a potential cause responsible for the underlying sex differences in NAPs, favoring the hormonal hypothesis [55].

ERK1/2 kinase signaling cascades are central in the control of neuronal plasticity and size [56] and can be activated in response to sex hormones [57]. While hyperphosphorylation of ERK1 has been previously reported in 16p11.2 deletion mouse models [58, 59], sex differences in ERK phosphorylation were overlooked [18, 60], with the exception of one recent study [59]. On this ground, pharmacological ERK inhibitors have been used to improve neurological traits [58] and more generally in neurodevelopmental and neurodegenerative disorders [61]. In the current study, hemiablation of *Mapk3* showed increased ERK1 activation in the male cortex not female, reinforcing a hormone-dependent regulation of kinase cascades. MVP was found to interact with steroid hormone receptors [34, 35] and is involved in sex-dependent ERK signal transduction induced by hormones [31]. We hypothesize that, in the absence of MVP/vault, there are compensatory biological mechanisms in the female brain and not in the male brain.

Finally, human and mouse MVP share approximately 90% of their amino acid residues, and the vault organelle likely plays a similar role in the human brain. Human *MVP* alone has not been implicated as yet in shaping brain size, possibly due to a polygenic model involving other genes of the 16p11.2 locus such as *MAPK3* or to a lack of functional evidence for its possible role in neurobiology. Interestingly, a smaller deletion region for autism encompassing five out of the 30 genes has been narrowed down [62] and included *MVP*.

## Conclusions

To conclude, we have characterized 20 individually deleted genes in mice using ultra-standardized histological methods and found that there is more than one gene playing a role in brain morphogenesis at the 16p11.2 locus. The *Mvp* gene is one of the top drivers based on the number of affected neuroanatomical parameters and correlated with smaller neuronal size but not cell counts. Interestingly, female mice are less affected by gene inactivation at the locus, which may be a potential explanation why neurodevelopmental disorders such as autism at the 16p11.2 locus predominantly affect boys, with female *Mvp*^*+/−*^ and *Mvp*^*−/−*^ mice showing no associated phenotypes. We also confirm MVP implication in the ERK1 signaling pathway in vivo and define *MVP* as a novel candidate gene in male-specific neurodevelopmental disorders.

## Methods

Here, we provide a summary of our main methods. For a full description, see Additional file 9.

### Study samples

The mouse syntenic 16p11.2 region *Sult1a-Spn* encompasses 30 protein-coding genes on chromosome 7. Among these, 20 (Additional file 2: Table S1) were incorporated in our analysis of neuroanatomical phenotyping explained in Additional file 1: Fig. S1. The remaining ten were not available during the course of the study for reasons explained in Additional file 9. Genotyping primers were designed in our own animal facility (Mouse Clinical Institute, Illkirch, France). A list of sequences is provided in Additional file 9. Sexing was determined visually except for embryos and young postnatal age where we combined visual assessment with genetic testing using the SRY reactions. For validation purposes, a set of mutants was studied multiple times through the production of independent cohorts or at different ages.

### Mouse model of the 16p11.2 locus

Del(7Sult1a1-Spn)6Yah mice deleted for the entire 16p11.2 locus (named as *Del/+*) were generated at the Mouse Clinical Institute, Illkirch, France. The deletion encompassed the region from *Sult1a* to *Spn* and was previously described [63]. In this study, we decided to test the impact of the chromosomic rearrangement in the pure genetic background strain (C57BL6/N), to facilitate data integration with the single-gene knockout mice all processed on identical genetic backgrounds (C57BL/6).

### Generation of double knockouts

We generated double-knockout lines by crossing single-gene mutants producing WT and double-het, as well as intermediate heterozygotes for each single-gene mutant. Four groups of mice were used in ERK-activity assays consisting of *Mvp*^*+/+*^*;Mapk3*^*+/+*^, *Mvp*^*+/−*^*;Mapk3*^*+/+*^, *Mvp*^*+/*^*;:Mapk3*^*+/−*^, and *Mvp*^*+/−*^*;Mapk3*^*+/−*^.

### Brain sample processing

The brains were taken from adult mice on a high-throughput phenotyping project, where each mouse was characterized by a series of standardized operating procedures. The collection of brain samples was performed blind to the genotype. The brains from at least four mice per genotype and per gender were collected. The control brains were systematically collected within each of the mutant lines. Every aspect of the procedure was managed through a relational database using the FileMaker (FM) Pro database management system (detailed elsewhere [36]). A similar approach was used for embryos at E18.5 fixed in the Bouin solution.

### Neuroanatomical studies and quality control

An overview of sample processing is shown in Additional file 1: Fig. S1. The brains were cut at a thickness of 5 μm on a sliding microtome (HM450, Microm, France) on symmetrical and stereotaxic planes to obtain coronal sections matching bregma + 0.98 mm and bregma − 1.34 mm. Our precision was estimated to be no more than 30 μm, anterior or posterior, to the histological section of interest. The sections were double-stained with 0.1% Luxol Fast Blue (Solvent Blue 38, Sigma-Aldrich) and 0.1% Cresyl violet acetate (Sigma-Aldrich), in order to label myelin and neurons, respectively. After mounting on slides, the sections were scanned at cell-level resolution using the Nanozoomer whole-slide scanner 2.0HT C9600 series (Hamamatsu Photonics, Shizuoka, Japan).

Each image was quality controlled to assess whether (i) the section is at the correct position, (ii) the section is symmetrical, (iii) the staining is of good quality, and (iv) the image is of good quality. Only images that fulfilled all of the quality control checks were fully processed. These quality control steps are essential for the detection of small to moderate neuroanatomical phenotypes (NAPs) and without which the large majority of NAPs would be missed. This is explained in great detail elsewhere [36]. On the coronal plane, 67 brain morphological parameters of 39 area and 28 length measurements were taken for each sample (Additional file 2: Table S2) [38]. Forty-two neuroanatomical coronal measurements were taken at E18.5 (Additional file 2: Table S5) [39] and 40 on the sagittal plane (Additional file 2: Table S6) [37]. Cellular features across histological sections were measured using a semi-automated macro designed in Fiji measuring cell counts as well as averaged cell area for each cell.

### MVP expression

Several mouse cohorts underwent microdissection in order to separate various brain structures of interest at various developmental and adulthood stages. Total RNA was extracted using a phenol-chloroform technique. One microgram of total RNA was reverse-transcribed to complementary DNA using Superscript III First-Strand Synthesis Supermix (11752–050, Invitrogen) for qualitative and quantitative reverse transcription PCR (RT-PCR). For the latter, we used TaqMan assays.

### Immuno-fluorescence

C57BL/6N adult mice of 12 weeks of age (three males and one female) were anesthetized, intracardially perfused with PBS, and fixed with 4% paraformaldehyde (PFA) solution in PBS. The brains were harvested, post-fixed in 4% PFA for 24 h at room temperature (RT), and then transferred in 30% sucrose solution for 24 h at 4 °C. Each brain was trimmed either for coronal or sagittal sectioning and embedded in Cryomatrix (Thermo Scientific) with a fast freeze device PrestoChill (Millestone). The brains were sectioned throughout the entire tissue blocks with a cryo-microtome (K400 station with dry ice on HM450 microtome, Microm). Immunostainings for MVP were realized without detergent using floating sections. The permeabilization step was done by incubating the brain sections in methanol at − 20 °C for 10 min. The sections were rinsed in PBS, blocked in a solution with 10% normal donkey serum (NDS) and 1% bovine serum albumine (BSA) in PBS, and incubated with rabbit polyclonal vault antibodies (N2-B15, provided by Leonard Rome, dilution 1:1000) and mouse anti-NeuN antibodies (MAB377, Millipore, dilution 1:1000) in 0.1%NDS/PBS at 4 °C overnight under agitation. After washing with PBS, the sections were incubated for 3 h RT with fluorescence-conjugated secondary antibodies coupled to anti-rabbit-Alexa-488 and anti-mouse-Alexa-647 (1:1000, Thermo Fischer Scientific). Images were acquired using a confocal microscope (TCS SP5, Leica) at × 20 or × 80 magnification and analyzed using ImageJ.

Phospho-ERK immunohistochemistry was carried out on floating sections. After quenching with 3% H_2_O_2_, 10% methanol for 15 min, the sections were rinsed in TBS and incubated for 1 h in a blocking solution (5% normal goat serum, 0.1% Triton X-100). The sections were then incubated overnight at 4 °C with anti-phospho-p44/42 MAP kinase (Thr202/Tyr204) (1:1000, Cell Signalling Technology, Danvers, MA). On the following day, a biotinylated goat anti-rabbit secondary antibody (1:200, Vector Labs) was applied to the sections for 2 h at room temperature. Detection of the bound antibodies was carried out using a standard peroxidase-based method (ABC-kit, Vectastain, Vector Labs), followed by incubation with DAB and H_2_O_2_ solution. The sections were subsequently dehydrated using increasing concentrations of ethanol and mounted with DPX. Images were acquired from the prefrontal cortex using a bright field microscope (Leica DMI6000B Macro/Micro imaging system) under a × 40 magnification and analyzed with ImageJ.

### Western blot

The cortex and liver from adult mice were dissected and homogenized with 300 μL of lysis buffer containing 1× RIPA buffer (Thermo Fisher), phenylmethylsulfonyl fluoride 1%, sodium orthovanadate 1%, and protease inhibitor 1% in tubes containing ceramic beads (Precellys Lysing Kit). The tubes were incubated for 30 min at 4 °C and centrifuged for 20 min at 17,000×*g* at 4 °C, and the supernatant was isolated for Western blotting. Sixty micrograms of protein was separated on 10% SDS/PAGE (Mini-PROTEAN TGX Gels, 12%, 10-well, 4561043, BIO-RAD) and transferred onto nitrocellulose membrane (#1620115, BIO-RAD). The membranes were blocked with 1% BSA diluted in Tris-buffered saline with Tween 20 (50 mM Tris, 150 mM NaCl, 0.05% Tween 20) and probed using anti-MVP (named “George,” provided by Leonard H. Rome) overnight at 4 °C. Antibody–protein interactions were revealed using chemiluminesence (RPN2108, GE Healthcare), and relative protein expression was quantified using ImageJ.

### Primary neuronal culture

The hippocampus was harvested from embryos at day 18.5 (E18.5) for cell dissociation. Between 20,000 and 30,000 cells were plated on Poly-l lysine-coated coverslips in 24-well plates and incubated at 37 °C with 5% CO_2_. After 4 days of in vitro culture (DIV4), the cells were fixed with 4% PFA in 6% sucrose for 15 min and then stored in EtOH at 4 °C until use. Fixed cells were incubated O/N at 4 °C with primary antibodies (rabbit polyclonal anti-MAP2 (AB5622, Millipore) and mouse monoclonal antibody against pan-axonal neurofilament (SMI-312R, Covance) both diluted at 1:1000 in saturation solution (0.2% Triton, 1% BSA, 10% normal donkey serum (S2170, Dutscher) in Tris-buffered saline). Donkey anti-rabbit coupled to Alexa647 (ab150075, Abcam) and donkey anti-mouse coupled to Alexa488 (ab150105, Abcam) were used as secondary antibodies in saturation solution without Triton. The nuclei were stained with Hoechst 3342 (1:10,000 dilution, Sigma-Aldrich). Images were acquired using a regular epifluorescence microscope × 100 (Leica) at a magnification of × 0.55 and analyzed using ImageJ.

### Bulk RNA sequencing

RNA sequencing libraries were generated from 200 ng of total RNA from mouse cingulate gyrus using TruSeq Stranded mRNA LT Sample Preparation Kit (Illumina, San Diego, CA), according to the manufacturer’s instructions. The details are provided in Additional file 9. The libraries were sequenced on the Illumina Hiseq 4000 as single-end 50 base reads following Illumina’s instructions. Image analysis and base calling were performed using RTA 2.7.3 and bcl2fastq 2.17.1.14. Reads were preprocessed using cutadapt 1.10 [64] in order to remove adapter, polyA, and low-quality sequences (Phred quality score below 20); reads shorter than 40 bases were discarded for further analysis. Reads mapping to rRNA and spike-in sequences were also discarded (this mapping was performed using bowtie 2.2.8 [65]). Reads were then mapped onto the mm10 assembly of *Mus musculus* genome using STAR [66] version 2.5.3a (--twopassMode Basic). Gene expression was quantified using htseq-count 0.6.1p1 [67] and gene annotations from Ensembl release 91. Statistical analysis was performed using R 3.3.2 and DESeq2 1.16.1 Bioconductor library [68].

### Golgi staining

GolgiCox staining was performed using the FD Rapid GolgiStain Kit (FD NeuroTechnologies, Ellicott City, MD) on the entire fresh brains, and processed as indicated by the manufacturer. After impregnation, the brains were embedded in 3% low-melting agarose and cut with a vibratome (VT1200S, Leica); 100-µm-thick sections were mounted on gelatine-coated slides and allowed to dry for 2 days before staining. Images were acquired with the Hamamatsu slide scanner at × 40 resolution with multi-layer mode. All the measurements were taken by the same operator, blind to the genotype using the Hamamatsu NDPviewer2.0 software.

### Behavioral experiments

Eleven core phenotypes were tested: (i) anxiety in the open-field and elevated plus maze paradigms; (ii) learning and memory in the Y-maze, the novel object recognition test, the three-chambers paradigm, and the cued and contextual fear conditioning test; (iii) locomotion in the circadian activity test; (iv) epilepsy in the pentylenetetrazol (PTZ)-induced seizure paradigm; (v) coordination in the rotarod performance test; (vi) motricity in the grip strength test; (vii) schizophrenia in the prepulse inhibition and startle reflex test; (viii) autism in the social behavior and marble burying paradigms; (ix) depression in the forced swim and tail suspension tests; (x) anhedonia in the sucrose preference test; and (xi) sociability using the social recognition test. Each test is described in detail in Additional file 9.

### Statistics

Statistical analyses were performed with GraphPad Prism 8.0.2, using two-tailed Student’s *t* tests of equal variances (except for Additional file 1: Fig. S9A-C, *t* tests of unequal variances) and one-way or two-way ANOVA followed by post hoc tests. Tests performed are indicated in the figure legends. The results are reported as box plots with individual data points overlaid as mean ± sample standard error of the mean, except for Fig. 4A, B, E; Additional file 1: Fig. S4; and Additional file 1: Fig. S7A which show the average for simplicity purposes. Figures were prepared using Affinity Designer. Significance was reported as follows: *p < 0.05, **p < 0.01, and ***p < 0.001. All replicates are biological replicates. For qPCR data, delta *C*_*T*_ values were obtained by normalizing *C*_*T*_ values of *Mvp* against two reference genes, *Gnas* and *Hprt*.

### Supplementary Information

**Additional file 1:** **Fig. S1.** Standardized workflow for neurophenotyping. **Fig. S2.** Heat map of gene association with NAPs in males. **Fig. S3.** Heat map of gene association with NAPs in females. **Fig. S4. **Analysis of *Mvp*, *Mapk3 *and *Kctd13 *mice*.***Fig. S5.** *Mvp* expression profiling. **Fig. S6. ***Mvp* knockout model validation and viability screen. **Fig. S7.** Impact of *Mvp* deletion on neuromorphology in females. **Fig. S8.** Impact of *Mvp* deletion on neuromorphology in males. **Fig. S9.** Analysis of *Mvp* loss in the cingulate gyrus. **Fig. S10.** Behavioral studies of *Mvp* homozygous male mice. **Fig. S11.** Behavioral studies of *Mapk3* heterozygous male mice. **Fig. S12.** Behavioral studies of DKO of *Mvp* and *Mapk3* genes.

**Additional file 2:** **Table S1.** Description of mouse models used in the study. **Table S2.** List of 67 neuroanatomical measurements in postnatal mice. **Table S3.** MVP-positive brain structures and link to limbic system. **Table S4.** Expression scores of MVP in murine brain. **Table S5.** List of 42 neuroanatomical measurements in E18.5 embryos. **Table S6.** List of 40 sagittal measurements in postnatal mice.

**Additional file 3:** **Table S7.** Full neuroanatomical data

**Additional file 4:** **Table S8.** Description of gene association with NAPs.

**Additional file 5:Table S9.** Raw data for the various *Mvp *studies.

**Additional file 6:** **Table S10.** Raw behavioral data  for *Mvp*^-/-^.

**Additional file 7:** **Table S11.** Raw behavioural data for *Mapk3*^+/-^.

**Additional file 8:** **Table S12.** Raw behavioral data for *Mvp*^+/-^;*Mapk3*^+/-^.

**Additional file 9:** **Supplementary methods.** In this additional file, we provide detailed information about: 1) study samples and mouse knockout constructions, 2) animal welfare, 3) genotyping and sexing of study samples, 4) brain histological screen, 5) follow-up histology studies of MVP/vault, 6) evaluation of MVP/vault transcript level, 7) immuno-histofluorescence, 8) Western Blot analysis, 9) immuno-cytofluorescence, 10) bulk RNA sequencing, 11)  Golgi staining, 12) electrophysiology, 13) electronic microscopy, and 14) behavioral analysis.

**Additional file 10:** **Supplementary results.** Here we present data of i) male NAP genes implicated in two or more brain categories, ii) male NAP genes implicated in one brain category, iii) female NAP genes involved in two or more brain categories, and iv) female NAP genes involved in one brain category. We also show preliminary data on ERK activity measurements.

**Additional file 11.** Summary table of key resources.

**Additional file 12.** Uncropped images for the cropped blots in Fig. 3 and Additional file 1: Fig S5, S6 and S12.

**Additional file 13.** Review history.

## Data Availability

Detailed information about the datasets generated in this study that support the conclusions of this article is available either in Additional file 2 or in the accompanying Additional files 9, 10, and 11. No custom scripts and software were used other than those mentioned in the “Methods” section. The RNA sequencing data generated in this study have been deposited in the NCBI’s Gene Expression Omnibus (GEO) database and are accessible through GEO Series accession number GSE243261 [69].
