# Supplementary material for: Dissecting the autism-associated 16p11.2 locus identifies multiple drivers in neuroanatomical phenotypes and unveils a male-specific role for the major vault protein
Source: Genome Biol. 2023 Nov 15;24:261. doi: 10.1186/s13059-023-03092-8 (PMC10647150; doi:10.1186/s13059-023-03092-8)
Supplement: Supplementary file 2 — Additional file 2: Table S1. Description of mouse models used in the study. Table S2. List of 67 neuroanatomical measurements in postnatal mice. Table S3. MVP-positive brain structures and link to limbic system. Table S4. Expression scores of MVP in murine brain. Table S5. List of 42 neuroanatomical measurements in E18.5 embryos. Table S6. List of 40 sagittal measurements in postnatal mice. [file 13059_2023_3092_MOESM2_ESM.docx]

ADDITIONAL FILE 2:

Supplementary tables (S1-S6) and the legends for

dataset tables (S7-S12)

**Dissecting the autism-associated 16p11.2 locus identifies multiple drivers in neuroanatomical phenotypes and unveils a male-specific role for the major vault protein**

Perrine F. Kretz^1^, Christel Wagner^1^, Anna Mikhaleva^2^, Charlotte Montillot^3^, Sylvain Hugel^4^, Ilaria Morella^5^, Meghna Kannan^1^, Marie-Christine Fischer^1^, Maxence Milhau^3^, Ipek Yalcin^4^, Riccardo Brambilla^5,6^, Mohammed Selloum^1,7^, Yann Herault^1,7^, Alexandre Reymond^2^, Stephan C. Collins^1,8^ and Binnaz Yalcin^1,8,*^

^1^University of Strasbourg, CNRS, INSERM, Institute of Genetics and Molecular and Cellular Biology, IGBMC, UMR7104, U964, 67400 Illkirch, France

^2^Center for Integrative Genomics, University of Lausanne, CH-1015 Lausanne, Switzerland

^3^Inserm UMR1231, Université de Bourgogne Franche-Comté, 21000 Dijon, France

^4^Institute of Cellular and Integrative neuroscience, UPR3212, CNRS, 67000 Strasbourg, France

^5^Neuroscience and Mental Health Innovation Institute, School of Biosciences, Cardiff University, CF24 4HQ Cardiff, UK

^6^Dipartimento di Biologia e Biotecnologie “Lazzaro Spallanzani”, Università degli Studi di Pavia, Pavia, Italy

^7^University of Strasbourg, CNRS, INSERM, CELPHEDIA, PHENOMIN, ICS, 67400 Illkirch, France.

^8^Current address: Université de Bourgogne, Inserm UMR1231, 21000 Dijon, France

^*^Correspondence: [binnaz.yalcin@inserm.fr](mailto:binnaz.yalcin@inserm.fr)

**Table S1.** Description of mouse models used in the study.

The 30 protein-coding mouse genes encompassing the 16p11.2-syntenic region are listed in column (A). Details are given according to Ensembl GRCm38.p6 and consist of the accession number, the strand (+: forward; - : reverse), the genomic coordinates (base pair), the total length of the gene (base pair) and the full name description (columns B-G, respectively). The 20 genes that underwent the neuroanatomical screen are indicated in column H. Column I provides the complete allele name. The core strain of the mouse is indicated in column J, the origin of the mice in column K (name of the production center or of the collaborator who provided the material) and finally column L the material received (living mouse or dissected brain sample).

**Table S2.** List of 67 neuroanatomical measurements in postnatal mice.

This table enlists combined morphological phenotypes studied in the coronal neuroanatomical screen. Stereotaxic coordinates of the two sections of interest are indicated. Association of each parameter with a brain region is indicated in the 2^nd^ column. The 3^rd^ column shows the number attributed to each region (**Figs. 1 and 4**, **Additional file 1: Fig. S4** and **Additional file 1: Fig. S7**). The 4^th^ column gives the merged name of the parameter (used in **Additional file 4: Table S8**), followed by the full name, the description and the unit the measurement.


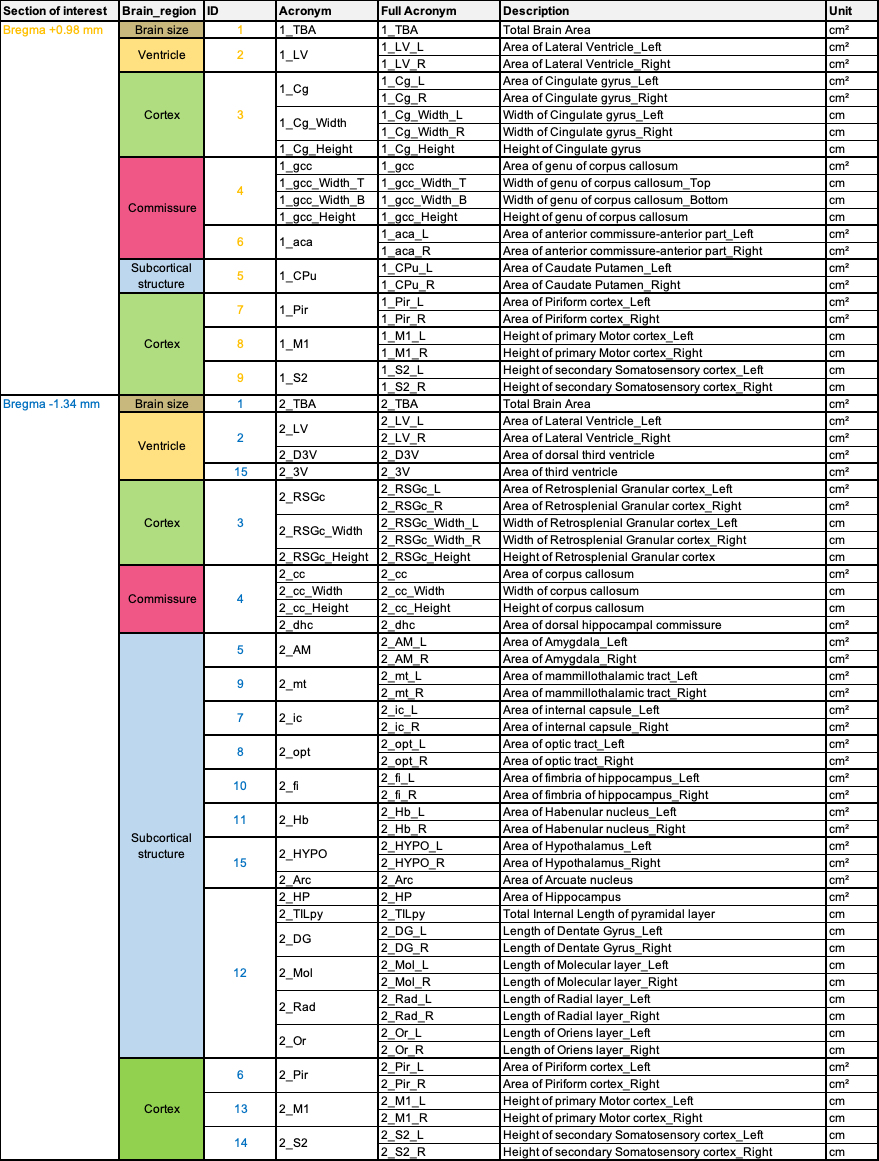


**Table S3.** MVP-positive brain structures and link to limbic system.

MVP-positive regions identified with immunohistofluorescence on wild-type brain sections are indicated in columns A-B and their known function in column C (**Fig. 2**, **Additional file 1: Fig. S5** and **Additional file 2: Table S4**). The link with the limbic system (defined as Established, Putative or None) are indicated in column D. Direct connections between the different MVP-positive structures are summarized in column E. The last column (ref. for Reference) gives the literature sources used to elaborate this table (see last page of this document).

| **A** | **B** | **C** | **D** | **E** | **F** |
| --- | --- | --- | --- | --- | --- |
| **Acronym** | **Description** | **Known function** | **Link to limbic system** | **Known connection between MVP-positive structures** | **Ref** |
| **7N** | Facial nerve nucleus | Facial expression - Salivation | Putative |  |  |
| **10N** | Vagus nerve nucleus | Autonomic modulation | Established | Sol | ^1^ |
| **aca** | anterior commissure - anterior part | Connection between amygdalae, and olfactory system | Established | Pir |  |
| **AcNuc** | Acumbens Nucleus | Processing of rewarding and reinforcing - Regulation of slow wave sleep | Established | f, CPu |  |
| **AP** | Area Postrema | Blood chemoreception - Autonomic control | None | Sol |  |
| **Arc** | Arcuate hypothalamic nucleus | Autonomic control - Homeostasis | None | PVH, Sol, HP, RSGc |  |
| **AV** | Arbor vitae | Cerebellum white matter | None |  |  |
| **cc** | Corpus callosum | Connection of the cerebral cortex | Putative | Cg, S2, RSGc |  |
| **Cg** | Cingulate gyrus | Emotion processing - Learning - Memory | Established | ZI, RM, Raphe, cc, DMT, CMT, MS, DBB | ^2,3^ |
| **CMT** | Central medial thalamic nucleus | Attention modulation | Putative | CPu, MVe, Cg, S2 |  |
| **CN** | Cuneate nucleus | Upper body sensitivity | None |  |  |
| **CP** | Choroid plexus | Cerebrospinal fluid production | None |  |  |
| **CPu** | Caudate-Putamen | Goal directed actions - Motor learning - slow wave sleep - attachment behaviors | Putative | AcNuc, CMT |  |
| **DBB** | Diagonal band of Broca | connects with DG in HP, theta waves with MS | Established | PVH, f, MS, ZI, HP, Cg | ^4^ |
| **Dk** | Darkschewitsch nucleus | Pupillary light reflex | None | ZI, PAG | ^5^ |
| **DMH** | Dorsal medial hypothalamic nucleus | Circadian rhythm - Feeding behavior - Autonomous regulation | None | LPO, PVH |  |
| **DMT** | Dorsal medial thalamic nucleus | Attention modulation - Memory - Emotional pain response - Oculomotor | Established | S2, Cg, HP |  |
| **f** | Fornix | Memory - Theta rhythm | Established | RM, MS, AcNuc, DBB |  |
| **Lith** | Lithoid nucleus |  | None | PAG |  |
| **LPO** | Lateral preoptic area | Modulation of sleep and thirst - Reward-related behaviors | Established | ZI, Raphe, DMH | ^6^ |
| **MS** | Medial septal nucleus | Theta waves generation | Established | PHV, Cg | ^4^ |
| **MVe** | Medial vestibular nucleus | Balance - Vestibular integration | Putative |  | ^7^ |
| **PAG** | Periaqueductal gray | Pain modulation | Established | Dk, Lith |  |
| **Pir** | Piriform cortex | Olfaction | Putative | aca |  |
| **PVH** | Paraventricular hypothalamic nucleus | Autonomic control - Appetite | Established | SFO, Arc, MS, DBB, Sol, ZI, DMH | ^8^ |
| **Raphe** | Raphe | Pain modulation - Thermoregulation - Emotion regulation - Sleep/wake state | Established | Sol, Cg, LPO, TS | ^9^ |
| **RM** | Retromammillary nucleus | Memory - Theta rhythm | Established | f, HP, Cg |  |
| **RSGc** | Retrosplenial cortex | Episodic memory - navigation - slow wave theta rhythmicity | Established | cc, Arc, HP, | ^10^ |
| **S2** | Somatosensory cortex | Integration of somesthesy (light touch, visceral sensation) | Putative | cc, DMT, CMT |  |
| **Sb** | Subiculum | Memory - Addiction | Putative | HP |  |
| **SFO** | Subfornical organ | Energy homeostasis - Cardiovascular modulation | None | PVH |  |
| **Sol** | Solitary nucleus | Autonomic modulation | Established | AP, PVH, Raphe, 10N | ^1^ |
| **TS** | Triangular septal nucleus | Control of anxiety and fear | Established | Raphe | ^11,12^ |
| **vHP** | Hippocampus - ventral part | Memory - Large scale spatial navigation | Established | Arc, DBB, RM, DMT, RSGc, DS | ^13^ |
| **Xi** | Xiphoid thalamic nucleus | Visual threats adaptive response | Putative | Amygdala | ^14^ |
| **ZI** | Zona incerta | Autonomic control - Slow-wave sleep - Attention | Established | Cg, LPO, PVH, DBB, Dk | ^15,16^ |

**Table S4.** Expression scores of MVP in murine brain.

Summary of MVP expression obtained from immunohistofluorescence on serial coronal (from Bregma +1.94mm to Bregma -7.67mm) and sagittal (Lat +0.24mm to Lat +0.72mm) sections. The stereotaxic coordinates are indicated in the first column along with the corresponding plane number of the Franklin and Paxinos Atlas^17^. Each brain part is indicated at the top of each table, and more precision about the identity of each structure is indicated in bracket. Abbreviations of MVP-positive structures are given in **Additional file 2: Table S3**. MVP/vault expression is scored as mild (+), moderate (++) or strong (+++) across all the positive brain regions. ¶ indicate the structure where only fibers are studied. NA: not applicable. NV: not visible. Cells highlighted in pale blue correspond to illustrations presented in **Figure 2** and **Additional file 1: Fig. S5**.

**Table S5.** List of 42 neuroanatomical measurements in E18.5 embryos.

This table enlists morphological phenotypes, adapted from the postnatal neuroanatomical screen, to fit to the analysis of brain anatomy in mouse embryo at embryonic stage 18.5 (E18.5). Stereotaxic coordinates (column A) of the two sections of interest are indicated. Column B gives the merged name of the parameter (used in **Additional file 4: Table S8**, column C the full name, column D the description and column E the unit of the measurement. An illustrative representation of the measurements is available in **Additional file 9: Fig. S15**.

**Table S6.** List of 40 sagittal measurements in postnatal mice.

This table enlists combined morphological phenotypes studied on sagittal plane. Stereotaxic coordinates (column A) of the section of interest are indicated. Column B shows the number attributed to each region in **Figure 4**. Column C gives the full name, column D the description and column E the unit of the measurement.

**Table S7.** Full neuroanatomical data.

A total of 458 samples were processed and analyzed across two coronal sections. A unique barcode (column A) identifies each sample. Column B and C refer to each subproject that were used to create subgroups for the analysis. Columns D to N give additional elements about study samples. Columns O to CC are the raw data for the 67 brain parameters (see **Additional file 2: Table S2** for more information about the parameters), columns CD and CE give body and brain weight, and columns CF to DO provide the co-variates for each sample.

**Table S8.** Description of gene association with NAPs.

**Sheet 1** HeatMap_SingleGenes_Coronal.

Columns A-I provide general information about each group of mice analyzed in the histology study. More specifically, column A gives the gene name, column B the core background strain, column C the full allele name, column D the age of the mice at necropsy in weeks, column E the gender of studied mice, column F the zygosity, column G the number of mutant mice and column I the number of colony-matched control animals (column H). Column J indicates the significance threshold of the NAP gene defined as NAT (NeuroAnatomical Threshold), column K the directionality of the impact on gene structure, column L the number of neuroanatomical phenotypes at the defined NAT of 0.05, column M the type of the statistics and finally columns N-BC the p-value and percentage change for each brain parameter across each tested allele. Column BD and BE indicate p-value and percentage change for body and brain weight, respectively. Column BF to BK give the number of NAPs according to each NAT. Column BL to BR give additional descriptive statistics. A color-code is used to indicate p-value threshold, ranging from yellow (for 0.05) to red for stringent threshold (of 0.0001) and directionality of percentage change (blue indicates decrease and red increase).

**Sheet 2** HeatMap_Mvp_Dev.

Column A to BC and color-code legend are the same as **Sheet 1**. Column BD to BI give the number of NAPs according to each NAT.

**Sheet 3** HeatMap_Mvp_Sagittal.

Column A to M and color-code legend are the same as **Sheet 1**. Columns N-BA indicate the p-value and percentage change for each brain parameter across each tested allele. Column BB to BG give the number of NAPs according to each NAT.

**Table S9.** Raw data for the various *Mvp* studies.

**Sheet 1** Viability: Calculation of birth ratio for each genotype, and each sex, done on successfully genotyped mice obtained from heterozygous by heterozygous breeding (n=617).

**Sheet 2** WesternBlot: Measurements of MVP and Actin levels in liver samples from *Mvp* mutant mice.

**Sheet 3** Dev_E18.5: Raw data from male and female *Mvp^+/+^* and *Mvp^-/-^* E18.5 embryos. See embryos. Extended for description of the parameters.

**Sheet 4** Histocount_Coro_M: Cellular parameters for male *Mvp* mutants. Cellular measures done on histological coronal sections (see **Additional file 2: Table S2** for corresponding parameter description) are presented in the table. For 1_M1, 1_S2 and 2_S2 analysis of area and cellular parameters was further explored in the different cortical layers (**Additional file 9: Supplementary methods** for more explanation). Columns highlighted in purple designate the data obtained by calculation: avgcellarea=cellarea/cellcount; cellareapercent=cellarea/structure_area; celldens=cellcount/structure_area.

**Sheet 5** Histocount_Coro_F: Cellular parameters for female *Mvp* mutants. Measures done on histological coronal sections on specific regions (see **Additional file 2: Table S2** for corresponding parameter description) are presented in the table. Columns highlighted in purple designate the data obtained by calculation: avgcellarea=cellarea/cellcount; cellareapercent=cellarea/structure_area; celldens=cellcount/structure_area.

**Sheet 6** Histocount_Sag_M: Cellular parameters for male *Mvp* mutants. Measures done on histological sagittal sections on motor cortex (4_M1) and cerebellar lobes (Cb) are presented in the table. For Cb, the length of granular layer for each lobule (GLlength, in cm) and the number of Purkinje cells (PC) was measured (see **Additional file 1: Fig. S8H** for further explanation). Columns highlighted in purple designate the data obtained by calculation: avgcellarea=cellarea/cellcount; cellareapercent=cellarea/M1_area; celldens=cellcount/M1_area for motor cortex and PCdensity=PC/GL_length. P-value of Student *t*-test and percentage change of each interaction and the mean and SEM for each genotype are indicative.

**Sheets 7-13** Immunocytofluorescence (ICF): these tabs recapitulates the data obtained by the measurements of cellular parameters done on primary hippocampal neuronal cultures at DIV4 from E18.5 male and female embryos. Soma area (soma), growth cone (GC) area and axonal length (AL) are presented (see **Fig. 4F** for visual description). The raw measure for each neuron is given in column D (area (in µm^2^) for soma and GC, and length (in µm) for AL). The decimal logarithm (Log10) of each measure is given in column E and is used to conduct statistical tests. For male soma, two different batches were tested, each reported in a different tab.

**Sheet 14** Golgi_SpineDensity: Spine density in somatosensory neurons. Column A and B inform on the number and the genotype of the mouse. The length measured on analyzed portion of dendrite, as well as the number of spines counted are provided in column C and D. Spine density (E) corresponds to : SpineDensity = #spines/length.

**Sheet 15.** Electrophysiology. Patch-clamp data in cingulate gyrus. Inter-event interval (IEI, in ms) and amplitude (in pA) of mEPSCs are recorded. The decimal logarithm (Log10) of each measure is given and was used to conduct statistical tests. The capacitance of each neuron is measured.

**Table S10.** Raw behavioral data for *Mvp^-/-^*.

Raw data sheets for the analysis of *Mvp^-/-^* mutant mice (n=12) versus WT (n=10) mice. See **Additional file 9: Supplementary methods** for more detailed description of test procedures.

**Sheet 1** **Legend.**

**Sheet 2** **Mvp_OF.** Open Field raw data. Measured parameters: Distance Traveled (cm), Number of rearing, % Time Spent in the Center, Distance (cm) in Center, Latency (s) in Center and % Distance in Center. Data are calculated over five minutes periods along the test (max 30min).

**Sheet 3** **Mvp_EPM.** Elevated Plus Maze. Measured parameters: number of entries and time spent in closed or open arms, number of head dip, rearing and extension, and latency (s) before entering open arm.

**Sheet 4** **Mvp_FC_Cond.** Fear Conditioning – Conditioning session. Immobility duration measured over three consecutive periods of two minutes (Hab-1, Hab-2 and Post-US1). Electrical foot shock, preceded by light and tone stimuli is given right before Post-US1.

**Sheet 5 Mvp_FC_Context.** Fear Conditioning – Context session. Duration of immobility (s) of the mouse during three consecutive periods of two minutes. Percentage of freezing over the total time (360s) is calculated and indicated.

**Sheet 6** **Mvp_FC_Cue.** Fear Conditioning – Cue session. Immobility (s) of the mouse measured over four consecutive periods of two minutes (Pre-Cue1, Cue1, Pre-Cue2 and Cue2). Percentage of freezing during Cue1 and Cue2 is calculated on the duration of the session.

**Sheet 7** **Mvp_TST.** Tail Suspension Test. Immobility (s) of the mouse measured over three consecutive periods of two minutes (Block1, Block2 and Block3) after suspension by the tail. Latency (s) to immobility is also recorded.

**Sheet 8** **Mvp_PTZ.** PTZ-susceptibility test. Latency and duration of the clonic and tonic phases of status epilepticus after PTZ intraperitoneal injection.

**Sheet 9** **Mvp_Rotarod_Learning.** Rotarod test – Learning session. Time (in seconds) spent on the rotarod at increasing speed from 4 to 40 rotations per minute (rpm) over five minutes session, during four sessions on three consecutive days.

**Sheet 10** **Mvp_Rotarod_Test.** Rotarod test – Test session. Latency before falling off the rotarod at rotation speeds (4, 10, 16, 22, 28, 34 and 40 rpm). Maximum per trial is set to 120 seconds. Two trials were done and the mean of both was calculated and gathered in the bottom panel.

**Sheet 11** **Mvp_SP_Data.** Sucrose preference test – Test data. Weight of water and 0.8% sucrose bottles before and after each session. Consumption corresponds to the difference between the two weights. Sucrose preference was calculated as the percentage of sucrose consumption over the total consumption during habituation (Hab, 1h), and testing days (Test-Day1 and Test–Day2, 12 hours during dark cycle).

**Sheet 12** **Mvp_SP_Consumption.** Sucrose preference test – Consumption data. Beverage consumption (addition of sucrose and water consumption) for the three sessions.

**Sheet 13** **Mvp_YM.** . Y-Maze test. Poss (Poss=number of total visited arm - 2) is used as an alias of locomotor activity, Real (Real: three consecutive different arms visited) and percentage of alternation (%Alt) as an alias for working memory. %Alt = Real/Poss*100. Same Arm Return (SAR) and Alternance Arm Return (AAR) were recorded and percentage calculated. Test was conducted on a single trial and data over either 6 or 8 minutes were transcribed separately.

**Sheet 14** **Mvp_NOR.** Novel-Object recognition test. Exploration time (s) of the different objects (ObjectA in 1st session, and ObjectFam and ObjectNew, in 2nd session). Discrimination ratio = (ObjectNew – ObjectFam)/(ObjectNew+ObjectFam). Percentage of exploration of the new object over total object exploration %Tnew=ObjectNew/(ObjectNew+ObjectFam). 50% was considered as no recognition of the familiar object.

**Sheet 15-17** **Mvp_SR_Phase1/2/3.** Social Recognition – Phase 1/2/3. Total distance moved (cm) during each phase of SR test. Cumulative duration of the mouse localization in each of the three-chamber (Left, Center or Right) either Zone1 (the entire chamber), Zone2 (closer to the goal box).

**Sheet 18** **Mvp_Marbles.** Marbles burying test. Number of marbles that were ¾ covered, completely covered or not covered after 15minutes of test period, and their relative percentage.

**Sheet 19** **Mvp_CA_All.** Tracking of locomotor activity (in centimeters) every hour over the duration of the test (32 hours). Statistical analysis was performed over the combined Dark and Light phase (from 7pm to 7pm). Back and front activity, as well as rearing, licks and pellet distribution are also recorded.

**Sheet 20** **Mvp_CA_food consumption.** Circadian consumption. Weight of pellets and water in grams consumed over 32 hours. The difference between pellet distribution and pellet lost is considered as pellet distribution. The weight of water bottles before and after the trial was also measured to quantify water consumption.

**Sheet 21** **Mvp_PPI_Avg.** Pre-Pulse Inhibition test – Average value. Averaged amplitude of the acoustic startle reflex (arbitrary unit) following 60 dB (Background Noise = BN), 70 dB pulse (P70), 75 dB (P75), 85 dB (P85), 90 dB (P90) and 110 dB (ST110) acoustic stimulus. PPI: Percentage of Pre-Pulse (PP) Inhibition calculated as PPX %=( PPX-ST110)/ST110.

**Sheet 22** **Mvp_PPI_Max.** Pre-Pulse Inhibition test – Max value. Maximum amplitude of the acoustic startle reflex (arbitrary unit) following 60 dB (Background Noise = BN), 70 dB pulse (P70), 75 dB (P75), 85 dB (P85), 90 dB (P90) and 110 dB (ST110) acoustic stimulus. PPI: Percentage of Pre-Pulse (PP) Inhibition calculated as PPX %=( PPX-ST110)/ST110.

**Table S11.** Raw behavioural data for *Mapk3*^+/-^.

Raw data sheets for the analysis of *Mapk3*^+/-^ mutant mice (n=12) versus WT (n=12) mice in two different cohorts. See **Additional file 9: Supplementary methods** more detailed description of test procedures.

**Sheet 1** **Legend**

**Sheet 2** **Mapk3_C1_OF.** Open Field raw data. Measured parameters: Distance Traveled (cm), Number of rearing, % Time Spent in the Center, Distance (cm) in Center, Latency (s) in Center and % Distance in Center. Data are calculated over five minutes periods along the test (max 30min).

**Sheet 3** **Mapk3_C1_EPM.** Elevated Plus Maze. Measured parameters: number of entries and time spent in closed or open arms, number of head dip, rearing and extension, and latency (s) before entering open arm.

**Sheet 4** **Mapk3_C1_FC_Cond.** Fear Conditioning – Conditioning session. Immobility duration measured over three consecutive periods of two minutes (Hab-1, Hab-2 and Post-US1). Electrical foot shock, preceded by light and tone stimuli is given right before Post-US1.

**Sheet 5** **Mapk3_C1_FC_Context.** Fear Conditioning – Context session. Duration of immobility (s) of the mouse during three consecutive periods of two minutes. Percentage of freezing over the total time (360s) is calculated and indicated.

**Sheet 6** **Mapk3_C1_FC_Cue.** Fear Conditioning – Cue session. Immobility (s) of the mouse measured over four consecutive periods of two minutes (Pre-Cue1, Cue1, Pre-Cue2 and Cue2). Percentage of freezing during Cue1 and Cue2 is calculated on the duration of the session.

**Sheet 7-8** **Mapk3_C1/C2_TST**. Tail Suspension Test for cohort 1/2. Immobility (s) of the mouse measured over three consecutive periods of two minutes (Block1, Block2 and Block3) after suspension by the tail. Latency (s) to immobility is also recorded.

**Sheet 9** **Mapk3_C2_PTZ.** PTZ-susceptibility test. Latency and duration of the clonic and tonic phases of status epilepticus after PTZ intraperitoneal injection.

**Sheet 10** **Mapk3_C2_Rotarod_Learning.** Rotarod test – Learning session. Time (in seconds) spent on the rotarod at increasing speed from 4 to 40 rotations per minute (rpm) over five minutes session, during four sessions on three consecutive days.

**Sheet 11** **Mapk3_C2_Rotarod_Test.** Rotarod test – Test session. Latency before falling off the rotarod at rotation speeds (4, 10, 16, 22, 28, 34 and 40 rpm). Maximum per trial is set to 120 seconds. Two trials were done and the mean of both was calculated and gathered in the bottom panel.

**Sheet 12** **Mapk3_C2_SP_Data.** Sucrose preference test – Test data. Weight of water and 0.8% sucrose bottles before and after each session. Consumption corresponds to the difference between the two weights. Sucrose preference was calculated as the percentage of sucrose consumption over the total consumption during habituation (Hab, 1h), and testing days (Test-Day1 and Test–Day2, 12 hours during dark cycle).

**Sheet 13** **Mapk3_C2_SP_Consumption.** Sucrose preference test – Consumption data. Beverage consumption (addition of sucrose and water consumption) for the three sessions.

**Sheet 14** **Mapk3_C1_YM.** Y-Maze test. Poss (Poss=number of total visited arm - 2) is used as an alias of locomotor activity, Real (Real: three consecutive different arms visited) and percentage of alternation (%Alt) as an alias for working memory. %Alt = Real/Poss*100. Same Arm Return (SAR) and Alternance Arm Return (AAR) were recorded and percentage calculated. Test was conducted on a single trial and data over either 6 or 8 minutes were transcribed separately.

**Sheet 15** **Mapk3_C1_NOR.** Novel-Object recognition test. Exploration time (s) of the different objects (ObjectA in 1st session, and ObjectFam and ObjectNew, in 2nd session). Discrimination ratio = (ObjectNew – ObjectFam)/ (ObjectNew+ObjectFam). Percentage of exploration of the new object over total object exploration %Tnew=ObjectNew/ (ObjectNew+ObjectFam). 50% was considered as no recognition of the familiar object.

**Sheet 16-18** **Mapk3_C1_SR_Phase1/2/3.** Social Recognition – Phase 1/2/3. Total distance moved (cm) during each phase of SR test. Cumulative duration of the mouse localization in each of the three-chamber (Left, Center or Right) either Zone1 (the entire chamber), Zone2 (closer to the goal box).

**Sheet 19** **Mapk3_C1_Marbles.** Marbles burying test. Number of marbles that were ¾ covered, completely covered or not covered after 15minutes of test period, and their relative percentage.

**Sheet 20** **Mapk3_C1_CA_All**. Tracking of locomotor activity (in centimeters) every hour over the duration of the test (32 hours). Statistical analysis was performed over the combined Dark and Light phase (from 7pm to 7pm). Back and front activity, as well as rearing, licks and pellet distribution are also recorded.

**Sheet 21** **Mapk3_C1_CA_Consumption.** Circadian consumption. Weight of pellets and water in grams consumed over 32 hours. The difference between pellet distribution and pellet lost is considered as pellet distribution. The weight of water bottles before and after the trial was also measured to quantify water consumption.

**Sheet 22** **Mapk3_C1_PPI_Avg.** Pre-Pulse Inhibition test – Average value. Averaged amplitude of the acoustic startle reflex (arbitrary unit) following 60 dB (Background Noise = BN), 70 dB pulse (P70), 75 dB (P75), 85 dB (P85), 90 dB (P90) and 110 dB (ST110) acoustic stimulus. PPI: Percentage of Pre-Pulse (PP) Inhibition calculated as PPX %=( PPX-ST110)/ST110.

**Sheet 23** **Mapk3_C1_PPI_Max.** Pre-Pulse Inhibition test – Max value. Maximum amplitude of the acoustic startle reflex (arbitrary unit) following 60 dB (Background Noise = BN), 70 dB pulse (P70), 75 dB (P75), 85 dB (P85), 90 dB (P90) and 110 dB (ST110) acoustic stimulus. PPI: Percentage of Pre-Pulse (PP) Inhibition calculated as PPX %=( PPX-ST110)/ST110.

**Table S12.** Raw behavioral data for *Mvp^+/-^*;*Mapk3^+/-^*.

Raw data sheets for the analysis of *Mvp::Mapk3* double-heterozygotes (n=12) versus WT (n=12) mice. See **Additional file 9: Supplementary methods** for more detailed description of test procedures.

**Sheet 1** **Legend**

**Sheet 2** **DblKO_BW**. Body weight longitudinal study. Weight were taken every week between 11 and 26 weeks old.

**Sheet 3** **DblKO_OF.** Open Field raw data. Measured parameters: Distance Traveled (cm), Number of Rearing’s, % Time Spent in the Center, Distance (cm) in Center, Latency (s) in Center and % Distance in Center. Data are calculated over five minutes periods along the test (max 30min).

**Sheet 4** **DblKO_EPM.** Elevated Plus Maze. Measured parameters: number of entries and time spent in closed or open arms, number of head dip, rearing and extension, and latency (s) before entering open arm.

**Sheet 5** **DblKO_FC_Cond.** Fear Conditioning – Conditioning session. Immobility duration measured over three consecutive periods of two minutes (Hab-1, Hab-2 and Post-US1). Electrical foot shock (US), preceded by light and tone stimuli is given right before Post-US1.

**Sheet 6** **DblKO_FC_Cont.** Fear Conditioning – Context session. Duration of immobility (s) of the mouse during three consecutive periods of two minutes. Percentage of freezing over the total time (360s) is calculated and indicated.

**Sheet 7** **DblKO_FC_Cue.** Fear Conditioning – Cue session. Immobility (s) of the mouse measured over four consecutive periods of two minutes (Pre-Cue1, Cue1, Pre-Cue2 and Cue2). Percentage of freezing during Cue1 and Cue2 is calculated on the duration of the session.

**Sheet 8** **DblKO_TST.** Tail Suspension Test. Immobility (s) of the mouse measured over three consecutive periods of two minutes (Block1, Block2 and Block3) after suspension by the tail. Latency (s) to immobility is also recorded.

**Sheet 9** **DblKO_FS.** Forced Swim test. Immobility (s) of the mouse measured over three consecutive periods of two minutes (Trial1, Trial2 and Trial3) after immersion in a becher of water. Latency (s) to immobility is also recorded.

**Sheet 10** **DblKO_PTZ.** PTZ-susceptibility test. Latency and duration of the clonic and tonic phases of status epilepticus after PTZ intraperitoneal injection.

**Sheet 11** **DblKO_GS.** Grip Strength. Grip strength values for four or two paws in kg and g. Each condition is repeated 4 times. The mean and the maximal value (highlighted in orange) are calculated and normalized over the mouse body weight.

**Sheet 12** **DblKO_Rotarod_Learning.** Rotarod test – Learning session. Time (in seconds) spent on the rotarod at increasing speed from 4 to 40 rotations per minute (rpm) over five minutes session, during four sessions on three consecutive days.

**Sheet 13** **DblKO_Rotarod_Test.** Rotarod test – Test session. Latency before falling off the rotarod at rotation speeds (4, 10, 16, 22, 28, 34 and 40 rpm). Maximum per trial is set to 120 seconds. Two trials were done and the mean of both was calculated and gathered in the bottom panel.

**Sheet 14** **DblKO_SP_Data.** Sucrose preference test – Test data. Weight of water and 0.8% sucrose bottles before and after each session. Consumption corresponds to the difference between the two weights. Sucrose preference was calculated as the percentage of sucrose consumption over the total consumption during habituation (Hab, 1h), and testing days (Test-Day1 and Test–Day2, 12 hours during dark cycle).

**Sheet 15** **DblKO_SP_Consumption.** Sucrose preference test – Consumption data. Beverage consumption (addition of sucrose and water consumption) for the three sessions.

**Sheet 16** **DblKO_YM.** Y-Maze test. Poss (Poss=number of total visited arm - 2) is used as an alias of locomotor activity, Real (Real: three consecutive different arms visited) and percentage of alternation (%Alt) as an alias for working memory. %Alt = Real/Poss*100. Same Arm Return (SAR) and Alternance Arm Return (AAR) were recorded and percentage calculated. Test was conducted on a single trial and data over either 6 or 8 minutes were transcribed separately.

**Sheet 17** **DblKO_NOR.** Novel-Object recognition test. Exploration time (s) of the different objects (ObjectA in 1st session, and ObjectFam and ObjectNew, in 2nd session). Discrimination ratio = (ObjectNew – ObjectFam)/ (ObjectNew+ObjectFam). Percentage of exploration of the new object over total object exploration %Tnew=ObjectNew/(ObjectNew+ObjectFam). 50% was considered as no recognition of the familiar object.

**Sheet 18-20** **DblKO_SR_Phase1/2/3.** Social Recognition – Phase 1/2/3. Total distance moved (cm) during each phase of SR test. Cumulative duration of the mouse localization in each of the three-chamber (Left, Center or Right) either Zone1 (the entire chamber), Zone2 (closer to the goal box).

**Sheet 21** **DblKO_SI.** Social interaction test. Recordings of the number and time spent to do different social behaviors: Agressivity, Pawing contact, Individual contact, following and sniffing over the ten minutes of the test.

**Sheet 22** **DblKO_Marbles.** Marbles burying test. Number of marbles that were ¾ covered, completely covered or not covered after 15minutes of test period, and their relative percentage.

**Sheet 23** **DblKO_CA_All.** Tracking of locomotor activity (in centimeters) every hour over the duration of the test (32 hours). Statistical analysis was performed over the combined Dark and Light phase (from 7pm to 7pm). Back and front activity, as well as rearing, licks and pellet distribution are also recorded.

**Sheet 24** **DblKO_CA_food consumption.** Circadian consumption. Weight of pellets and water in grams consumed over 32 hours. The difference between pellet distribution and pellet lost is considered as pellet distribution. The weight of water bottles before and after the trial was also measured to quantify water consumption.

**Sheet 25** **DblKO_PPI_Results Avg.** Pre-Pulse Inhibition test – Average value. Averaged amplitude of the acoustic startle reflex (arbitrary unit) following 60 dB (Background Noise = BN), 70 dB pulse (P70), 75 dB (P75), 85 dB (P85), 90 dB (P90) and 110 dB (ST110) acoustic stimulus. PPI: Percentage of Pre-Pulse (PP) Inhibition calculated as PPX %=( PPX-ST110)/ST110.

**Sheet 26** **DblKO_PPI_Results Max.** Pre-Pulse Inhibition test – Max value. Maximum amplitude of the acoustic startle reflex (arbitrary unit) following 60 dB (Background Noise = BN), 70 dB pulse (P70), 75 dB (P75), 85 dB (P85), 90 dB (P90) and 110 dB (ST110) acoustic stimulus. PPI: Percentage of Pre-Pulse (PP) Inhibition calculated as PPX %=( PPX-ST110)/ST110.

**References**

1. Mulkey, S. B. & du Plessis, A. J. Autonomic nervous system development and its impact on neuropsychiatric outcome. *Pediatr. Res.* **85**, 120–126 (2019).

2. Fillinger, C., Yalcin, I., Barrot, M. & Veinante, P. Afferents to anterior cingulate areas 24a and 24b and midcingulate areas 24a’ and 24b’ in the mouse. *Brain Struct. Funct.* **222**, 1509–1532 (2017).

3. Fillinger, C., Yalcin, I., Barrot, M. & Veinante, P. Efferents of anterior cingulate areas 24a and 24b and midcingulate areas 24a’ and 24b’ in the mouse. *Brain Struct. Funct.* **223**, 1747–1778 (2018).

4. Tsanov, M. Speed and Oscillations: Medial Septum Integration of Attention and Navigation. *Front. Syst. Neurosci.* **11**, 67 (2017).

5. Onodera, S. & Hicks, T. P. Projections from substantia nigra and zona incerta to the cat’s nucleus of Darkschewitsch. *J. Comp. Neurol.* **396**, 461–482 (1998).

6. Gordon-Fennell, A. G. *et al.* The Lateral Preoptic Area: A Novel Regulator of Reward Seeking and Neuronal Activity in the Ventral Tegmental Area. *Front. Neurosci.* **13**, 1433 (2019).

7. Balaban, C. D. Neurotransmitters in the vestibular system. *Handb. Clin. Neurol.* **137**, 41–55 (2016).

8. Jiang, Z., Rajamanickam, S. & Justice, N. J. CRF signaling between neurons in the paraventricular nucleus of the hypothalamus (PVN) coordinates stress responses. *Neurobiol. Stress* **11**, 100192 (2019).

9. Teissier, A., Soiza-Reilly, M. & Gaspar, P. Refining the Role of 5-HT in Postnatal Development of Brain Circuits. *Front. Cell. Neurosci.* **11**, 139 (2017).

10. Milczarek, M. M. & Vann, S. D. The retrosplenial cortex and long-term spatial memory: from the cell to the network. *Curr. Opin. Behav. Sci.* **32**, 50–56 (2020).

11. Sperlágh, B., Maglóczky, Z., Vizi, E. S. & Freund, T. F. The triangular septal nucleus as the major source of ATP release in the rat habenula: a combined neurochemical and morphological study. *Neuroscience* **86**, 1195–1207 (1998).

12. Yamaguchi, T., Danjo, T., Pastan, I., Hikida, T. & Nakanishi, S. Distinct roles of segregated transmission of the septo-habenular pathway in anxiety and fear. *Neuron* **78**, 537–544 (2013).

13. Jung, M. W., Wiener, S. I. & McNaughton, B. L. Comparison of spatial firing characteristics of units in dorsal and ventral hippocampus of the rat. *J. Neurosci. Off. J. Soc. Neurosci.* **14**, 7347–7356 (1994).

14. Salay, L. D., Ishiko, N. & Huberman, A. D. A midline thalamic circuit determines reactions to visual threat. *Nature* **557**, 183–189 (2018).

15. Zhou, M. *et al.* A central amygdala to zona incerta projection is required for acquisition and remote recall of conditioned fear memory. *Nat. Neurosci.* **21**, 1515–1519 (2018).

16. Wang, X., Chou, X.-L., Zhang, L. I. & Tao, H. W. Zona Incerta: An Integrative Node for Global Behavioral Modulation. *Trends Neurosci.* **43**, 82–87 (2020).

17. Paxinos, G. & Franklin. *The Mouse Brain in Stereotaxic Coordinates. 3rd ed.* (Academic Press, San Diego, 2007).
