# Supplementary material for: Dissecting the autism-associated 16p11.2 locus identifies multiple drivers in neuroanatomical phenotypes and unveils a male-specific role for the major vault protein
Source: Genome Biol. 2023 Nov 15;24:261. doi: 10.1186/s13059-023-03092-8 (PMC10647150; doi:10.1186/s13059-023-03092-8)
Supplement: Supplementary file 9 — Additional file 9: Supplementary methods. In this additional file, we provide detailed information about: 1) study samples and mouse knockout constructions, 2) animal welfare, 3) genotyping and sexing of study samples, 4) brain histological screen, 5) follow-up histology studies of MVP/vault, 6) evaluation of MVP/vault transcript level, 7) immuno-histofluorescence, 8) Western Blot analysis, 9) immuno-cytofluorescence, 10) bulk RNA sequencing, 11) Golgi staining, 12) electrophysiology, 13) electronic microscopy, and 14) behavioral analysis. [file 13059_2023_3092_MOESM9_ESM.docx]

ADDITIONAL FILE 9:

Supplementary methods

**Dissecting the autism-associated 16p11.2 locus identifies multiple drivers in neuroanatomical phenotypes and unveils a male-specific role for the major vault protein**

Perrine F. Kretz^1^, Christel Wagner^1^, Anna Mikhaleva^2^, Charlotte Montillot^3^, Sylvain Hugel^4^, Ilaria Morella^5^, Meghna Kannan^1^, Marie-Christine Fischer^1^, Maxence Milhau^3^, Ipek Yalcin^4^, Riccardo Brambilla^5,6^, Mohammed Selloum^1,7^, Yann Herault^1,7^, Alexandre Reymond^2^, Stephan C. Collins^1,8^ and Binnaz Yalcin^1,8,*^

^1^University of Strasbourg, CNRS, INSERM, Institute of Genetics and Molecular and Cellular Biology, IGBMC, UMR7104, U964, 67400 Illkirch, France

^2^Center for Integrative Genomics, University of Lausanne, CH-1015 Lausanne, Switzerland

^3^Inserm UMR1231, Université de Bourgogne Franche-Comté, 21000 Dijon, France

^4^Institute of Cellular and Integrative neuroscience, UPR3212, CNRS, 67000 Strasbourg, France

^5^Neuroscience and Mental Health Innovation Institute, School of Biosciences, Cardiff University, CF24 4HQ Cardiff, UK

^6^Dipartimento di Biologia e Biotecnologie “Lazzaro Spallanzani”, Università degli Studi di Pavia, Pavia, Italy

^7^University of Strasbourg, CNRS, INSERM, CELPHEDIA, PHENOMIN, ICS, 67400 Illkirch, France.

^8^Current address: Université de Bourgogne, Inserm UMR1231, 21000 Dijon, France

^*^Correspondence: [binnaz.yalcin@inserm.fr](mailto:binnaz.yalcin@inserm.fr)

Table of content

[1 Study samples and mouse knockout constructions 3](#_Toc63940598)

[1.1 Single-gene knockout 3](#_Toc63940599)

[1.2 Double knockout model 6](#_Toc63940600)

[1.3 Mouse model of the 16p11.2 locus 7](#_Toc63940601)

[2 Animal welfare 8](#_Toc63940602)

[3 Genotyping and sexing of study samples 8](#_Toc63940603)

[4 Brain histological screen 10](#_Toc63940604)

[4.1 Sample processing 10](#_Toc63940605)

[4.2 Histomorphometric analyses and quality control 12](#_Toc63940606)

[4.3 Statistics 13](#_Toc63940607)

[5 Follow-up histology studies of MVP/vault 14](#_Toc63940608)

[5.1 Embryonic stage 14](#_Toc63940609)

[5.2 Sagittal orientation 15](#_Toc63940610)

[5.3 Determination of layers in somatosensory and motor cortices 15](#_Toc63940611)

[5.4 Measurement of cellular features 16](#_Toc63940612)

[6 Evaluation of MVP/vault transcript level 17](#_Toc63940613)

[6.1 Tissue dissection 17](#_Toc63940614)

[6.2 RNA extraction 17](#_Toc63940615)

[6.3 Qualitative RT-PCR 18](#_Toc63940616)

[6.4 Quantitative RT-PCR (qRT-PCR) 19](#_Toc63940617)

[7 Immunohistofluorescence 20](#_Toc63940618)

[7.1 Sample processing 20](#_Toc63940619)

[7.2 MVP/Vault immunostaining 20](#_Toc63940620)

[7.3 Phospho-ERK immunohistofluorescence 21](#_Toc63940621)

[8 Western Blot Analysis 21](#_Toc63940622)

[9 Immunocytofluorescence 22](#_Toc63940623)

[9.1 Primary neuronal cultures 22](#_Toc63940624)

[9.2 Immunocytostaining 23](#_Toc63940625)

[9.3 Cell morphological assessment 24](#_Toc63940626)

[10 RNA sequencing 24](#_Toc63940627)

[11 Golgi staining 25](#_Toc63940628)

[12 Electrophysiology 26](#_Toc63940629)

[12.1 Preparation of acute slices 26](#_Toc63940630)

[12.2 Electrophysiological recordings 26](#_Toc63940631)

[13 Electronic microscopy 27](#_Toc63940632)

[14 Behavioral Analysis 27](#_Toc63940633)

[14.1 Behavioral pipeline 28](#_Toc63940634)

[14.2 Open field 29](#_Toc63940635)

[14.3 Elevated plus maze 30](#_Toc63940636)

[14.4 Fear conditioning 30](#_Toc63940637)

[14.5 Forced swim 31](#_Toc63940638)

[14.6 Tail suspension test 31](#_Toc63940639)

[14.7 Pentylenetetrazol seizure test 31](#_Toc63940640)

[14.8 Grip strength test 32](#_Toc63940641)

[14.9 Rotarod 32](#_Toc63940642)

[14.10 Sucrose preference 32](#_Toc63940643)

[14.11 Y maze 33](#_Toc63940644)

[14.12 Novel object recognition 33](#_Toc63940645)

[14.13 Social recognition 34](#_Toc63940646)

[14.14 Social behavior 34](#_Toc63940647)

[14.15 Marbles burying 34](#_Toc63940648)

[14.16 Circadian activity 35](#_Toc63940649)

[14.17 Acoustic startle response and pre-pulse inhibition (PPI) 35](#_Toc63940650)

[14.18 Statistics 36](#_Toc63940651)

[Supplementary References 38](#_Toc63940658)

## 1 Study samples and mouse knockout constructions

### 1.1 Single-gene knockout

The mouse syntenic 16p11.2 region *Sult1a*-*Spn* encompasses 30 protein-coding genes on chromosome 7: *Sult1a* (new name *Sut1a1*), *Slx1b*, *Bola2*, *Coro1a*, *Mapk3*, *Gdpd3*, *Ypel3*, *Tbx6*, *Ppp4c*, *Aldoa*, *Fam57b*, *4930451I11Rik* (previous name *C16orf92*), *Doc2a*, *Ino80e*, *Hirip3*, *Taok2*, *Tmem219*, *Kctd13*, *Asphd1*, *Sez6l2*, *Cdipt*, *Mvp*, *Pagr1a* (previous name *2900092E17Rik*), *Prrt2*, *Maz*, *Kif22*, *Zg16*, *AI467606*, *Qprt* and *Spn*. A description of each gene is provided in **Additional file 2: Table S1** (columns A-G) including unique accession number based on the mouse genome browser Ensembl (<https://www.ensembl.org/Mus_musculus/>) assembly number GRCm38.p6, chromosome 7 strand, start and end position of the gene of interest, genomic length in base pairs and a description of the gene.

Among these 30 protein-coding genes, 20 (see columns H-L **Additional file 2: Table S1**) were incorporated in our analysis of neuroanatomical phenotyping explained in **Additional file 1: Fig. S1**. The mutant mice were acquired through collaboration with various sources. Two third came from five mouse production centers of the International Mouse Phenotyping Consortium (IMPC): i) Mouse Clinical Institute (ICS), Illkirch, France (*Slx1b*, *Gdpd3*, *Ppp4c*, *Fam57b*, *Taok2*, *Kctd13*, *Sez6l2*, *Maz*, *Zg16* and *Spn*) ii) Wellcome Sanger Institute (WSI), Cambridge, UK (*Prrt2*), iii) Mouse Biology Program (MBP), UC Davis, USA (*Hirip3*), iv) Italian National Research Council (CNR), Monterotondo, Italy (*Ino80e*), and v) Texas A&M Institute for Genomic Medicine (TIGM) USA (*Mvp*). One third came from researchers worldwide who previously studied these mutant mice outside of the field of brain development including *Bola2*^1^, *Coro1a*^2^, *Mapk3*^3^, *Tbx6*^4^, *Doc2a*^5^, *Kctd13*^6^ and *Qprt*^7^. Each mouse transfer was approved by a Mouse Transfer Agreement with the corresponding laboratories. For four lines (*Bola2*, *Coro1a*, *Prrt2* and *Qprt*), for which animal transfer was not possible, brain samples were obtained instead by collaborators instructed to use our own standard operating procedures.

Twenty-one mouse mutants were generated using a gene targeting approach (exception *Mvp* used gene trapping, see below) noted by the superscript “tm” for targeted mutation or “em” for endonuclease mediated mutation (see column I in **Additional file 2: Table S1**). The targeted approach comprised three strategies: i) the “Knockout-first allele” strategy^8^ producing tm1a (*Fam57b*, *Ino80e*, *Sez6l2* and *Prrt2*) and tm1b (*Taok2* and *Sez6l2*) alleles (**Additional file 9: Fig. S13A**), ii) the reporter-tagged deletion strategy producing tm1 (*Bola2*, *Mapk3*, *Tbx6*, *Doc2a* and *Hirip3*) and tm1.1 (*Coro1a*, *Kctd13* and *Qprt*) alleles (**Additional file 9: Fig. S13B**), and iii) the CRISPR/Cas9 strategy producing em1 (*Slx1b*, *Gdpd3*, *Ppp4c*, *Maz*, *Zg16* and *Spn*) and em2 (*Kctd13*) alleles, similar to a previous report^9^.


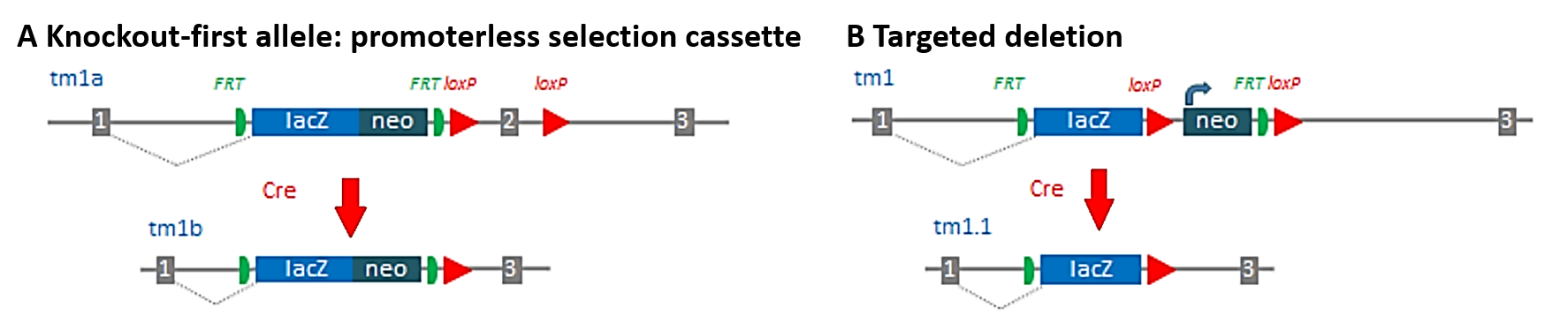


**Additional file 9: Fig S13.** Mutant allele design.

(**A**) ‘Knockout-first’ strategy. (**B**) Deletion allele with the promoter-driven targeting cassette. Green boxes represent FRT sites cleaved in the presence of Flipase (Flp). Red triangles represent loxP sites cleaved by Cre-recombinase (Cre). The insertion of the LacZ and neo cassette is expected to disrupt gene function. Adapted from IMPC.

The “Knockout-first allele” strategy relies on the identification of a critical exon (CE) common to all transcript variants, upstream of which a LacZ cassette was inserted to make a constitutive knockout named tm1a. Unlike the tm1a allele, tm1b creates a frame-shift mutation upon deletion of the selected exon after constitutive Cre-recombination. Alleles annotated tm1 were generated by homologous recombination targeted to the CE and replacing it by LacZ and promoter-driven Neo cassettes, flanked by FRT and loxP sites. Tm1 alleles can generate a tm1.1 derivative allele through Cre excision, which will remove the Neo cassette from the locus. Alleles that were generated by CRISPR/Cas9 also relied on a CE that was deleted using four gRNAs (two gRNAs 5’ and two gRNAs 3’ to the CE region). A single genotype-confirmed F1 mouse was produced to establish the colony used to generate mice for phenotyping. For *Kctd13* both tm1.1 and em2 strategies were used. Mvp mouse model was generated using a gene trapping strategy at the Texas A&M Institute for Genomic Medicine (TIGM). The clone number utilized was IST10954E2. The strategy relied on an insertion of a β-galactosidase/neomycin phosphotransferase (β-Geo) cassette within the first intron of the *Mvp* gene. The cassette was flanked by a splice acceptor and a polyadenylation site which triggers gene transcription premature arrest (**Additional file 1: Fig. S6A**). To ensure high comparability between mouse models, the mice used in this study came from the same genetic background strain C57BL/6 (see column J in **Additional file 2: Table S1**). We maintained most of the mice on a pure inbred C57BL/6N background (Taconic Biosciences) representing more than 80% of the lines tested in this study to minimize variation in phenotyping results due to strain differences. One gene (*Kctd13*) was tested on both C57BL/6N (B6N) and C57BL/6J (B6J) genetic background.

Among the 30 protein-coding genes of the mouse 16p11.2 syntenic region, ten were not available during the course of the study for various reasons (see column H in **Additional file 2: Table S1**). Two mutant lines (*Pagr1a* and *Kif22*) were at the stage of embryonic stem cell production therefore were not yet produced. Five (*Aldoa*, *4930451I11Rik*, *Tmem219*, *Cdipt* and *AI467606*) were cryopreserved at the Baylor College of Medicine (Texas USA) and one (*Ypel3*) at the Mouse Biology Program (MBP) UC Davis USA, but their rederivation was proven difficult. Two (*Sult1a* and *Asphd1*) were reassigned to a new partner of the IMPC: the Czech Centre for Phenogenomics (CCP) at the Institute of Molecular genetics of the Czech Academy of Sciences (IMG), Czech Republic, Europe.

### 1.2 Double knockout model

To study genetic interaction between *Mapk3* and *Mvp*, we generated a double-knockout line by crossing single-gene mutant models.

Because the two genes are on the same locus, the gametes resulting from meiosis of each can only segregate for one of the mutations, with 50% chance to segregate as a heterozygous or WT haplotype. Our breeding strategy gives rise to four groups, equally distributed with 25% chance to get each genotype in the F1 generation. For this study, we bred *Mapk3* mice with *Mvp* mice. The sex of the parent bearing each allele deletion was not taken into account. Considering the core strain of each of our mouse model, F1 generation from *Mvp* and *Mapk3* breeding was characterized by a mixed genetic background (50%B6Nx50%B6J).

Double-heterozygotes, WTs as well as the two intermediate heterozygotes for each single-gene knockouts were present in the offspring segregating according to Mendelian ratio. Four groups of mice were assessed when it was possible.


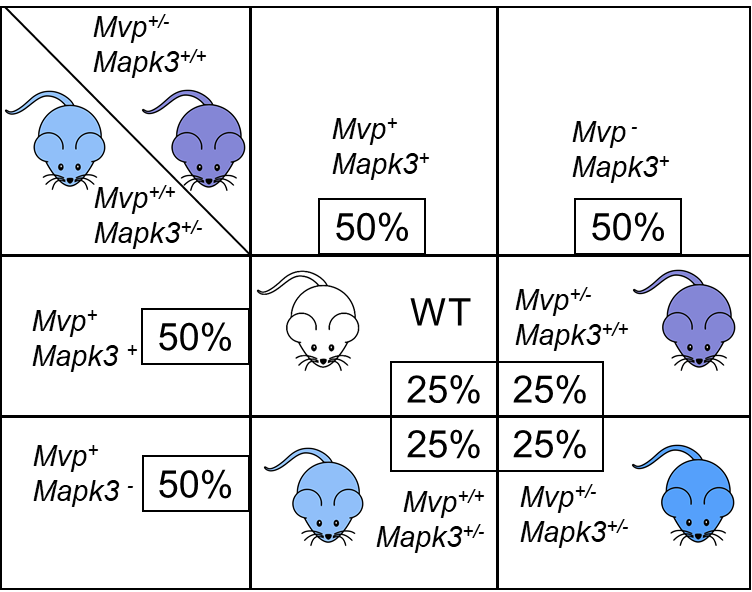


**Additional file 9: Fig S14.** Double knockout breeding strategy. Punnett square recapitulating the expected birth ratio for each genotype after the breeding of *Mvp* and *Mapk3* heterozygous mice. *Mvp^+/+^;Mapk3^+/+^, Mvp^+/-^;Mapk3^+/+^, Mvp^+/+^;Mapk3^+/-^* and *Mvp^+/-^;Mapk3^+/-^* are generated at equal ratio (25%).

### 1.3 Mouse model of the 16p11.2 locus

Del(7Sult1a1-Spn)6Yah mice deleted for the entire 16p11.2 locus (hereafter named as *Del/+*) were generated at the Mouse Clinical Institute, Illkirch, France (in the laboratory of Dr. Yann Hérault) and analyzed for NeuroAnatomical Phenotypes. The deletion encompassed the region from *Sult1a* to *Spn* and was previously described^10^. In this study, we decided to test the impact of the chromosomic rearrangement in the pure genetic background strain (C57BL6/N), to facilitate data integration with the single-gene knockout mice all processed on identical genetic background (C57BL/6).

## 2 Animal welfare

Seventeen of the 21 single-gene mouse lines were housed and maintained within our own animal facility at the Mouse Clinical Institute, Illkirch, France (see column L in **Additional file 2: Table S1**). A typical stocking density of 3-5 mice was used per cage in individually ventilated caging under a controlled 12-hours light/dark cycles. Animals were provided with chow food and water *ad libitum*. The ambient temperature was 21±2^o^C and the humidity was 55±10%. All animals were regularly monitored for health and welfare concerns and were additionally checked prior to and after procedures. In addition to bedding substrate, standard environmental enrichment of a couple of nestlets and a cardboard tunnel were provided. The care and use of mice in the study was carried out in accordance with the local ethics committee (Com’Eth) under the reference number 2016010717527861. The housing conditions described for single-gene models were the same than the ones for double-knockout models, as well as 16p11.2^Del/+^ mice.

## 3 Genotyping and sexing of study samples

Genotyping primers were designed for each of the seventeen mouse lines housed in our own animal facility (Mouse Clinical Institute, Illkirch, France). A list of sequences is provided in **Additional file 9: Table S13** along with respective annealing temperature and expected product size in both WT and KO conditions. Sexing was determined visually except for embryos and young postnatal age where we combined visual assessment with genetic testing using the SRY reactions.

**Additional file 9: Table S13.** Genotyping and sexing primers. List of the different primers used in this study to determine the genotype and sex of each mouse. Annealing temperature for PCR and expected product size are indicated.

Mice were genotyped according to the following instructions. Genomic DNA was extracted from mouse tail clip based on sample lysis by detergents and purified by precipitation using isopropanol. Polymerase chain reactions (PCR) were carried out with dNTPs (Roche), 10x PCR Buffer (Roche) and TaqPolymerase (Roche) in a MasterCycler (Eppendorf) under the following conditions: 95°C for 4minutes (1 cycle), 94°C for 30 seconds followed by another 30 seconds at primer-specific annealing temperature and 72°C for 1minute (34 cycles), 72°C for 7minutes (1 cycle) and then 4°C until use. PCR products were then loaded on 3% agarose gel with Ethidium Bromide (BET) (0.3μg/ml, Euromedex) and run for 25 minutes at 130V in an electrophoresis setup. The gel was then revealed using UV light.

The genotyping strategy to assess double-knockout lines consisted of a combination of the genotyping of corresponding single-gene models. DNA extracts were used with specific primers and genotyped separately. The combined genotypes were then merged in the same file to obtain final reading. The genotyping of the 16p11.2 mice was conducted as previously described^10^.

## 4 Brain histological screen

### 4.1 Sample processing

Brain samples were harvested from mice using a high-throughput approach, where each mouse was characterized by a series of standardized operating procedures^11^. The collection of brain samples was performed blind with experimenters not knowing the genotype of the mouse. Factors thought to affect the variables were standardized as far as possible. Where standardization was not possible, steps were taken to reduce potential bias. For example, as recommended in the ARRIVE guidelines^12^, we used a “minimized operator” defined as “The process by which steps are taken to minimize the potential differences in the effector by training and monitoring of operator”.

Brain samples from at least three mice per genotype and per gender were collected, which was estimated based on power calculation, limited throughput of both the necropsy collections and histological workflows and 3Rs in regards to animal use. Control brains were systematically collected within each of the mutant lines. Six cohorts representing a total of 458 mice were analyzed with the main difference being the number of genes targeted by the mutation. The first was for single-gene mutation, consisted of 347 mice made of 180 mutants and 167 matched WTs. The second dataset was for the analysis of homozygous *Kctd13* mice, composed of 14 individuals. The third dataset was for the analysis of *Mvp* homozygous mice, composed of 10 individuals. The fourth was for *Mvp* analysis at different time points, composed of 42 mice of 13 mutants and 29 matched WTs, The fifth was for *Mvp;Mapk3* double-gene mutations composed of 20 mice with 9 mutants and 11 matched WTs, and the sixth for deletion of the entire 16p11.2 interval on background C57BL/6N comprising 24 mice of 7 mutants and 17 matched WTs (we are not reporting the data on the C3B background).

For validation purposes, a set of mutants was studied multiple times through the production of independent cohorts or at different ages. Typically, adult mice aged 16 weeks old were studied (exception of *Ino80e* and *Ppp4c* aged 24-25 weeks old). *Kctd13^<tm1.1Nk>^* mice were studied at 8 and 16 weeks of age.

An overview of sample processing is shown in **Additional file 1: Fig. S1**. Standard operating procedures are described in more details elsewhere^11^. Animals were euthanized in a CO_2_ chamber, and brains were carefully collected and fixed in 10% neutral buffered formalin. The samples were transferred to 70% ethanol 48 hours after drop fixation. The cerebellar part was discarded and the remaining brain was trimmed at coronal plane around Bregma -0.10mm to produce two blocks and then embedded in paraffin using an automated embedding machine (Sakura Tissue-Tek VIP). Brains were cut at a thickness of 5μm on a sliding microtome (HM 450 Microm France) on symmetrical and stereotaxic planes such that we obtained sections matching well defined planes at Bregma +0.98 mm and Bregma -1.34 mm. Our precision was estimated to be no more than 30μm, anterior or posterior, to the histological section of interest. The sections were doubled-stained with 0.1% Luxol Fast Blue (Solvent Blue 38; Sigma-Aldrich) and 0.1% Cresyl violet acetate (Sigma-Aldrich), in order to label myelin and neurons, respectively. After mounting on slides, the sections were scanned at cell-level resolution using the Nanozoomer whole-slide scanner 2.0HT C9600 series (Hamamatsu Photonics, Shizuoka, Japan).

Every aspect of the procedure was managed through a relational database using the FileMaker (FM) Pro database management system (detailed elsewhere^13^), for example generating image scan names and directory architecture for 2TB of image data, image quality control, measurements and statistical pipelines using FM build-in scripting capabilities and an interface with R (version 3.4.0, <https://www.r-project.org/>) and ImageJ (Fiji, version 1.51e) scripts. Within this database, co-variate and measurement data for about 1000 brain images were collected and QCed entirely blind to the genotype.

### 4.2 Histomorphometric analyses and quality control

**Additional file 1: Fig. S1** gives the overall brain histomorphometric procedure. Careful and precise sectioning is a prerequisite in this protocol; sections must be symmetrical and match well-defined anatomical features (explained above). By contrast to more conventional histopathological screens that often rely on qualitative assessment, we used a quantitative approach where each section had to pass well-defined stereotaxic coordinates defined accordingly to the Mouse Brain Atlas before image analysis. Each image was quality controlled to assess whether (i) the section is at the correct position, (ii) the section is symmetrical, (iii) the staining is of good quality, and (iv) the image is good quality. Only images that fulfilled all of the quality control checks were processed. These quality control steps are essential for the detection of small to moderate neuroanatomical phenotypes (NAPs) and without which the large majority of NAPs would be missed. This is explained in great details elsewhere^11^.

A total of 35 co-variates, for example sample processing dates and usernames were collected at every step of the procedure (**Additional file 3: Table S7**), as well as 67 brain morphological parameters of 39 area and 28 length measurements (**Additional file 2: Table S2**). Out of the 67 coronal brain parameters analyzed, 50 were measured on both the left and the right hemispheres including 20 in the cortices and 8 in the hippocampus. Since our histomorphological procedure does not allow interhemispheric comparisons for sure (sometimes sections can turn upside down during the histological procedure), we combined measurements of left and right hemispheres, reducing the number of coronal parameters to 42.

These parameters encompass five main categories: brain size, commissures (callosal, anterior and hippocampal), ventricles (lateral and third), cortex (motor, somatosensory, cingulate, piriform and retrosplenial) and subcortex (hippocampus, amygdala, tracts, caudate putamen, internal capsule, habenula, fimbria and hypothalamus). The total number of neuroanatomical measures taken throughout the study was 19,278. All samples were also systematically assessed for cellular ectopia (misplaced neurons). Of note, amongst all the mouse mutant lines assessed in the study, we found no occurrence of ectopia.

### 4.3 Statistics

We began by a quality control step of the collected data and checked for the presence of erroneous measures, typographical errors and outliers, and corrected the measurement if necessary. At this step, we removed from the study female *Qprt^+/-^* data, since the data quality checks for this group did not fulfil our expertise criteria. To assess whether assumptions for statistical tests were met, quantile plots were drawn for each of the brain parameters in matched WTs where normality was met for most parameters at the exception of the size of the ventricles. Data were analyzed using a two-tailed Student t-test of equal variance to determine whether a brain region is associated with a neuroanatomical phenotype or not. We critically evaluated any results with p-values lower than 0.05. The mutant samples were analyzed using their colony controls (the same mouse line). The percentage change of affected structures relative to the WTs (set to 100%) was also calculated as following: %change= (average mutant/average WT)*100*.*

**Additional file 4: Table S8** provides association and percentage change data for the twenty-one assessed single-gene alleles across 42 left and right combined parameters. In order to determine the level of confidence of each of the NAP, they were ranked within six different categories (NeuroAnatomical Threshold (NAT), defined after their p-value (below 0.05, 0.01, 0.005, 0.001, 0.0005 or 0.0001). The definition of a NAP is only possible according to a specific NAT.

## 5 Follow-up histology studies of MVP/vault

### 5.1 Embryonic stage

For embryos at E18.5, the whole embryo was fixed in Bouin solution for 48 hours. The embryonic brains were then harvested, transferred to 70% ethanol, and manually embedded in paraffin using the following steps: three incubation baths in 70% ethanol for 30 minutes each, two baths in 95% ethanol for 30 minutes each, two baths in 100% ethanol for 45 minutes each, three baths in Histosol Plus for 1 hour each, and five baths in warm paraffin (60°C) for 30 minutes each, followed by incubation in warm paraffin overnight before casting in a mold. Brains were cut at a thickness of 5μm on a microtome (HM 450, Microm Microtech, France), such that we obtain sections matching planes displayed in **Additional file 9: Fig. S15** for embryonic coronal sections. The sections were stained with 0.1% Cresyl violet acetate (Sigma-Aldrich) and scanned using Nanozommer 2.0HT, C9600 series at 20× resolution.


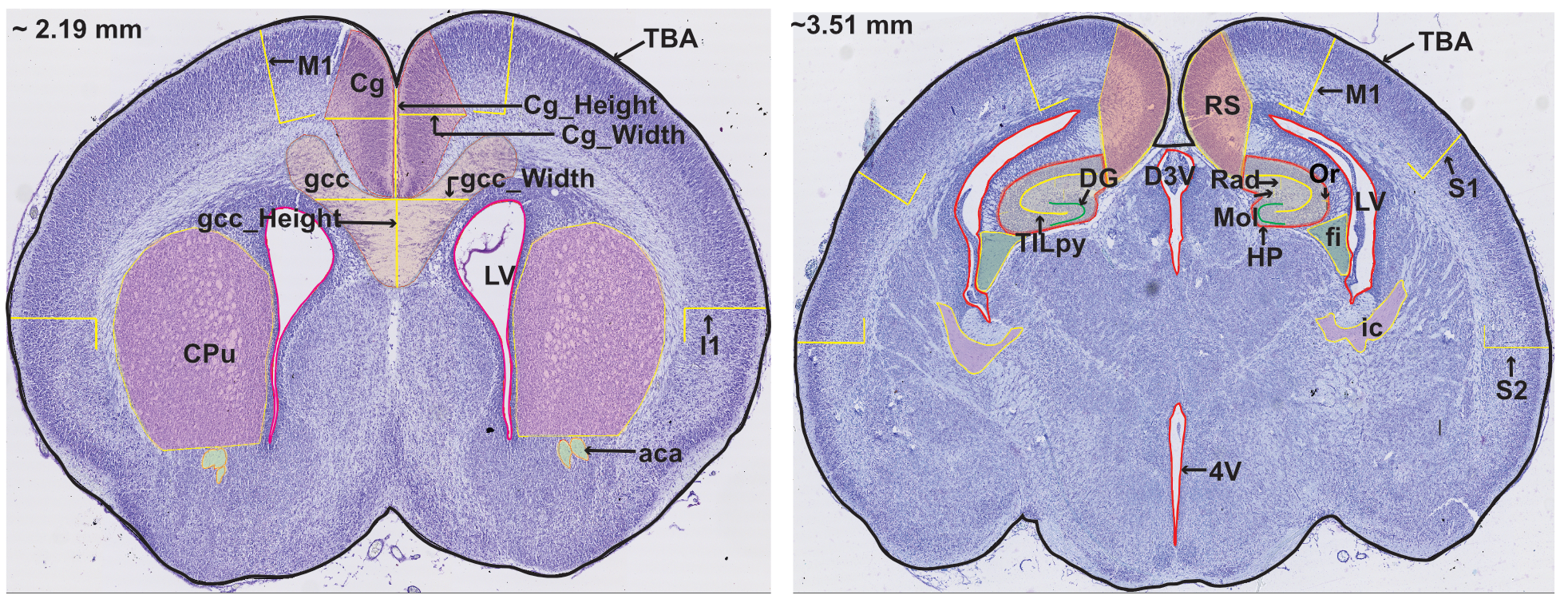


**Additional file 9: Fig S15.** Coronal section of interest at E18.5.

Measured regions are delineated and associated with their parameter names on representative images of the two coronal sections of interest. See **Additional file 2: Table S5** for the description of acronym used.

### 5.2 Sagittal orientation

After dissection and fixation, the brains were cut into halves along the sagittal midline, and embedded separately in paraffin. The section of interest was determined as corresponding to Figure 106 of the Mouse Brain Atlas (Lateral +0.60 mm). Brains were sectioned to match the defined section at a thickness of 5μm. The staining, scanning, and quality control steps were identical to the coronal procedure. 40 brain morphological parameters, made of 25 areas, 14 lengths and 1 number, were measured for each brain. This is described in more details in **Additional file 2: Table S6**.

### 5.3 Determination of layers in somatosensory and motor cortices

Cortical layers are easily identifiable on histological sections. One of our question was to determine if the neuroanatomical defaults observed in male cortices were restricted to specific layers or if the whole cortex was impacted. To do this, we set the line used to measure the length of these cortices (1_S2 and 1_M1 in red in the **Additional file 9: Fig. S16**), as the base to create a rectangle, with 0.22 mm (green) or 0.65 mm (yellow) wideness, respectively, which allows the measurement of the area of these cortices.


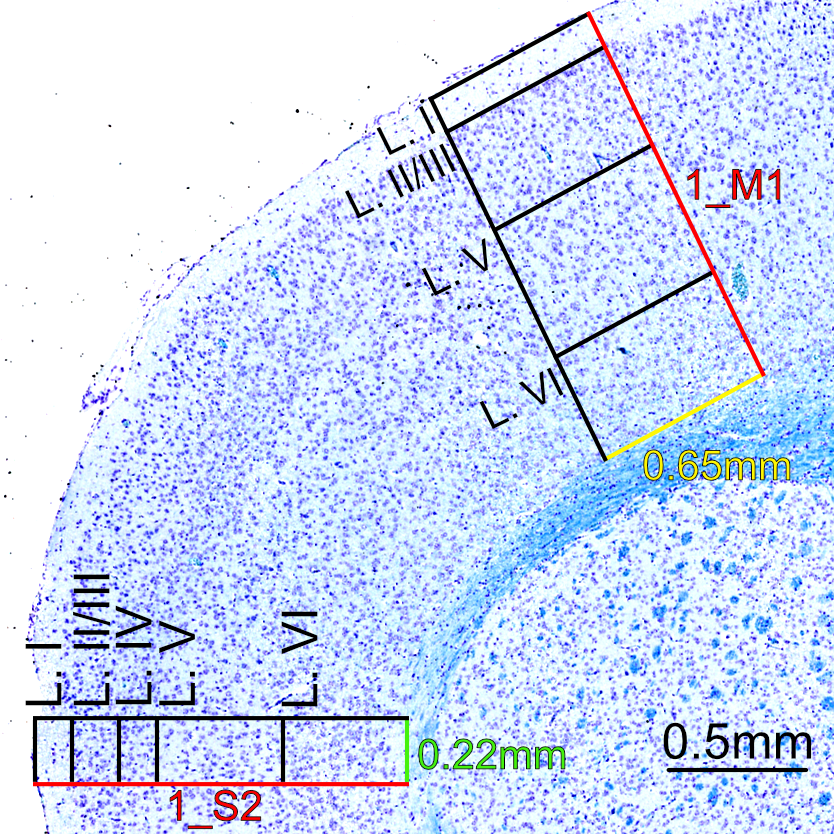
Then, we manually delineated each of the layers according to their cytoarchitecture features: Layer I: external layer with very few cells; Layer II/III: densely packed neurons; Layer IV (not present in motor cortex): more densely packed neurons; Layer V: loosely packed with bigger neurons; Layer VI: medial, densely packed neurons. The area as well as the cell count and averaged cellular area were measured and calculated in these regions.

**Additional file 9: Fig S16.** Cortical layer determination.

### 5.4 Measurement of cellular features

We developed an automated plugin in Fiji to measure the number of cells as well as the averaged cell area of each cell. These measurements are done on areas already delineated when measuring the area of the structure of interest. For example, cingulate gyrus cell count (1_Cg_cellcount) and cell area (1_Cg_avgcellarea) are measured in the area determined as being the cingulate gyrus (1_Cg) in the automated protocol. The total area of all cells within the region (cellarea) is divided by the number of cells in order to determine the averaged area of each cell (avgcellarea): avgcellarea = cellarea/cellcount.

## 6 Evaluation of MVP/vault transcript level

### 6.1 Tissue dissection

Whole brain from WT and *Mvp* mutant mice were harvested at different developmental (E16.5, E18.5, P0, P10, P30) and adulthood (10, 16 and 30 weeks old) stages. Until P10, mice were decapitated with sharpened scissors and P30 and adult cohorts by increased CO_2_ inhalation in gas chamber. For some adult cohorts, brains underwent microdissection in order to separate various brain structures of interest (such as cortex (CTX), hippocampus (HP), striatum (STR) and cerebellum (CRB)). Anterior cingulate cortex and primary somatosensory cortex were very carefully harvested using a mouse brain matrix. Additional peripheral tissues such as liver, lung, kidney and heart were also extracted from the same mice. Tissue samples were stored in cryotubes (Nunc) and deeply freezed in liquid nitrogen to prevent RNA degradation. Excess of tissue samples were kept at -80°C for long-term storage.

### 6.2 RNA extraction

Total RNA was extracted from samples by using a phenol-chloroform technique. Samples were transferred and homogenized with TRI Reagent (T9424, Sigma Aldrich) in tubes containing ceramic beads (Precellys Lysing Kit). The phase separation was done by addition of chloroform followed by centrifugation at 12000g for 5 minutes. Supernatant was collected and RNA extraction was conducted with RNeasy Plus Mini Kit (74134, Qiagen) as told by the manufacturer. RNA concentration and quality were assessed by spectrophotometry (Nanodrop 2000, Thermo Scientific). Only RNA samples with a ratio A_260/_A_280_ close to 1.8 and a ratio A_260/_A_230_ above 2.0, to exclude guanidine thiocyanate-containing buffer contamination, were further processed. RNA integrity number (RIN) was assessed using the Bioanalyser (Agilent 2100). Samples with RIN < 8 were not used in downstream experimental settings.

1µg of total RNA was reverse-transcribed to complementary DNA (cDNA) using SupercriptIII First-Strand Synthesis Supermix (11752–050, Invitrogen) for qualitative and quantitative reverse transcription PCR (RT-PCR). cDNA was stored at -20°C for future usage.

### 6.3 Qualitative RT-PCR

RT-PCR reactions were utilized in order to visually assess transcript expression level. This was done in four independent experiments: i) compare differences in *Mvp* expression between brain structures in WT, ii) compare *Mvp* expression patterns between male and female WT mice, iii) validate the *Mvp* mutant mouse model, iv) assess the expression level of the overlapping gene *Pagr1* in the *Mvp* mutant mouse model. PCR reactions were carried as described in section 4.3 of the present document. Primers were specially designed in order to amplify a region spanning two adjacent exons in order not to amplify genomic DNA (**Additional file 9: Table S14**). PCR products were then loaded on 3% agarose gel containing BET and migrated by electrophoresis at 130V.

**Additional file 9: Table S14.** Sequences of RT-PCR primers.

### 6.4 Quantitative RT-PCR (qRT-PCR)

**
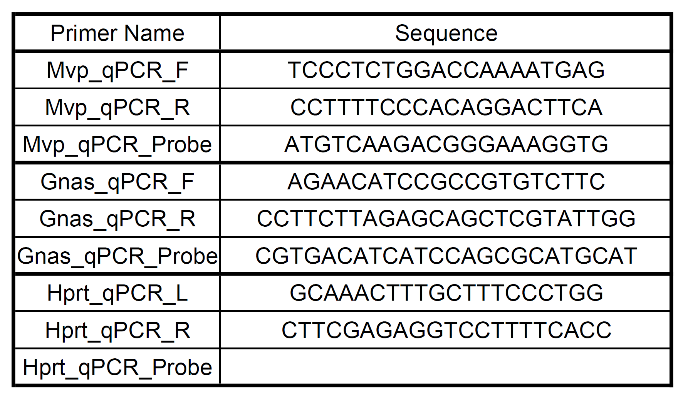
**Efficiencies of the TaqMan assays were checked using a cDNA dilution series from the extracts of cortex (CTX), hippocampus (HPC), cerebellum (CRB) and liver (LIV). PCR reactions were performed using Fast Blue qPCR Mastermix Plus (RT-QP2X-03+FB; Eurogentec) with 300nM of each primer and 100nM of FAM-labelled TaqMan probes in a final reaction of 15μl with a standard amplification procedure (T°A=60°C, 40 cycles). Normalization was performed by carrying out in parallel the amplification of two other housekeeping genes (*Hprt* and *Gnas*). All the tested samples were performed in triplicate (male and female mice were aged 12 weeks), and the results were reported as the mean ± SEM.

**Additional file 9: Table S15.** qRT-PCR primers and probes

## 7 Immunohistofluorescence

### **7.1 Sample processing**

C57BL/6N adult mice of 12 weeks of age (3 males and 1 female) were anesthetized using Ketamine (150mg/kg) and Xylazine (400ug/kg) in NaCl, and intracardially perfused with PBS, to remove blood within the vessels, and then fixed with 4% Paraformaldehyde (PFA) solution in PBS. Brains were harvested, post-fixed in 4% PFA for 24 hours at room temperature (RT), and then transferred in 30% sucrose solution for 24 hours at 4°C to ensure cryoprotection of the tissues. Each brain was then cut into either three (for coronal sectioning) or two (for sagittal sectioning) and embedded in Cryomatrix (Thermo Scientific) with a fast freeze device PrestoChill (Millestone).

### **7.2 MVP/Vault immunostaining**

Brains were sectioned throughout the entire tissue blocks with a cryo-microtome (K400 station with dry ice on HM 450 microtome, Microm Microtech France) at 30µm in thickness. Floating sections were then stored at 4°C in 0.1% sodium aside in PBS.

Immunostainings for MVP were realized without detergent. The permeabilisation step was done by incubation of the brain histological sections in methanol at -20°C for 10 minutes. Sections were rinsed in PBS, blocked in a solution with 10% Normal Donkey Serum (NDS) and 1% Bovine Serum Albumin (BSA) in PBS and incubated with rabbit polyclonal vault antibodies (N2-B15, provided by Leonard Rome, dilution 1:1000) and mouse anti-NeuN antibodies (MAB377, Millipore, dilution 1:1000) in 0.1%NDS/PBS at 4 °C overnight under agitation. After washing with PBS, the sections were incubated for three hours RT with fluorescence-conjugated secondary antibodies coupled to anti-rabbit-Alexa-488 and anti-mouse-Alexa-647 (1:1000, Thermo Fischer Scientific). Nuclei were counterstained with Hoechst 33342 (1:10,000 dilution; Sigma-Aldrich), and sections were mounted on Superfrost blades using FluorSave mounting solution (Calbiochem). The slides were stored in the dark at 4 °C. Images were acquired using confocal microscope (TCS SP5; Leica) at 20× or 80x magnification and analyzed using ImageJ software. We carefully assessed MVP/vault expression as mild (+), moderate (++) or strong (+++) across all the positive brain regions (**Additional file 2: Table S4**).

### 7.3 Phospho-ERK immunohistofluorescence

ERK activity was studied in *Mvp^+/-^;Mapk3^+/-^* double-heterozygous, their matching singe-heterozygous ( *Mvp^+/+^;Mapk3^+/-^* and *Mvp^+/-^;Mapk3^+/+^*) and WT mice in both male and female. The brains were sectioned (10μm), then rehydrated in PBS for 10 min. Antigen retrieval using 1× Reveal Decloaker (Biocare) was performed for 10 min at 95°C. Sections were blocked in 2% (postnatal) normal goat or donkey serum for 1 h at room temperature with 0.1% (v/v) Triton X-100 in PBS. Slides were incubated with primary antibodies (polyclonal rabbit anti-pERK (Cell Signaling Technology; 1:100)) overnight at 4°C, rinsed with PBS and incubated with corresponding secondary antibodies (AlexaFluor 488 conjugated to goat anti-rabbit (Invitrogen) (1:1000)) for 1–2 h at room temperature. DNA was stained with 4′,6′-diamidino-2-phenylindole for 5 min (Invitrogen).

We counted at least three consecutive tissue slices per animal. In postnatal brains, coronal sections were used to count cells in 400μm boxes in prefrontal cortex. Slides were picked at random and the investigator was blinded to genotypes. The number of mice evaluated is indicated in figure legends, with three to seven mouse brains per group.

## 8 Western Blot Analysis

Livers from adult male mice were dissected and homogenized with 300μL of lysis buffer containing 1× RIPA buffer (ThermoFischer), phenylmethylsulfonyl fluoride 1%, sodium orthovanadate 1%, and protease inhibitor 1% in tubes containing ceramic beads (Precellys Lysing Kit). The tubes were incubated for 30 minutes at 4°C and centrifuged for 20 minutes at 17,000 × g at 4 °C, and the supernatant was isolated for Western blotting. 60μg of protein was separated on 10% SDS/PAGE (Mini-PROTEAN TGX Gels, 12%, 10-well, 4561043, BIO-RAD) and transferred onto nitrocellulose membrane (#1620115, BIO-RAD). Membranes were blocked with 1% BSA diluted in tris buffered saline with Tween 20 (50mM Tris, 150mM NaCl, 0.05% Tween 20) and probed using either rabbit MVP-antibodies (named “George”, provided by Leonard Rome), ERK-antibody (4370S, CellSignalling) or Phospho-ERK (4695S, CellSignallig) overnight at 4°C. Membranes were then incubated with horseradish peroxidase (HRP)-conjugated anti-rabbit (GE Healthcare) secondary antibody. Antibody–protein interactions were revealed using chemiluminesence (RPN2108; GE Healthcare), and relative protein expression was quantified using ImageJ (<https://imagej.nih.gov/ij>).

## 9 Immunocytofluorescence

### 9.1 Primary neuronal cultures

Female mice on gestation were culled in increasing CO_2_ gas chamber, and embryos harvested at embryonic day 18.5 (E18.5) then placed in dissection solution S1 (Phosphate Buffer Saline (PBS) 1x, Bovine Serum Albumin 3mg/ml, D-glucose 3mg/ml, MgSO4 1mM). The skin and skull of each embryo were removed and the two brain hemispheres separated. The hippocampus and the cortex were harvested from each hemisphere and collected in their corresponding tubes with 500μl of S1 solution. The samples were then centrifuged at 10,000g for 1 minute, and the supernatant removed. 500μl of enzymatic dissociation solution S2 (S1 + Trypsin 2.5mg/ml and DNase I 2.5mg/ml) was then added to each sample and incubated for 20 minutes at 37°C by intermittently mixing the tubes. 500μl of Trypsin inhibition solution S3 (S1+ Soybean Trypsin Inhibitor (SBTI) 80μg/ml, DNase 130μg/ml and MgSO_4_ 250μM) was added to each sample in order to stop dissociation and centrifuged at 10,000g for 5 minutes. The supernatant was removed without disturbing the pellet, and 300μl of mechanical dissociation solution S4 (S1+ SBTI 0.5mg/ml, DNase 0.08mg/ml and MgSO_4_ 1.5mM) was added. The cells were then dissociated by pipetting up and down and centrifuged at 10,000g for five minutes. The supernatant was then replaced by seeding medium (DMEM, HEPES 10mM, Horse serum 10%, Gentamycin 50μg/ml and Insulin 1μg/ml), and cells were counted using the Hemocytometer (Malassez cell). Between 20,000 and 30,000 cells were plated on Poly-L lysine coated coverslips in 24-well plates and incubated at 37°C with 5% CO_2_. After 24 hours, the medium was replaced using filtered GIBCO Neurobasal Medium1x (Invitrogen) supplemented with GIBCO B27 medium 1x (Invitrogen), Penicillin, Streptomycin and L-Glutamine (0.5mM). After 4 days of *in vitro* culture (DIV4), the cells were fixed with 4% PFA in 6% sucrose for 15 minutes, and then stored in EtOH at 4°C until use.

### 9.2 Immunocytostaining

Fixed cells were incubated O/N at 4°C with primary antibodies (rabbit polyclonal anti-MAP2 (AB5622, Millipore) and mouse monoclonal antibody against pan-axonal neurofilaments (SMI-312R, Covance) both diluted at 1:1000 in saturation solution (0.2% Triton, 1% BSA, 10% Normal Donkey Serum (S2170, Dutscher) in Tris-Buffered Saline). Donkey anti-rabbit coupled to Alexa647 (ab150075, Abcam) and donkey anti-mouse coupled to Alexa488 (ab150105, Abcam) were used as secondary antibodies in saturation solution without Triton. Nuclei were stained with Hoechst 3342 (1:10,000 dilution; Sigma-Aldrich). The coverslips were then mounted on slides by using AquaPolyMount (PolyScience, Inc.) and stored at 4°C until imaging.

### 9.3 Cell morphological assessment

Images were acquired using a regular epifluorescence microscope 100x (Leica) at magnification of 0.55x and analyzed using ImageJ (https://imagej.nih.gov/ij). The area of the soma was determined following the MAP2 staining around the cytoplasm, often characterized by an ovoid-like shape. The processes and lamellipodia were entirely excluded in the measurement. Axonal length was analyzed by measuring the length from the start of the axon until the tip of the primary axonal process, stopping when the growth cone begins. The growth cone area was defined and measured from the beginning of the terminal widening of the axon and included all visible lameli- and filopodia (see **Fig. 4F** for schematic explanation). Shapiro-Wilk normality test revealed that these datasets followed a lognormal distribution. We then converted all of our data into log(10) to perform the statistical analysis using a two-tailed Student’s *t* test. 0.05 was set as the significance threshold. Graphs represent the mean + SEM of the raw data.

## 10 RNA sequencing

RNA-Seq libraries were generated from 200 ng of total RNA using TruSeq Stranded mRNA LT Sample Preparation Kit (Illumina, San Diego, CA), according to manufacturer's instructions. Briefly, following purification with poly-T oligo attached magnetic beads, the mRNA was fragmented using divalent cations at 94°C for 2 minutes. The cleaved RNA fragments were copied into first strand cDNA using reverse transcriptase and random primers. Strand specificity was achieved by replacing dTTP with dUTP during second strand cDNA synthesis using DNA Polymerase I and RNase H. Following addition of a single 'A' base and subsequent ligation of the adapter on double stranded cDNA fragments, the products were purified and enriched with PCR (30 seconds at 98°C; [10 seconds at 98°C, 30 seconds at 60°C, 30 seconds at 72°C] x 12 cycles; 5 minutes at 72°C) to create the cDNA library. Surplus PCR primers were further removed by purification using AMPure XP beads (Beckman-Coulter, Villepinte, France) and the final cDNA libraries were checked for quality and quantified using capillary electrophoresis. These libraries were sequenced on the Illumina Hiseq 4000 as Single-end 50 base reads following Illumina’s instructions.

Image analysis and base calling were performed using RTA 2.7.3 and bcl2fastq 2.17.1.14. Reads were preprocessed using cutadapt 1.10^14^ in order to remove adapter, polyA and low-quality sequences (Phred quality score below 20), reads shorter than 40 bases were discarded for further analysis. Reads mapping to rRNA and spike-in sequences were also discarded (this mapping was performed using bowtie 2.2.8^15^). Reads were then mapped onto the mm10 assembly of *Mus musculus* genome using STAR^16^ version 2.5.3a (--twopassMode Basic). Gene expression was quantified using htseq-count 0.6.1p1^17^ and gene annotations from Ensembl release 91. Statistical analysis was performed using R 3.3.2 and DESeq2 1.16.1 Bioconductor library^18^.

11 Golgi staining

GolgiCox staining was performed using the FD Rapid GolgiStain Kit (FD NeuroTechnologies, Ellicott City, MD) on entire fresh brains, right after being removed from the skull (4 WT, 6 *Mvp*^+/-^, 4 *Mvp*^-/-^), and processed as indicated by the manufacturer. After impregnation, brains were embedded in 3% low-melting agarose and cut with vibratome. 100µm thick sections were mounted on gelatine-coated slides and allow drying for two days before staining (following notice). Images were acquired with slide scanner Hamamatsu at 40x resolution with multi-layer mode (41 focal plan every 0.2µm), thus allowing the analysis of the dendrites over 8µm in the tissue. Typical pyramidal neurons from somatosensory cortex were identified, and 25-100 µm-long secondary apical dendrites were analyzed. The length of the dendrite section as well as the number of visible tangential dendritic spines were recorded to calculate the spine density (spine density= # of dendritic spine counted/length of the dendritic section measured). Data were collected and are presented in **Additional file 5: Table S9**. All the measurements were taken by the same operator, blind to the genotype and perform manually on NDPviewer2.0.

## 12 Electrophysiology

### 12.1 Preparation of acute slices

A total of 14 male mice (8 *Mvp^+/+^* and 6 *Mvp^-/-^)*, 16-19 weeks old, were anesthetized (urethane i.p., 1.9 g/Kg) and killed by decapitation. The brain was removed and immediately immersed in cold (0-4°C) sucrose-based artificial cerebrospinal fluid containing (in mM): 248 sucrose, 11 glucose, 26 NaHCO3, 2 KCl, 1.25 KH2PO4, 2 CaCl2 and 1.3 MgSO4 (bubbled with 95% O2 and 5% CO2). Transverse slices (400μm thick) were performed with a vibratome (VT1000S, Leica, Nussloch, Germany). Slices were maintained at room temperature in a chamber filled with artificial cerebrospinal fluid containing (in mM): 126 NaCl, 26 NaHCO3, 2.5 KCl, 1.25 NaH2PO4, 2 CaCl2, 2 MgCl2 and 10 glucose (bubbled with 95% O2 and 5% CO2; pH 7.3; 310 mOsm measured).

### 12.2 Electrophysiological recordings

Slices were transferred to a recording chamber and continuously superfused with oxygenated artificial cerebrospinal fluid. Pyramidal cingulate neurons were recorded in the whole-cell configuration. Patch pipettes were pulled from borosilicate glass capillaries (Harvard Apparatus, Edenbridge, UK) using a P-2000 puller (Sutter Instruments, Novato, CA, USA). They were filled with a solution containing the following (in mM): 145 KCl, 10 HEPES and 2 MgCl2 (pH 7.3, adjusted with KOH; osmolarity 310 mOsm adjusted with sucrose) (3.5–4.5 MΩ). All recordings were performed in presence of bicuculline (10μM) and tetrodotoxine (0.5µM. Voltage-clamp recordings were performed with a Multiclamp 700A amplifier (Molecular Devices, Union City, CA, USA) at a holding potential fixed at -60 mV. Recordings were acquired and analyzed with WinWCP 4.3.5 (courtesy of Dr. J. Dempster, University of Strathclyde, Glasgow, United Kingdom). All recordings were performed at 34°C.

## 13 Electronic microscopy

Adult (16 weeks) *Mvp^-/-^* male mice and corresponding WT were deeply anesthetized with Ketamine (150mg/kg) and Xylazine (400ug/kg) in NaCl, and intracardially perfused with PBS, and then fixed with 2.5% glutaraldehyde and 2.5% PFA in cacodylate buffer (0.1 M [pH 7.4]). Portion of cingulate gyrus were then washed 30 min in cacodylate buffer, post-fixed with 1% osmium tetroxide in 0.1 M cacodylate buffer for 1 h at 4°C, and dehydrated through graded alcohol (50%, 70%, 90%, and 100%) and propylene oxide for 30 min each. Samples were oriented and embedded in Epon 812. Semithin sections were cut at 2μm with the Leica Ultracut UCT ultramicrotome and stained with 1% toluidine blue and 1% sodium borate. Ultrathin sections were cut at 70nm and contrasted with uranyl acetate and lead citrate. Electron microscopy observation and image acquisition were performed at 70kv with the Morgagni 268D electron microscope (FEI Electron Optics, Eindhoven, the Netherlands) and equipped with the Mega View III camera (Soft Imaging System).

## 14 Behavioral Analysis

Behavioral experiments were conducted at the Mouse Clinical Institute (Illkirch, France), by the same experimenter, blinded to the genotype to avoid any potential biases. All mice tested were given several days of rest between experiments and were age matched. Four cohorts of male mice were analyzed: A) 12 mice *Mvp^+/-^*;*Mapk3^+/-^* *versus* 12 matched WT for the double heterozygous knockout mice of *Mvp* and *Mapk3*, B) 12 mice *Mvp^+/-^* *versus* 10 matched WT for the single-gene mutation of *Mvp*, C) 12 mice *Mapk3^+/-^ versus* 12 matched WT for the first cohort of the single-gene mutation of *Mapk3* and D) 12 mice *Mapk3^+/-^ versus* 12 matched WT for the second cohort.

We used a comprehensive pipeline of sixteen behavioral tests, including: (i) open field, (ii) elevated plus maze, (iii) fear conditioning, (iv) forced swim, (v) tail suspension test, (vi) pentylenetetrazol (i.v.PTZ) seizure test, (vii) grip strength test, (viii) rotarod coordination test, (ix) sucrose preference, (x) Y maze, (xi) novel object recognition 3 h, (xii) social recognition, (xiii) social interaction, (xiv) marble burying, (xv) circadian activity and (xvi) pre-pulse inhibition (PPI) test.

### 14.1 Behavioral pipeline

Behavioral analyses were done between 9 and 26 weeks of age to model phenotypes found in young adults with the 16p11.2 deletion. Only male mice were utilized for behavioral testing to avoid any estrous cycle interference. **Additional file 9: Table S16** gives the details of tests and the age at which they were carried out.

**Additional file 9: Table S16**. Behavioral pipeline.

Summary of the sixteen behavioral tests realized on four different cohorts (*Mvp*;*Mapk3*, *Mvp* and two distinct *Mapk3*). Each test is associated with a core function. The acronym used throughout this study is indicated in the third column. The age range of the mice for each test is referred in weeks.

### 14.2 Open field

The open-field test allows studying basic locomotor activity, hyperactivity, exploratory behavior and anxiety in mice. The mouse was put in an arena of dimensions 44.3 × 44.3 × 16.8 cm made of PVC (Panlab) fitted with two frames of 32 infrared (IR) beams and illuminated at 150 Lux, and their activity was recorded for 30 minutes using a video tracking system (Ethovision; Noldus); data were analyzed using Acti-Track software. Speed and distance covered by the mice were then quantified as well as the percentage of time spent in defined zones of the field (periphery, intermediate, or center) and the latency before entering the center for the first time. The natural avoidance of mice for highly luminous places was used as a proxy of anxiety level.

### 14.3 Elevated plus maze

We used the elevated plus maze (Imetronic) as an additional test to evaluate anxiety. The setup consisted of a plus-shaped arena illuminated at 50 Lux at an elevation of 66cm, with opposite arms being either open (without walls, 30 × 5 cm) or closed (with walls 30 × 5 × 15 cm). The apparatus was equipped with IR captors, allowing the detection of mouse movements in the enclosed arms and different areas of the opens arms. It was also possible to evaluate head dips over the open arm. The mouse was placed in the center and allowed to freely explore the arms for a period of 5 minutes. The index of anxiety was calculated against the time spent in the open arms as opposed to the total time spent exploring arms.

### 14.4 Fear conditioning

Learning and memory of aversive stimuli is a key function to assure survival for mice. To test for this, fear conditioning was used as a three-phase test: conditioning, contextual testing and cued testing. This was measured in a operant chamber (Coulbourn, Bilaney, Dusseldorf, Germany, www.blaney.com) to associate light/tone stimuli with a mild electrical foot shock. Freezing was measured as a proxy of learning/memory performance during each phase by using infrared sensor. During the conditioning phase, the mouse was placed in the chamber for six minutes. There were four minutes of habituation (Hab-1 and Hab-2, two minutes each) where freezing time was measured. This was followed by tone/light stimuli (Cue: blue light and 80 dB/10kHz tone) for 20 seconds and a mild electrical foot shock (Unconditional stimulus=US) of 0.4 mA for one second. Freezing time was recorded for two additional minutes (Post-US). On the following day, the mouse was put back in the operant chamber in which it had been trained to associate a tone/light with an electrical foot shock. Freezing to the background context (Cont) was measured over a period of six minutes (Cont-1, Cont-2 and Cont-3, two minutes each). Five hours later, the mouse was placed in a new chamber of distinct appearance (*i.e.* different wall color, floor texture odor) and amount of time freezing was measured for eight minutes. At the end of the first two minutes (Pre-cue1), the tone/light stimuli were presented for a period of two minutes (Cue1) and freezing measured again. This sequence was repeated once again (Pre-Cue2 and Cue2).

### 14.5 Forced swim

The forced swim test (also known as the despair test) was used to assess depression-like behaviors. The mouse was placed in a glass becher filled with water (20-21°C) with no possible escape and let for six minutes. The duration of immobility as well as the latency before the first immobilization were recorded.

### 14.6 Tail suspension test

The tail suspension test is another way to evaluate depression-like behaviors during which the animal cannot escape. The mouse was suspended above the ground by their tail with adhesive and observed for 6 minutes. The duration (in seconds) of immobility as well as the latency before the first immobility were evaluated.

### 14.7 Pentylenetetrazol seizure test

Pentylenetetrazol (PTZ) is a convulsing drug used at high dose to model status epilepticus in animal models. After intraperitoneal injection of PTZ (50mg/kg in NaCl 0.9%), the mouse is susceptible to enter clonic (shaking of the body due to skeletal muscles contractions) and/or tonic phase (elongation of the limbs). The duration of each phase and the latency before entering the seizure were manually recorded.

### 14.8 Grip strength test

Neuromuscular functions were assessed by recording the maximal force (in grams) that a mouse can exert while grasping at a metal grid attached to a dynamometer (Bioseb). The mouse was placed on the testing apparatus to grasp the grid and gently pulled back by their tail until the release. Resulting grip strength force was recorded and normalized as percentage of bodyweight. This test was used to evaluate forelimb muscular strength as well as a combination of the four paws and repeated for four successive trials.

### 14.9 Rotarod

The rotarod test was used to assess sensorimotor coordination. The mouse was placed facing the direction of rotation in a rotating bar of 5cm diameter (Bioseb) at several inches from the table. The mouse underwent three days of training during which the mouse was trained to stay on the rod for two minutes with increasing speed (from 4 to 40rpm) for maximum five minutes. The test was stopped when the mouse felt down. Each mouse underwent four trials every day and the latency before falling off the rotating bar was recorded. On the test day, the mouse was placed on the rod at determined speed (4, 10, 16, 22, 28, 34 or 40 rpm) and let for maximal two minutes (120 seconds). The test was stopped when the mouse felt down and the latency before falling off the rotating bar recorded.

### 14.10 Sucrose preference

Mice have a natural preference for sweets, which is associated with pleasure-seeking behavior. The mouse was exposed to two different drinking bottles, one with water and one with 0.8% sucrose, and recorded for its drinking consumption. In the habituation phase, the bottles were exposed for one hour for training purposes. On the following day, the bottles were left for 15 hours (from 6am to 9pm), and the weight of the bottles were measured. Sucrose preference was based on the percentage of sucrose beverage consumption over the total consumption (sucrose + water). Sucrose preference superior to 50% was considered as normal pleasure-seeking behavior and below 50% as anhedonia, associated with depression-like behavior.

### 14.11 Y maze

The Y-maze test was used to evaluate short-term working memory based on the innate curiosity of the mice to explore an arm that has not been previously explored; this preferential behavior, when occurring at a frequency greater than 50%, is called spontaneous alternation. The testing apparatus consisted of a Y-shaped maze with three white, opaque, Plexiglas arms of equal length (40 × 9 × 16 cm) at 120° angles to each other, each identified with walls designed with unique motifs, illuminated at 100 Lux. Animals were placed at the center of the maze and allowed to explore freely the three arms for 8 minutes. The numbers of arm entries were recorded and quantified to yield percentage of alternation.

### 14.12 Novel object recognition

The novel object recognition is similar to the Y-maze test, as it is based on the innate tendency of mice to explore novel objects over familiar ones. The animals were habituated (100-Lux illumination) and tested (70-Lux illumination) in the open-field arena. They were first presented with two Object A, glass marble or plastic dice, and the exploration time was recorded. After a retention period (3 hours to test short-term memory), the mice were presented again with one object A (ObjectFam) together with a novel object B (ObjectNew), and distinct exploration time for each objects were manually recorded. The calculation was based on the percentage of time spent exploring the new object over total time spent exploring both objects.

### 14.13 **Social recognition**

The social recognition test is quite similar to the novel object recognition test. Instead of objects, the test mouse was left to explore an unfamiliar mouse placed in its proximity. The arena was made up of three identical chambers of identical size (illuminated at 90 Lux), a central chamber with two adjacent chambers connected internally with doors. In each adjacent chamber, a goal box was positioned delimited by a sliding grid. The test mouse was first allowed to freely explore the three chambers for 10 minutes. The test mouse was then placed in the central chamber, and an unfamiliar mouse was briefly introduced into one of the adjacent wire boxes kept closed. The doors of the central chamber were opened; the test mouse explored the three chambers, and its exploratory behavior was recorded for 10 minutes, specifically its preference for each of the wire boxes (with mouse or without mouse). Again, the test mouse was placed in the central chamber, and another unfamiliar mouse (novel mouse) was introduced briefly into the other wire box kept closed. The doors of the central chamber were opened; the test mouse explored the three chambers, and its exploratory behavior was recorded for 10 minutes. Its preference for exploring the wire box of the novel as opposed to the wire box of the familiar mouse was evaluated.

### 14.14 **Social behavior**

Social interaction was evaluated by putting two mice of the same genotype, sex, and weight but from different cages in an open field (dimensions 44.3 × 44.3 × 16.8 cm) illuminated at 50 Lux for a duration of 10 minutes. Several parameters were recorded including the number of sniffing, pawing, following and aggressiveness.

### 14.15 Marbles burying

The marble burying test was used to assess repetitive and perseverative behaviors of the mouse. The chamber consisted of a housing cage filled with five centimeters of bedding on which 24 marbles were equidistantly laid down. The mouse was left in the chamber for 15 minutes. The marbles were categorized as fully covered (100%), partially covered (75%) or not covered. An increase in the number of covered marbles indicate the presence of repetitive and pervasive behaviors.

### **14.16 Circadian activity**

The circadian activity test was used to assess spontaneous activity in a full light/dark cycle. The mouse was put in individual cages (11 × 21 × 18 cm) fitted with IR captors linked to an electronic interface (Imetronic). Locomotor activity was recorded for a period of 32 hours for *Mvp;Mapk3* and *Mvp,* or 35 hours for *Mapk3* single gene mutation. It included a habituation period of 8 or 11 hours, followed by testing for 12 hours each in the dark and light phases. Feeding behavior was also evaluated using a lickometer and pellet feeder (test Diet; Hoffman La-Roche).

### **14.17 Acoustic startle response and pre-pulse inhibition (PPI)**

In mouse, as in humans, unexpected strong acoustic stimuli (pulse) cause startle response. This response can be attenuated when a weaker stimulus is applied right before the pulse (pre-pulse). This principle results from the integration of sensorimotor information, and is impaired in numerous disease such as autism or schizophrenia. In order to measure mouse ability to integrate these signals, the pre-pulse inhibition (PPI) paradigm consisted of exposing the mouse to randomize and repeated acoustic items and of calculating the mouse’s flinching response in a chamber measuring baseline movements over a period of 50 minutes. Portion of the test in shown as example in **Additional file 9: Fig. S17**. Item A consisted of a single acoustic pulse P (60, 70, 75, 85 or 90 dB) presented during 20 milliseconds and the startle response recorded right at the end of the stimulus during 100 milliseconds. Item B comprised a combination of a pre-pulse PP (70, 75, 85 or 90 dB) 80 milliseconds before a loud pulse of 110 dB (lasting 40 milliseconds). In item B, the startle response was recorded only during the 110 dB pulse and the percentage of PPI was calculated by dividing it by the response obtained in item C, at single acoustic pulse 110 dB (ST110 for startle 110), in order to determine the inhibition of the effect that the pre-pulse stimulus exerted over the single startle response at 110 dB: %PPI(X) = PP(X)/ST110*100. Item D consisted of no stimulus (NOSTIM) trials in which only background noise (BN=60 dB) was presented to measure baseline movement of the animal in the chamber. The average response of repeated acoustic items was chosen over the maximum response
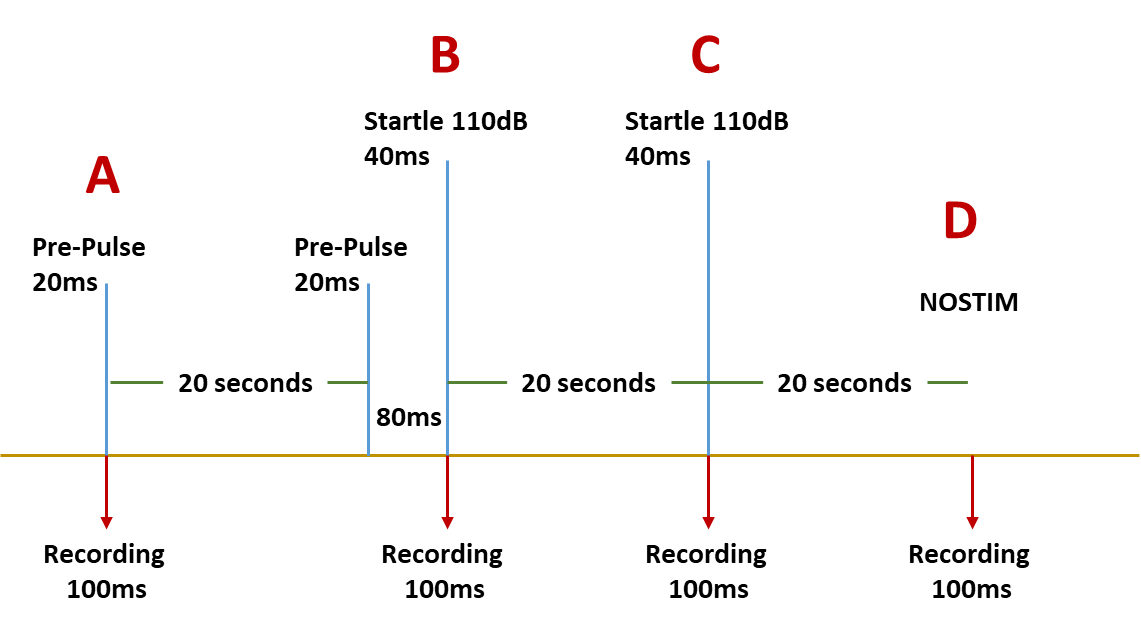
and was given in an arbitrary unit by the software SR-LAB (San Diego Instruments).

**Additional file 9: Fig. S17**. Acoustic startle response and PPI.

### 14.18 Statistics

We assessed statistical significance using GraphPad Prism v8.0.2 (GraphPad Software, Inc., San Diego, CA, USA). All acquired behavioral data were tested for normality (Shapiro-Wilk normality test) and checked for outliers according to the ROUT method (with Q=1%). Outliers are labelled in red in **Additional file 5: Tables S9**, **Additional file 6: Tables S10**, **and Additional file 7: Tables S11**. They were discarded from statistical analysis.

In cohorts consisted of two groups, data were analyzed using Student’s t-test (OF, EPM, TST, PTZ, YM, NOR, SR and CA) or two-way ANOVA for repeated measures followed by Sidak’s post-hoc (OF, FC, Rot, SP, Mar, CA and PPI). Student’s t-tests were used to compare recognition index values to the chance level (50%) (SP, YM, NOR or SR).

The data are represented as the mean ± standard error of the mean (SEM) and the significance threshold was *p*< 0.05 unless otherwise indicated. Significant results were indicated by stars (*:p-value<0.05, ** p-value<0.01, *** p-value<0.001).

**References**

1. Giannuzzi, G. *et al.* The Human-Specific BOLA2 Duplication Modifies Iron Homeostasis and Anemia Predisposition in Chromosome 16p11.2 Autism Individuals. *Am. J. Hum. Genet.* **105**, 947–958 (2019).

2. Mueller, P. *et al.* Regulation of T cell survival through coronin-1-mediated generation of inositol-1,4,5-trisphosphate and calcium mobilization after T cell receptor triggering. *Nat. Immunol.* **9**, 424–431 (2008).

3. Pagès, G. *et al.* Defective thymocyte maturation in p44 MAP kinase (Erk 1) knockout mice. *Science* **286**, 1374–1377 (1999).

4. Chapman, D. L. & Papaioannou, V. E. Three neural tubes in mouse embryos with mutations in the T-box gene Tbx6. *Nature* **391**, 695–697 (1998).

5. Sakaguchi, G. *et al.* Doc2alpha is an activity-dependent modulator of excitatory synaptic transmission. *Eur. J. Neurosci.* **11**, 4262–4268 (1999).

6. Arbogast, T. *et al.* Kctd13-deficient mice display short-term memory impairment and sex-dependent genetic interactions. *Hum Mol Genet* **28**, 1474–1486 (2019).

7. Terakata, M. *et al.* Establishment of true niacin deficiency in quinolinic acid phosphoribosyltransferase knockout mice. *J. Nutr.* **142**, 2148–2153 (2012).

8. Skarnes, W. C. *et al.* A conditional knockout resource for the genome-wide study of mouse gene function. *Nature* **474**, 337–342 (2011).

9. Boroviak, K., Doe, B., Banerjee, R., Yang, F. & Bradley, A. Chromosome engineering in zygotes with CRISPR/Cas9. *Genes. N. Y. N 2000* **54**, 78–85 (2016).

10. Arbogast, T. *et al.* Reciprocal Effects on Neurocognitive and Metabolic Phenotypes in Mouse Models of 16p11.2 Deletion and Duplication Syndromes. *PLoS Genet.* **12**, e1005709 (2016).

11. Collins, S. C. *et al.* Large-scale neuroanatomical study uncovers 198 gene associations in mouse brain morphogenesis. *Nat Commun* **10**, 3465 (2019).

12. Karp, N. A. *et al.* Applying the ARRIVE Guidelines to an In Vivo Database. *PLoS Biol.* **13**, e1002151 (2015).

13. Collins, S. C. *et al.* A Method for Parasagittal Sectioning for Neuroanatomical Quantification of Brain Structures in the Adult Mouse. *Curr Protoc Mouse Biol* **8**, e48 (2018).

14. Martin. M. Cutadapt removes adapter sequences from high-throughput sequencing reads. *EMB-Netjournal* **17**, 10–12 (2011).

15. Langmead, B. & Salzberg, S. L. Fast gapped-read alignment with Bowtie 2. *Nat. Methods* **9**, 357–359 (2012).

16. Dobin, A. *et al.* STAR: ultrafast universal RNA-seq aligner. *Bioinforma. Oxf. Engl.* **29**, 15–21 (2013).

17. Anders, S., Pyl, P. T. & Huber, W. HTSeq--a Python framework to work with high-throughput sequencing data. *Bioinforma. Oxf. Engl.* **31**, 166–169 (2015).

18. Love, M. I., Huber, W. & Anders, S. Moderated estimation of fold change and dispersion for RNA-seq data with DESeq2. *Genome Biol.* **15**, 550 (2014).
