# Supplementary material for: Dissecting the autism-associated 16p11.2 locus identifies multiple drivers in neuroanatomical phenotypes and unveils a male-specific role for the major vault protein
Source: Genome Biol. 2023 Nov 15;24:261. doi: 10.1186/s13059-023-03092-8 (PMC10647150; doi:10.1186/s13059-023-03092-8)
Supplement: Supplementary file 10 — Additional file 10: Supplementary results. Here we present data of i) male NAP genes implicated in two or more brain categories, ii) male NAP genes implicated in one brain category, iii) female NAP genes involved in two or more brain categories, and iv) female NAP genes involved in one brain category. We also show preliminary data on ERK activity measurements. [file 13059_2023_3092_MOESM10_ESM.docx]

ADDITIONAL FILE 10:

Supplementary results

**Dissecting the autism-associated 16p11.2 locus identifies multiple drivers in neuroanatomical phenotypes and unveils a male-specific role for the major vault protein**

Perrine F. Kretz^1^, Christel Wagner^1^, Anna Mikhaleva^2^, Charlotte Montillot^3^, Sylvain Hugel^4^, Ilaria Morella^5^, Meghna Kannan^1^, Marie-Christine Fischer^1^, Maxence Milhau^3^, Ipek Yalcin^4^, Riccardo Brambilla^5,6^, Mohammed Selloum^1,7^, Yann Herault^1,7^, Alexandre Reymond^2^, Stephan C. Collins^1,8^ and Binnaz Yalcin^1,8,*^

^1^University of Strasbourg, CNRS, INSERM, Institute of Genetics and Molecular and Cellular Biology, IGBMC, UMR7104, U964, 67400 Illkirch, France

^2^Center for Integrative Genomics, University of Lausanne, CH-1015 Lausanne, Switzerland

^3^Inserm UMR1231, Université de Bourgogne Franche-Comté, 21000 Dijon, France

^4^Institute of Cellular and Integrative neuroscience, UPR3212, CNRS, 67000 Strasbourg, France

^5^Neuroscience and Mental Health Innovation Institute, School of Biosciences, Cardiff University, CF24 4HQ Cardiff, UK

^6^Dipartimento di Biologia e Biotecnologie “Lazzaro Spallanzani”, Università degli Studi di Pavia, Pavia, Italy

^7^University of Strasbourg, CNRS, INSERM, CELPHEDIA, PHENOMIN, ICS, 67400 Illkirch, France.

^8^Current address: Université de Bourgogne, Inserm UMR1231, 21000 Dijon, France

^*^Correspondence: [binnaz.yalcin@inserm.fr](mailto:binnaz.yalcin@inserm.fr)

## Male NAP genes implicated in two or more brain categories

At the exception of *Mvp* that we described in the main text and **Additional file 1: Fig. S4**, seven other NAP genes (*Bola2*, *Qprt*, *Maz*, *Ppp4c*, *Slx1b*, *Taok2* and *Zg16*) also gave very significant results affecting two or more brain categories (**Fig. 1D**, **Additional file 1: Fig. S2** and **Additional file 4: Table S8**). These findings are described in details below.

Male *Bola2*^+/-^ showed four affected brain parameters pertaining to three main brain categories (commissure, subcortical structures and ventricle). The size of the soma of the corpus callosum was reduced (-10%, *p*=0.0066) concomitant with thin height of the same structure (-11%, *p*=0.044). The size of the hippocampus was also smaller by 12% (*p*=0.018). Intriguingly, the size of the third ventricle was smaller by no less than 40% (*p*=0.0071).

Male *Qprt*^+/-^ showed three affected brain parameters pertaining to two main brain categories (commissure and subcortical). Consistently, the size of the corpus callosum, its width and the size of the internal capsule was smaller by 18% (*p*=0.011), 15% (*p*=0.0032) and 14% (*p*=0.011), respectively. It is worth mentioning that the impact of *Qprt* deletion seems to be specific to white matter structures.

Male *Maz*^+/-^ showed six affected brain parameters pertaining to four main brain categories (all except brain size). Five brain parameters were reduced in size when compared to colony matched WTs including the height of the cingulate cortex (-15%, *p*=0.010), the lateral ventricles (-37%, *p*=0.012), the height of the corpus callosum (-17%, *p*=0.012), the internal capsule (-10%, *p*=0.044) and the habenular nucleus (-14%, *p*=0.046). By contrast, one parameter, the somatosensory cortex at Bregma -1.34 mm was increased in size (+8%, *p*=0.0098).

Male *Ppp4c*^+/-^ associated with ten affected parameters belonging to four main brain categories (all except ventricle). These parameters were all decreased in size and included the total brain area at Bregma +0.98 mm and Bregma -1.34 mm (-10%, *p*=0.0077 and -7%, *p*=0.035, respectively), the height of the cingulate cortex (-7%, *p*=0.0079), the genu of the corpus callosum (-16%, *p*=0.0088), the width of the genu of the corpus callosum (-9%, *p*=0.023), the anterior commissure (-12%, *p*=0.014), the caudate putamen (-10%, *p*=0.019), the somatosensory cortex at Bregma +0.98 mm and Bregma -1.34 mm (-10%, *p*=0.0013 and -3%, *p*=0.014, respectively) and the retrosplenial granular cortex (-9%, *p*=0.029).

Male *Slx1b*^+/-^ exhibited six affected parameters all increased in size related to three brain categories (brain size, commissure and subcortex). The genu of the corpus callosum was enlarged by 11% (*p*=0.031), the total brain area by 7% (*p*=0.031), the soma of the corpus callosum by 10% (*p*=0.018), the height of the corpus callosum b 14% (*p*=0.032), the dorsal hippocampal commissure by 20% (*p*=0.018) and the dentate gyrus by 10% (*p*=0.018).

Male *Taok2*^+/-^ showed six affected parameters spanning three main categories (brain size, cortex and commissure) including three reduced in size and three enlarged in size. While the total brain area was enlarged by 6% (*p*=0.0088), the cingulate cortex by 9% (P=0.0029) and the height of the corpus callosum by 27% (*p*=0.014), the anterior commissure was smaller by 17% (*p*=0.0068), the dorsal hippocampal commissure decreased in size by 15% (*p*=0.009) and the motor cortex smaller by 8% (*p*=0.0065).

Finally, male *Zg16*^+/-^ exhibited seven affected parameters all increased in size pertaining to all categories at the exception of the ventricles. The anterior commissure was increased in size by 26% (*p*=0.004), the total brain area by 7% (*p*=0.011), the anterior commissure by 44% (*p*=0.00083), the fimbria by 13% (*p*=0.015), the habenular nucleus by 10% (*p*=0.039), the arcuate nucleus by 37% (*p*=0.012) and the piriform cortex by 21% (*p*=0.016).

##

## Male NAP genes implicated in one brain category

Five genes (*Doc2a*, *Fam57b*, *Hirip3*, *Spn* and *Tbx6*) presented specific phenotypes, affecting one brain category.

Male *Fam57b*^+/-^ showed smaller cortices across several brain parameters: cingulate (-11%, *p*=0.032), piriform (-18%, *p*=0.018 at Bregma +0.98 mm and -20% *p*=0.004 at Bregma -1.34 mm), retrosplenial (-19%, *p*=0.0018) and motor (-9%, *p*=0.016). By contrast, male *Tbx6*^+/-^ showed thicker cortices: retrosplenial (+11%, *p*=0.000019), piriform (+21%, *p*=0.00069) and somatosensory (+8%, *p*=0.0029). Male *Doc2a*^+/-^ showed enlarged somatosensory cortex (+8%, *p*=0.025), whereas *Spn*^+/-^ thick genu of the corpus callosum (+12%, *p*=0.030) and male *Hirip3*^+/-^ small anterior commissure (-24%, *p*=0.025).

## Female NAP genes involved in two or more brain categories

Six NAP genes (*Slx1b*, *Bola2*, *Gdpd3*, *Tbx6*, *Doc2a* and *Spn*) gave significant results affecting two or more brain categories in female (**Additional file 1: Fig. S3**). These findings are described below.

Female *Slx1b*^+/-^ exhibited three affected parameters all increased in size spanning two brain categories (commissure and brain size). It included an increase of 6% (*p*=0.0084) for the total brain area, 10% (*p*=0.016) for the soma of the corpus callosum and 9% (*p*=0.0024) for the height of the corpus callosum.

Female *Bola2*^+/-^ showed 6 affected parameters all decreased in size, pertaining to three brain categories (commissure, brain size and cortex). The brain size was reduced by 9% (*p*=0.040) concomitant with a thinner somatosensory cortex by 11% (*p*=0.0053). The height of the genu of the corpus callosum was reduced by 15% (*p*=0.029) consistently the soma of the corpus callosum was smaller by 14% (*p*=0.0019) for its area, 11% (*p*=0.043) for its width and 10% (*p*=0.0097) for its height.

Female *Gdpd3*^+/-^ affected three categories (commissure, ventricle and cortex) with an equal number of affected brain parameters. The lateral ventricle was increased by 81% (*p*=0.012), the corpus callosum was thicker by 14% (*p*=0.036) however the motor cortex was smaller by 10% (*p*=0.012).

Female *Tbx6*^+/-^ showed increased measurements across three categories (subcortex, cortex and brain size). The total brain was enlarged by 10% (*p*=0.000056), the fimbria by 13% (*p*=0.00027), the piriform cortex by 16% (*p*=0.045) and the somatosensory cortex by 12% (*p*=0.000045).

Female *Doc2a*^+/-^ had three increased parameters across two categories (commissure and cortex). The cingulate cortex was enlarged by 16% (*p*=0.037), the motor cortex enlarged by 10% (*p*=0.015) and the width of the soma of the corpus callosum longer by 8% (*p*=0.027).

Finally, *Spn*^+/-^ exhibited three increased parameters spanning two categories. The corpus callosum height was thicker by 19% (*p*=0.02), the internal capsule was enlarged by 14% (*p*=0.019) and the fimbria enlarged b 20% (*p*=0.017). Of note, these three brain parameters pertain to white matter structures of the brain.

##

## Female NAP genes involved in one brain category

Four genes (*Ppp4c*, *Fam57b*, *Maz* and *Zg16*) showed specific phenotypes, affecting one single brain category (see **Additional file 1: Fig. S3**).

Female *Ppp4c*^+/-^ showed one associated parameter, the height of the genu of the corpus callosum was diminished by 8% (*p*=0.013). Female *Fam57b*^+/-^ showed a very mild effect on the total brain area (-3%, *p*=0.041). Female *Maz*^+/-^ showed decreased height of the cingulate cortex (-11%, *p*=0.016) and finally, female *Zg16*^+/-^ showed an increased size of the brain (+7%, *p*=0.011).

## ERK activity measurements

We first examined ERK activity by measuring phospho-ERK in the cortex of *Mvp^+/+^;Mapk3^+/+^*, *Mvp^+/+^;Mapk3^+/-^*, *Mvp^+/-^;Mapk3^+/+^* and *Mvp^+/-^;Mapk3^+/-^* mice. Previous work indicated that *Mapk3^-/-^* showed an upregulation of ERK activity^1^. We show that *Mvp^+/+^;Mapk3^+/-^* exhibited an increase of global ERK phosphorylation also (**Additional file 1: Fig. S12M**), upon hemiablation of the gene *Mapk3* confirmed by a 50% reduction of ERK1 protein levels (**Additional file 1: Fig. S12O-P**), while female were unaffected (**Additional file 1: Fig. S12N**). Our quantification of ERK activity revealed a three-fold increase of phospho-ERK in the cortex of *Mvp^+/-^;Mapk3^+/+^*, indicating that MVP could be an inhibitor of ERK signalling. Accordingly, ERK activity was reduced by more than a third in double *Mvp^+/-^;Mapk3^+/-^* mice. These preliminary findings provide *in vivo* evidence of MVP-mediated regulation of ERK signalling, however sex-specific differences in neuroanatomical phenotypes do not seem to be driven by global ERK phosphorylation.

**References**

1. Mazzucchelli, C. *et al.* Knockout of ERK1 MAP kinase enhances synaptic plasticity in the striatum and facilitates striatal-mediated learning and memory. *Neuron* **34**, 807–20 (2002).
