# Supplementary material for: Dissecting the autism-associated 16p11.2 locus identifies multiple drivers in neuroanatomical phenotypes and unveils a male-specific role for the major vault protein
Source: Genome Biol. 2023 Nov 15;24:261. doi: 10.1186/s13059-023-03092-8 (PMC10647150; doi:10.1186/s13059-023-03092-8)
Supplement: Supplementary file 11 — Additional file 11. Summary table of key resources. [file 13059_2023_3092_MOESM11_ESM.docx]

ADDITIONAL FILE 11:

Summary table of key resources

**Dissecting the autism-associated 16p11.2 locus identifies multiple drivers in neuroanatomical phenotypes and unveils a male-specific role for the major vault protein**

Perrine F. Kretz^1^, Christel Wagner^1^, Anna Mikhaleva^2^, Charlotte Montillot^3^, Sylvain Hugel^4^, Ilaria Morella^5^, Meghna Kannan^1^, Marie-Christine Fischer^1^, Maxence Milhau^3^, Ipek Yalcin^4^, Riccardo Brambilla^5,6^, Mohammed Selloum^1,7^, Yann Herault^1,7^, Alexandre Reymond^2^, Stephan C. Collins^1,8^ and Binnaz Yalcin^1,8,*^

^1^University of Strasbourg, CNRS, INSERM, Institute of Genetics and Molecular and Cellular Biology, IGBMC, UMR7104, U964, 67400 Illkirch, France

^2^Center for Integrative Genomics, University of Lausanne, CH-1015 Lausanne, Switzerland

^3^Inserm UMR1231, Université de Bourgogne Franche-Comté, 21000 Dijon, France

^4^Institute of Cellular and Integrative neuroscience, UPR3212, CNRS, 67000 Strasbourg, France

^5^Neuroscience and Mental Health Innovation Institute, School of Biosciences, Cardiff University, CF24 4HQ Cardiff, UK

^6^Dipartimento di Biologia e Biotecnologie “Lazzaro Spallanzani”, Università degli Studi di Pavia, Pavia, Italy

^7^University of Strasbourg, CNRS, INSERM, CELPHEDIA, PHENOMIN, ICS, 67400 Illkirch, France.

^8^Current address: Université de Bourgogne, Inserm UMR1231, 21000 Dijon, France

^*^Correspondence: [binnaz.yalcin@inserm.fr](mailto:binnaz.yalcin@inserm.fr)

| REAGENT or RESOURCE | SOURCE | IDENTIFIER |
| --- | --- | --- |
| Biological Samples | Brain samples of mutant mouse lines | **Additional file 2: Table S1** |
| Chemicals, Peptides, and Recombinant Proteins | | |
| Cresyl violet acetate | Sigma-Aldrich | Cat#C5042-106 |
| Solvent Blue 38 | Sigma-Aldrich | Cat#S3382-25G |
| Lithium carbonate | Sigma-Aldrich | Cat#13010-100G-R |
| Oxalic acid | Sigma-Aldrich | Cat#241172-50G |
|  |  |  |
| Deposited Data | | |
| Assessed brain parameters | This paper | **Additional file 2: Tables S2, S5, S6** |
| Comprehensive heat map of neuroanatomical defects | This paper | **Additional file 4: Table S8** |
| Software and Algorithms | | |
| FileMakerPro version 14.0.6 | FileMaker Inc. | https://www.filemaker.com/ |
| ImageJ/Fiji version 1.51e | Schneider et al., 2012 | <http://imagej.nih.gov/ij/> |
| Ensembl (Human GRCh38.p13; Mouse: GRCm38.p6) | Ensemble.org | https://www.ensembl.org/ |
| NPDview2.0 | Hamamatsu | https://www.hamamatsu.com/ |
| Other | | |
| Expression datasets |  | **Additional file 2: Table S4** |
| Mouse whole body phenotypes | The Jackson Laboratory | http://www.informatics.jax.org/downloads/reports/index.html#pheno |
