# Supplementary material for: Dissecting the autism-associated 16p11.2 locus identifies multiple drivers in neuroanatomical phenotypes and unveils a male-specific role for the major vault protein
Source: Genome Biol. 2023 Nov 15;24:261. doi: 10.1186/s13059-023-03092-8 (PMC10647150; doi:10.1186/s13059-023-03092-8)
Supplement: Supplementary file 12 — Additional file 12. Uncropped images for the cropped blots in Fig. 3 and Additional file 1: Fig S5, S6 and S12. [file 13059_2023_3092_MOESM12_ESM.docx]

**Additional file 12: uncropped images for the cropped blots in Figure 3, Additional file 1: Fig S5, S6 and S12.**

For each Figure, RED RECTANGLES INDICATE the approximate REGION OF THE GELS/blots SELECTED FOR PUBLICATION.

- **Figure 3**

4 files provided per protein (MVP and b-actin) corresponding to pictures from biorad.

Because of issues with samples 2 (damaged band) and 4 (genotyping uncertainty found later), samples 1,3,5,6,7,8,9 were used for publication.

File Names are provided and bear the prefix Fig3B_*


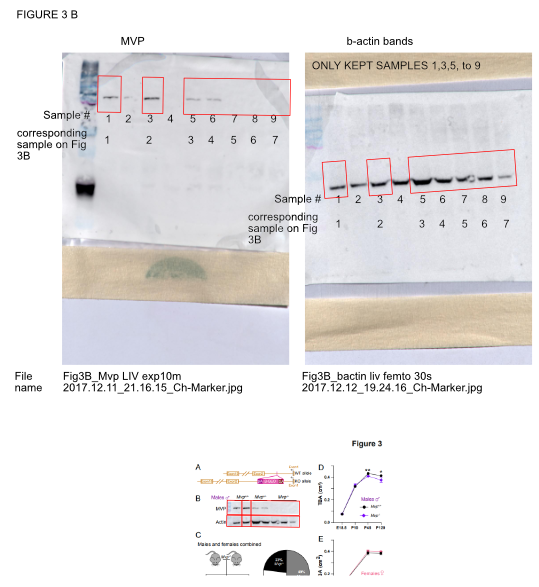


- **Additional file 1: Fig S5**

3 pdf files are provided and bear the prefix ExtFig5A_*, ExtFig5B_* (note same as ExtFig6b_*), ExtFig5C_*

Additional file 1: Fig S5A


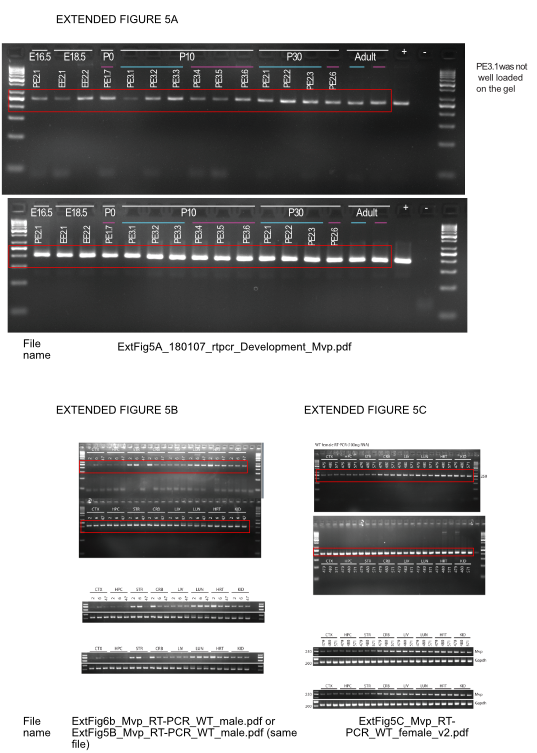


Additional file 1: Fig S5B Additional file 1: Fig S5C


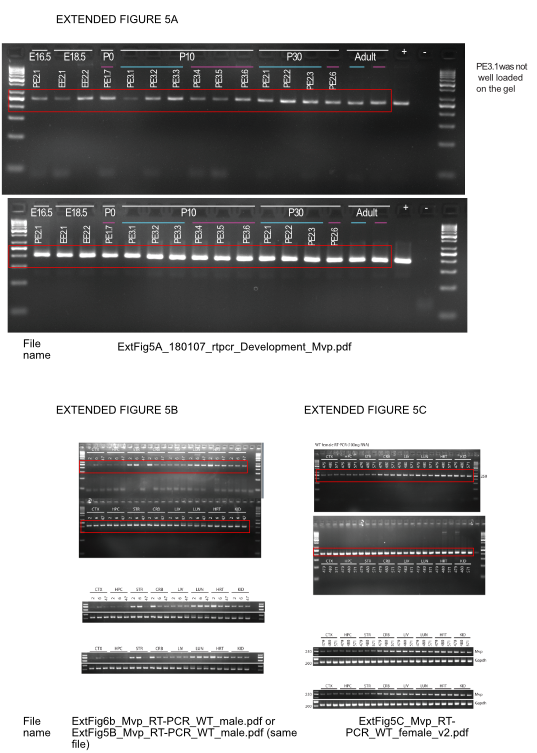


- **Additional file 1: Fig S6**

3 pdf files are provided and bear the prefix ExtFig6a_*, ExtFig6b_*, ExtFig6C_*

Additional file 1: Fig S6B

**
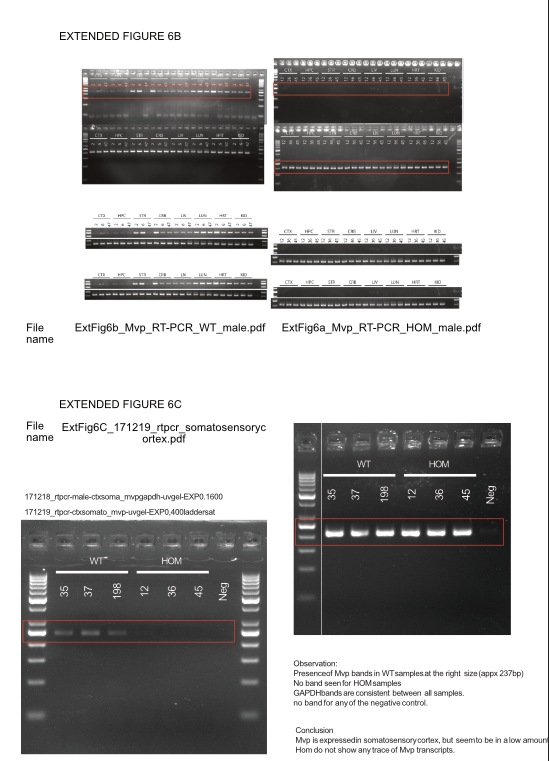
**

Additional file 1: Fig S6C

**
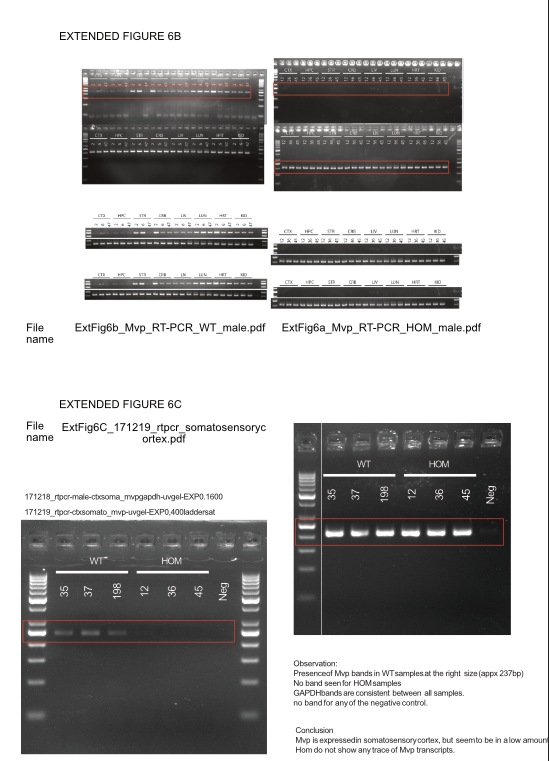
**

- **Additional file 1: Fig S12**

4 .scn files (can be opened via ImageJ) are provided corresponding to different exposition time and bear the prefix ExtFig12Oa_*, ExtFig12Ob_*, ExtFigPa_*, ExtFig12Pb_*

Additional file 1: Fig S12O


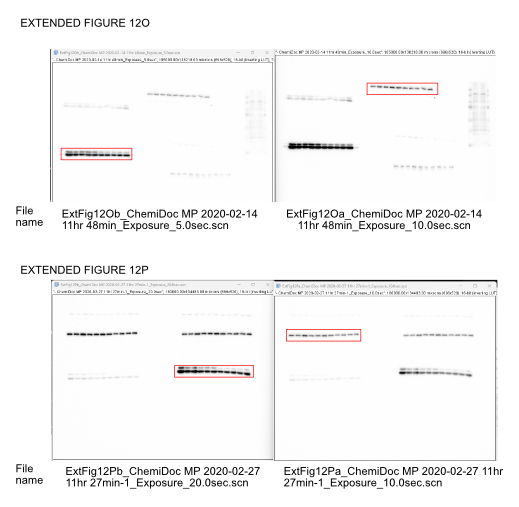


Additional file 1: Fig S12P


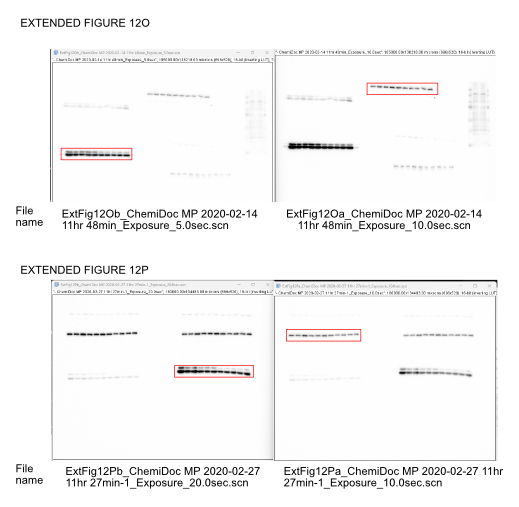


>Please provide a reference for your datasets in Figshare and GEO

See below for our replies.

We followed Genome Biology repositories for the GEO. For the microscopy images, we have acquired a very large number of images for this project (more than thousands). These images were left in the IGBMC data archiving disks before our lab’s move to Dijon. Retrieving all these data is an enormous task and we are not sure whether this will be possible in a reasonable timeframe. However, we have added a new Additional file 3 that contains all the explanations in great details of how we collected the data from these images and provide all the raw data in great details. Representative microscopy images are available throughout the manuscript (Figures 1-4 and Additional file 1: Figs. S1, S5, S9.

Yours sincerely,


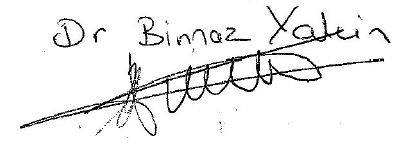


Dr Binnaz Yalcin

Inserm Unit 1231, 15 boulevard Maréchal De Lattre de Tassigny, 21070 Dijon 
Tel: +33 (0)3 80 39 66 60
Fax: +33 (0)3 80 39 66 00
Email: [binnaz.yalcin@inserm](mailto:binnaz.yalcin@inserm).fr

Lab web site: <https://blog.u-bourgogne.fr/yalcingroup/>
