# Supplementary material for: Dissecting the autism-associated 16p11.2 locus identifies multiple drivers in neuroanatomical phenotypes and unveils a male-specific role for the major vault protein
Source: Genome Biol. 2023 Nov 15;24:261. doi: 10.1186/s13059-023-03092-8 (PMC10647150; doi:10.1186/s13059-023-03092-8)
Supplement: Supplementary file 1 — Additional file 1: Fig. S1. Standardized workflow for neurophenotyping. Fig. S2. Heat map of gene association with NAPs in males. Fig. S3. Heat map of gene association with NAPs in females. Fig. S4. Analysis of Mvp, Mapk3 and Kctd13 mice.Fig. S5. Mvp expression profiling. Fig. S6. Mvp knockout model validation and viability screen. Fig. S7. Impact of Mvp deletion on neuromorphology in females. Fig. S8. Impact of Mvp deletion on neuromorphology in males. Fig. S9. Analysis of Mvp loss in the cingulate gyrus. Fig. S10. Behavioral studies of Mvp homozygous male mice. Fig. S11. Behavioral studies of Mapk3 heterozygous male mice. Fig. S12. Behavioral studies of DKO of Mvp and Mapk3 genes. [file 13059_2023_3092_MOESM1_ESM.docx]

ADDITIONAL FILE 1:

Supplementary figures

**Dissecting the autism-associated 16p11.2 locus identifies multiple drivers in neuroanatomical phenotypes and unveils a male-specific role for the major vault protein**

Perrine F. Kretz^1^, Christel Wagner^1^, Anna Mikhaleva^2^, Charlotte Montillot^3^, Sylvain Hugel^4^, Ilaria Morella^5^, Meghna Kannan^1^, Marie-Christine Fischer^1^, Maxence Milhau^3^, Ipek Yalcin^4^, Riccardo Brambilla^5,6^, Mohammed Selloum^1,7^, Yann Herault^1,7^, Alexandre Reymond^2^, Stephan C. Collins^1,8^ and Binnaz Yalcin^1,8,*^

^1^University of Strasbourg, CNRS, INSERM, Institute of Genetics and Molecular and Cellular Biology, IGBMC, UMR7104, U964, 67400 Illkirch, France

^2^Center for Integrative Genomics, University of Lausanne, CH-1015 Lausanne, Switzerland

^3^Inserm UMR1231, Université de Bourgogne Franche-Comté, 21000 Dijon, France

^4^Institute of Cellular and Integrative neuroscience, UPR3212, CNRS, 67000 Strasbourg, France

^5^Neuroscience and Mental Health Innovation Institute, School of Biosciences, Cardiff University, CF24 4HQ Cardiff, UK

^6^Dipartimento di Biologia e Biotecnologie “Lazzaro Spallanzani”, Università degli Studi di Pavia, Pavia, Italy

^7^University of Strasbourg, CNRS, INSERM, CELPHEDIA, PHENOMIN, ICS, 67400 Illkirch, France.

^8^Current address: Université de Bourgogne, Inserm UMR1231, 21000 Dijon, France

^*^Correspondence: [binnaz.yalcin@inserm.fr](mailto:binnaz.yalcin@inserm.fr)

**Fig S1.** Standardized workflow for neurophenotyping.

The workflow contains six items corresponding to the mouse itself, the brain sample, the paraffin-embedded blocs, the brain images, the quantitative measures and the heat map. Each of these items are interconnected by specific standard operating procedures. i) Mouse: Mutant mice were either produced by the partners of the International Mouse Phenotyping Consortium (IMPC) or acquired through collaboration (**Additional file 2: Table S1**). Mouse colonies were maintained on a pure inbred C57BL/6 background and brain tissues were carefully harvested at 16 weeks of age. ii) Brain: Samples were fixed in 10% buffered formalin for 48 hours and trimmed for paraffin embedding. iii) Blocs: Paraffin blocs were sectioned with a sliding microtome (Microm HM 450) at a thickness of 5µm. Sections were double-stained using Nissl (violet) for neurons and Luxol (blue) for myelin, generating a color contrast between white and grey matters. iv) Images: Whole slides were scanned using a digital slide Hamamatsu Nanozoomer scanner (2.0HT C9600 series), producing whole brain images at cell-level resolution. Each image was checked for stringent quality parameters including symmetry and position relative to the defined sections. v) Measures: Neurophenotyping parameters were measured using Fiji 1.51e across the two stereotactically defined coronal sections (**Additional file 2: Table S2**). At Bregma +0.98 mm and Bregma -1.34 mm, 22 and 45 parameters were measured, respectively. Measurements and quality control were made within a relational database interface in FileMaker Pro 14.0.6. To test for any potential co-variate structure effect, experimental co-variates were also recorded, for example sample processing dates and usernames were collected at every step of the procedure. Extensive quality control checks and critical evaluation of the dataset were carried out. Gene association was considered as significant when the p-value was smaller than 0.05. All significant gene associations were critically verified by an independent experimenter with extensive expertise in brain anatomy. vi) Heat map: A color-coded heat map of neuroanatomical defects including percentage change and corresponding p-value was generated once the above steps were completed. NAP genes were classified based on the number of brain categories affected as one or multiple.

**Fig S2.** Heat map of gene association with NAPs in males.

Brain parameters are shown along the horizontal axis and studied alleles along the vertical axis. Color intensity indicates the strength of the association based on the p-value significance threshold. Blue color refers to a brain region that is increased in size while red shows a region decreased in size. More details are available in **Additional file 4: Table S8**. In the studied allele names along the vertical axis, HET refers to heterozygous knockout alleles, HOM to homozygous and WT to wild-type littermate control mice.

**Fig S3.** Heat map of gene association with NAPs in females.

Brain parameters are shown along the horizontal axis and studied alleles along the vertical axis. Color intensity indicates the strength of the association based on the p-value significance threshold. Blue color refers to a brain region that is increased in size while red shows a region decreased in size. More details are available in **Additional file 4: Table S8**. In the studied allele names along the vertical axis, HET refers to heterozygous knockout alleles, HOM to homozygous and WT to wild-type littermate control mice.

**Fig S4.** Analysis of *Mvp*, *Mapk3* and *Kctd13* mice*.*

Each panel represents results obtained from quantitative histomorphological analysis of heterozygous female *Mvp^+/-^* (**A**), heterozygous male *Mapk3^+/-^* (**B**) and heterozygous (**C**) and homozygous (**D**) male *Kctd13* mice. Two different genetic models of *Kctd13* were used (<Nk> and <Ics> explained in **Additional file 2: Table S1**). *Left*. Schematic representation of the 24 brain regions quantified at Bregma +0.98 mm and -1.34 mm on coronal sections. Colored regions indicate the presence of at least one significant parameter within the brain region at the 0.05 level. White indicates a p-value > 0.05, grey shows not enough data to calculate a p-value. *Right.* Histograms of percentage change relative to matched WT (set as 0) for each of the measured parameters (listed in **Additional file 2: Table S2**). For complete data sets (% change and p-value) of male and female neuroanatomical studies, see **Additional file 4: Table S8**.

**Fig S5.** *Mvp* expression profiling.

(**A**) Reverse transcriptase PCR assessing *Mvp* expression in whole murine brain extracts from WT mice at different developmental stages, ranging from embryonic day 16.5 (E16.5), to E18.5, post-natal day 0 (P0), P10, P30 and up to adulthood (P210). Blue and pink bars indicate male and female samples, respectively. (**B-C**) RT-PCR assessing *Mvp* expression in male (**B**) and female (**C**) extracts. Four brain regions were tested including the cortex (CTX), the hippocampus (HP), the striatum (STR) and the cerebellum (CRB), as well as four peripheral organs: the liver (LIV), the lung (LUN), the heart (HRT) and the kidney (KID). Three biological replicates were used. *Gapdh* was used as loading control. (**D-F**) IHF of MVP, NeuN neurons and Hoechst on brain sections from WT male (**D**, **E** and **F**) and WT female (**D’**, **E’** and **F’**) mice. Pictures from somatosensory cortex (**D** and **D’**), piriform cortex (**E** and **E’**) and cerebellum (**F** and **F’**) shown. Dashed square delineates the region presented at higher-magnification on individual channels for MVP, NeuN and Hoechst, and merge of the three. MVP signal came from the cytoplasm with no overlap with the neuronal nuclei, NeuN, marker.

**Fig S6.** *Mvp* knockout model validation and viability screen.

(**A**) Schematic representation of *Mvp* knockout model construction. Murine *Mvp* gene, located on the reverse strand on chromosome 7 from position 126,986,860 to 127,014,621 (spanning 27,761bp) on Build GRCm38.p6 of the mouse genome, contains 15 exons. The knockout mutants were obtained by a promoter gene trap design, resulting in the insertion of a β-galactosidase/neomycin phosphotransferase (β-GEO) cassette within the first intron of *Mvp* gene. The β-GEO cassette comprises a splice acceptor (SA) allowing the expression of the reporter as well as drug resistance. The polyA-tail (pA) serves to end the transcription, and thus discard the transcription of *Mvp* gene. (**B**) Validation of *Mvp* knockout at the transcript level. RT-PCR for *Mvp* transcript from *Mvp^+/+^* (top panel) and *Mvp^-/-^* (middle and bottom panels). Four brain regions (cortex (CTX), hippocampus (HP), striatum (STR) and cerebellum (CRB)) and four peripheral organs (liver (LIV), lung (LUN), heart (HRT) and kidney (KID)) were tested on three biological replicates. *Gapdh* transcripts were used as positive control. (**C**) RT-PCR from somatosensory cortex extracts of *Mvp^+/+^* and *Mvp^-/-^* mice (three replicates each) providing further evidence of a complete full knockout model. Negative control (Neg) was set with water. (**D**) Validation of *Mvp* knockout at the protein level. *Left*. Western blot (WB) from liver extracts of *Mvp^+/+^* (n=2), *Mvp^+/-^* (n=2) and *Mvp^-/-^* (n=3) mice. *Right*. Quantification of the WB membrane. Actin was used as loading control for normalization and *Mvp^+/+^* mean set as one for ease of readability of the graph. (**E**) Specificity of *Mvp* deletion on transcription. Design of the different primers (indicated in color at the top of the scheme) and reactions (defined by a number with the corresponding color according to primer association) used to attest the presence of the different transcripts. The size of the expected band is indicated below each reaction. Reaction 1 (Genomic DNA) was specific to genomic DNA, and served as negative control. Reaction 2 (Mvp_202) enabled to see *Mvp_202­* (long isoform) expression. Reaction 3 (Mvp_Com) amplified a fragment of transcript that is common to *Mvp_202­* and *Mvp_201* isoforms. Reaction 4 (Pagr1a/b) amplified a fragment of transcript that is common to *Pagr1a* and *Pagr1b* isoforms. Reaction 5 (Pagr1b) is specific to *Pagr1b* transcript. RT-PCR for each of these reactions were conducted according to protocols defined in **Additional file 9** on two biological replicates in male *Mvp^+/+^* and *Mvp^-/-^* mice, in cortex (CTX) and liver (LIV) extracts. The table recapitulates the results obtained: Ø when no bands were seen, +, ++ and +++, for faint, medium and strong band, respectively. (**F**) Viability screen of *Mvp* knockout mice. *Left.* Schematic representation of heterozygous-by-heterozygous breeding scheme, with expected Mendelian ratio of segregation of the offspring (25% ^+/+^; 50% ^+/-^; 25% ^-/-^). *Center.* Graph represents the ratio of each genotype of successfully genotyped mice obtained from heterozygous-by-heterozygous breeding (n=617). Proportions followed Mendelian’s ratio, thus excluding any mortality due to the presence of the mutation. *Right.* The sex of each mouse also segregated according to 50%/50% expected sex ratio, thus excluding influence of the mutation on viability dependent upon gender.

**Fig S7.** Impact of *Mvp* deletion on neuromorphology in females.

(**A**) *Left*. Schematic representation of the 24 brain regions quantified at Bregma +0.98 mm and -1.34 mm on coronal section from *Mvp^+/+^* and *Mvp^-/-^* mice. Colored regions indicate the presence of at least one significant parameter within the brain region at the 0.05 level. White indicates a p-value > 0.05, grey shows not enough data to calculate a p-value. *Right.* Histograms of percentage change relative to *Mvp^+/+^* (set as 0) for each of the measured parameters. (**B-G**) Cell count and average cell area determined in different regions of the murine brain: anterior cingulate cortex (**B**), anterior somatosensory cortex (**C**), posterior somatosensory cortex (**D**), caudate putamen (**E**), motor cortex (**F**) and retrosplenial cortex (**G**). Plots are represented as mean ± SEM. Two-tailed Student *t* test equal variance. **p*<0.05.

**Fig S8.** Impact of *Mvp* deletion on neuromorphology in males.

(**A-F**) Cell count and average cell area determined in different regions of the murine brain: layers of the anterior somatosensory cortex (**A**), posterior somatosensory cortex (**B**), Caudate Putamen at Bregma +0.98 mm (**C**), layers of the motor cortex at Bregma +0.98 mm (**D**), motor cortex at Bregma +0.98 mm (**E**) and retrosplenial cortex at Bregma -1.34 mm (**F**). (**G**) Cell count and average cell area in the cingulate gyrus of *Mvp^+/+^* and *Mvp^-/-^* mice on sagittal section at Lateral +0.60mm. (**H**) Cell density of Purkinje cells in cerebellum was calculated by dividing the number of Purkinje cells (PC; blue dots) by the total length of the granular layer of the cerebellum (red line) from *Mvp^+/+^* and *Mvp^-/-^* mice. Plots are represented as mean ± SEM. Two-tailed Student *t* test equal variance (**B-C, E-H**). For layer analysis (**A** and **D**), two-tailed Student *t* tests were corrected for multiple comparisons using the Holm-Sidak method. **p*<0.05.

**Fig S9.** Analysis of *Mvp* loss in the cingulate gyrus.

(**A-C**) Electrophysiological readouts: amplitude (**A**) and inter-event interval (**B**) of miniature excitatory post-synaptic currents (mEPSCs), and (**C**) capacitance, measured by patch-clamp on neurons from cingulate gyrus from *Mvp^+/+^* and *Mvp^-/-^* male mice. (**D**) *Left*. Spine density quantifications from dendrites of pyramidal neurons in the cortex from *Mvp^+/+^* and *Mvp^-/-^* male mice. *Right*. Representative Golgi images. (**E**) Pictures of sections (2µm thick) of cingulate gyrus of *Mvp^+/+^* and *Mvp^-/-^* male mice stained with toluidine blue. (**F**) Electronic microscopy from cingulate gyrus of *Mvp^+/+^* and *Mvp^-/-^* mice. N, nucleus; M, mitochondria; Ly, Lysosome. (**G**) Scatter-plot comparing the mean normalized counts for each condition. A pseudo count of 1 was added to all values in order to represent genes that are not expressed in one condition. Significant genes were selected using the following thresholds: adjusted p-value lower than 0.05 and absolute value of log2 Fold-Change greater than 1. Only *Mvp* (red dot) expression was significantly affected in *Mvp^-/-^* (n=6) compared to *Mvp^+/+^* (n=6) male mice.

**Fig S10.** Behavioral studies of *Mvp* homozygous male mice.

(**A**) Open field test. *Left*. Distance travelled, recorded in centimeters every five minutes, over the time of the test (30 minutes). *Middle*. Percentage of distance travelled in the center of the arena over total distance travelled. *Right*. Latency in seconds before the first entrance in the center of the arena. (**B**) Elevated plus maze. *Left*. Number of entrances in the open arm. *Middle*. Percentage of time spent in the open arm over total time of the test (5 minutes). *Right*. Number of times the mice bend its nose to the ground over the open arm. (**C**) Fear conditioning test. *Conditioning.* Freezing time (in seconds) during the first 4 minutes (Hab-1 and Hab-2, two minutes each) and after unconditional stimulus (POST-US, 2 minutes). *Context.* Freezing time (in seconds) of the mice when replaced in the same context after 24 hours (Cont-1-3, two minutes each). *Cue*. Freezing time (in seconds) during the different phase (two minutes each) of the cued test. The cue (light and sound) is presented during the entire Cue1 and Cue2 phase. (**D**) Latency in seconds before the first immobility of the mouse in the tail suspension test. (**E**) Pentylenetetrazol (PTZ)-test. Duration of the clonic phase of status epilepticus after PTZ intraperitoneal injection. (**F**) Rotarod test. *Learning*. Time (in seconds) spent on the rotarod at increasing speed from 4 to 40 rotation per minute (rpm) over five minutes, during four sessions on three consecutive days. *Test.* On the day of testing, latency before falling off the rotarod during different fixed rotation speeds (4, 10, 16, 22, 28, 34 and 40 rpm). Maximum time per trial was set to 120 seconds. (**G**) Sucrose preference was measured as the percentage of sucrose consumption over the total consumption during habituation (Hab, 1h), and testing days (Test-Day1 and Test–Day2, 12 hours during dark cycle). (**H**) Y-maze. Percentage of alternation was calculated by dividing the number of alternations by the number of possible triads x 100. (**I**) Novel Object Recognition (NOR). *Exploration.* Exploration time (in seconds) of the different objects (ObjectA in 1st phase, and ObjectFam and ObjectNew, in 2nd phase). *Recognition*. Percentage of exploration of the new object over total object exploration (ObjectNew/(ObjectNew+ObjectFam)). 50% was considered as no recognition of the familiar object. (**J**) Three-chamber test. Percentage of the duration of interaction with the Old Individual (OldInd) or New Individual (NewInd) over total interaction time during the 3^rd^ phase of the test. (**K**) Marble: Plot represents the number of marbles according to their level of coverage (75%Cover, 100%Cover or NoCover). (**L**) Circadian Activity. *Locomotion.* Tracking of locomotor activity (in centimeters) every hour over the duration of the test (32 hours). Statistical analysis was performed over the combined Dark and Light phase (from 7pm to 7pm). *Pellet* and *Water* consumptions. Weight of pellets and water in grams consumed over 32 hours. (**M**) Pre-Pulse Inhibition. *Startle* *response*. Averaged amplitude of the acoustic startle reflex (arbitrary unit) following 60 dB (Background Noise = BN), 70 dB pulse (P70), 75 dB (P75), 85 dB (P85), 90 dB (P90) and 110 dB (ST110) acoustic stimulus. PPI: Percentage of Pre-Pulse (PP) Inhibition calculated as PPX %=( PPX-ST110)/ST110. (**N**) Body weight. Body weight tracking (in grams) from 15 to 24 weeks of age. *Mvp*^+/+^ are represented by black dots and *Mvp*^-/-^ by purple diamonds. A comprehensive description of behavioral tests and raw datasets are provided in **Additional file 9** and **Additional file 6: Table S10**, respectively. Mean and standard error of the mean are shown in the graphs. Sample size is indicated within each histogram. Two-tailed Student t test equal variance (**A**, **B**, **D**, **E**, **G**, **H**, **I**, **J**, **K**, **L** and **M**) and two-way ANOVA with Sidak post hoc (**A**, **C**, **F**, **L** and **N**).

**Fig S11.** Behavioral studies of *Mapk3* heterozygous male mice.

(**A**) Open field test. *Left*. Distance travelled, recorded in centimeters every five minutes, over the time of the test (30 minutes). *Middle*. Percentage of distance travelled in the center of the arena over total distance travelled. *Right*. Latency in seconds before the first entrance in the center of the arena. (**B**) Elevated plus maze. *Left*. Number of entrances in the open arm. *Middle*. Percentage of time spent in the open arm over total time of the test (5 minutes). *Right*. Number of times the mice bend its nose to the ground over the open arm. (**C**) Fear conditioning test. *Conditioning.* Freezing time (in seconds) during the first 4 minutes (Hab-1 and Hab-2, two minutes each) and after unconditional stimulus (POST-US, 2 minutes). *Context.* Freezing time (in seconds) of the mice when replaced in the same context after 24 hours (Cont-1-3, two minutes each). *Cue*. Freezing time (in seconds) during the different phase (two minutes each) of the cued test. The cue (light and sound) is presented during the entire Cue1 and Cue2 phase. (**D**) Latency in seconds before the first immobility of the mouse in the tail suspension test. (**E**) Pentylenetetrazol (PTZ)-test. Duration of the clonic phase of status epilepticus after PTZ intraperitoneal injection. (**F**) Rotarod test. *Learning*. Time (in seconds) spent on the rotarod at increasing speed from 4 to 40 rotation per minute (rpm) over five minutes, during four sessions on three consecutive days. *Test.* On the day of testing, latency before falling off the rotarod during different fixed rotation speeds (4, 10, 16, 22, 28, 34 and 40 rpm). Maximum time per trial was set to 120 seconds. (**G**) Sucrose preference was measured as the percentage of sucrose consumption over the total consumption during habituation (Hab, 1h), and testing days (Test-Day1 and Test–Day2, 12 hours during dark cycle). (**H**) Y-maze. Percentage of alternation was calculated by dividing the number of alternations by the number of possible triads x 100. (**I**) Novel Object Recognition (NOR). *Exploration.* Exploration time (in seconds) of the different objects (ObjectA in 1st phase, and ObjectFam and ObjectNew, in 2nd phase). *Recognition*. Percentage of exploration of the new object over total object exploration (ObjectNew/ (ObjectNew+ObjectFam)). 50% was considered as no recognition of the familiar object. (**J**) Three-chamber test. Percentage of the duration of interaction with the Old Individual (OldInd) or New Individual (NewInd) over total interaction time during the 3^rd^ phase of the test. (**K**) Marble: Plot represents the number of marbles according to their level of coverage (75%Cover, 100%Cover or NoCover). (**L**) Circadian Activity. *Locomotion.* Tracking of locomotor activity (in centimeters) every hour over the duration of the test (32 hours). Statistical analysis was performed over the combined Dark and Light phase (from 7pm to 7pm). (**M**) Pre-Pulse Inhibition. *Startle* *response*. Averaged amplitude of the acoustic startle reflex (arbitrary unit) following 60 dB (Background Noise = BN), 70 dB pulse (P70), 75 dB (P75), 85 dB (P85), 90 dB (P90) and 110 dB (ST110) acoustic stimulus. PPI: Percentage of Pre-Pulse (PP) Inhibition calculated as PPX %=( PPX-ST110)/ST110. (**N**) Body weight. Body weight tracking (in grams) from 15 to 24 weeks of age. *Mapk3*^+/+^ are represented by black dots and *Mapk3*^+/-^ by blue triangle. A comprehensive description of behavioral tests and raw datasets are provided in **Additional file 9** and **Additional file 7: Table S11**, respectively. Mean and standard error of the mean are shown in the graphs. Sample size is indicated within each histogram. Two-tailed Student t test equal variance (**A**, **B**, **D**, **E**, **G**, **H**, **I**, **J**, **K** and **M**) and two-way ANOVA with Sidak post hoc (**A**, **C**, **F**, **L** and **N**). **p*<0.05.

**Fig S12.** Behavioral studies of DKO of *Mvp* and *Mapk3* genes.

(**A**) Weekly body weight tracking from age 11- to 25 weeks in male *Mvp^+/-^;Mapk3^+/-^* and *Mvp^+/+^;Mapk3^+/+^* mice (n=12 per group). (**B**) Grip strength was evaluated on either the four (*left*) or the two anterior (*right*) paws, and normalized on mouse body weight. (**C**) Rotarod test. *Learning*. Time (in seconds) spent on the rotarod at increasing speed from 4 to 40 rotation per minute (rpm) over five minutes, during four sessions on three consecutive days. *Latency before falling*. On the day of testing, latency before falling off the rotarod at rotation speeds (4, 10, 16, 22, 28, 34 and 40 rpm). Maximum per trial was set to 120 seconds. (**D**) Sucrose preference was measured as the percentage of sucrose consumption over the total consumption during, habituation (Hab, 1h), and testing days (Test-Day1 and Test–Day2, 12h during dark cycle). (**E**) Y-maze. Percentage of alternation was calculated by dividing the number of alternations by the number of possible triads x 100. (**F**) Novel Object Recognition (NOR). *Exploration*. Exploration time (in seconds) of the different objects (ObjectA in 1st phase, and ObjectFam and ObjectNew, in 2^nd^ phase). *Recognition*. Percentage of exploration of the new object over total object exploration (ObjectNew/ (ObjectNew+ObjectFam)). 50% is considered as no recognition of the familiar object. (**G**) Three-chamber test. Percentage of the duration of interaction with the Old Individual (*OldInd*) or New Individual (*NewInd*) over total interaction time during the 3^rd^ phase. (**H**) Social Interaction. Graphs plot the duration of pawing, following, interaction and sniffing behaviors between two individuals from the same genotype. (**I**) Marble. Plot represents the number of marbles according to their level of coverage (75%cover, 100%Cover or NoCover). (**J**) Circadian Activity. *Locomotion.* Tracking of locomotor activity (in cm) every hour over the duration of the test (32h). Statistical analysis is performed over combined Dark and Light phase (from 7pm to 7am). *Pellet* and *Water*. Weight of pellets and water in grams consumed over 32 hours. (**K**) Pre-Pulse Inhibition. *Startle response*. Averaged amplitude of the acoustic startle reflex (arbitrary unit) following 60dB (Background Noise), 70dB (P70), 75dB (P75), 85dB (P85), 90dB (P90) and 110dB (ST110) acoustic stimulus. *PPI*. Percentage of Pre-Pulse Inhibition calculated as PPX %=(PPX-ST110)/ST110. (**L**) Open-Field. Latency to enter the center of the arena. A comprehensive description of behavioral tests, raw datasets and additional findings are provided in **Additional file 9**, **Additional file 8: Table S12** and **Figure 5**, respectively. (**M-N**) Number of phospho-ERK positive neurons quantified through the prefrontal cortex by immunofluorescence in male (**M**) and female (**N**). Mean and standard error of the mean are shown in the graphs. (**O**-**P**) *Left*. Western blot analysis of ERK-expression on proteins extracted from cortical tissues in male (**O**) and female (**P**) showing ERK1 and ERK2 differential expression levels in five *Mvp^+/-^;Mapk3^+/-^* compared to five matched *Mvp^+/+^;Mapk3^+/+^* in each group. *Right*. Quantification of the Western blots relative to *Mvp^+/+^;Mapk3^+/+^*. Normalization was done on vinculin expression. Bullet points represent *Mvp^+/+^;Mapk3^+/+^* mice and square *Mvp^+/-^;Mapk3^+/-^*. Males are represented in blue and females in pink. Mean and standard error of the mean are shown in the graphs. Two-tailed Student *t* test equal variance (**B**, **D-K** and **O**-**P**) and two-way ANOVA with Sidak post hoc (**A**, **C** and **K**). One-way ANOVA with Tukey post hoc (**M**-**N**). **p*<0.05 ***p*<0.01.
