# Supplementary material for: Dissecting the autism-associated 16p11.2 locus identifies multiple drivers in neuroanatomical phenotypes and unveils a male-specific role for the major vault protein
Source: Genome Biol. 2023 Nov 15;24:261. doi: 10.1186/s13059-023-03092-8 (PMC10647150; doi:10.1186/s13059-023-03092-8)
Supplement: Supplementary file 13 — Additional file 13. Review history. [file 13059_2023_3092_MOESM13_ESM.docx]

Review History

**First round of review**

**Reviewer 1**

**Were you able to assess all statistics in the manuscript, including the appropriateness of statistical tests used?**

Yes, and I have assessed the statistics in my report.

**Comments to author:**

This study used an impressive number of mouse models of mutations in 20 different genes within the syntenic region of human 16p11.2 and performed extensive neuroanatomical phenotyping. They identify the major vault protein as a major contributor to microcephaly in males, despite no apparent differences in behavior. They also attempt to make a connection to Mapk3 which is another gene in the locus, but the results shown are not particularly convincing. Overall, the study is potentially important for others in the 16p11.2 field, but as presented the results are confusing and do not substantiate the claims made in the abstract.

Major concerns

1. Neither Figure 2 nor the legend mention the sex of the mice in the figure and the Results section related to Fig 2 does not either, despite starting with "we thought to determine the origin of Mvp sex-specific differences in brain size." This appears to leave a major question in this study unanswered, namely, is there a sex difference in the level of Mvp protein or transcript in any of the brain regions where it is expressed? There is a statement in the Results claiming "no apparent sex differences were detected in the pattern of MVP/vault expression," but there is no data showing this conclusion. Furthermore, the potentially very important conclusion that the male Mvp-/- mice were unique in their acquired microcephaly is not shown in Figure 2 because there is no direct comparison of sex differences in the same experiment.

2. The same problem occurs in Fig 4 in which there is no mention of sex as a biological variable in the behavioral assays. The results graphed in Fig A and B are examining sex stratified results, but the % phosphorylated neurons is too variable and the n is too low for there to be an informed conclusion about the relationship between Mvp and Mapk3 alleles. Panel C is not informative because the statistics were not performed to examine sex and genotype effects, including all genotypes of the litter. Overall, the results in this figure are confusing, mostly not sex stratified and are not supportive of the conclusion in the abstract "the double hemideletion Mvp::Mapk3 rescues the brain size phenotype of Mvp deficient male mice, suggesting that MVP and ERK are involved in the same signalling pathway in vivo with MVP possibly acting as an upstream regulator for ERK signalling controlling brain size".

3. At a level of significance there is the concern that if the Mvp deficient phenotype is only at the level of brain size but not any detectable behavioral phenotypes. There is some novelty in the male-specific brain anatomical differences, but it is not clear how this contributes to the phenotype of 16p11.2 deletion phenotypes.

4. The 16p11.2del/+ mouse model was mentioned as being studied in the methods, but I could not find any results associated with this model. The inclusion of this mouse model as a comparison is important for the study's significance.

Minor concerns

1. The writing and organization of the manuscript was confusing overall. The wording should be more precise in referring to mouse genotypes with appropriate nomenclature and in describing groups being compared for specific phenotypes. There are some specific places that could be improved as well:

a. Background (p 5): the section describing the importance of sex differences in ASD and the developing brain is woefully under-referenced. There are many more publications on sex differences in the early brain than the single one referenced.

b. Background (p. 6): there are two paragraphs introducing the major vault protein, but the order is confusing. The first paragraph could be anatomical localization and expression and the second one on function. Any known sex differences or interaction with sex hormones should be discussed under Mvp function. The reference to drug delivery seems disconnected to anything else.

c. Results (p 12, line 232): the sentence is confusing as written, should likely be "we performed subsequent studies specifically comparing Mvp-/- to Mvp +/+.

d. Results (p 13, line 270-271): the conclusion sentence needs a qualifier, like "suggesting that Mvp has no major role on transcriptional regulation that could explain the phenotypes at the time point sampled."

e. Discussion: Why the authors claim that their study offers insights into mechanisms of sex differences in ASD, there is actually not discussion of sex-specific mechanisms in the manuscript. A more thorough introduction and discussion of how hormonal differences may interact with Mvp is warranted.

**Reviewer 2**

**Were you able to assess all statistics in the manuscript, including the appropriateness of statistical tests used?**

Yes, and I have assessed the statistics in my report.

**Comments to author:**

The authors used mouse models to characterize the effects of single genes withing the 16p11.2 recurrent CNV region to delineate the contribution of each gene to neuroanatomical features. This deletion is among the most frequent causes of ASD and has a high degree of variable expressivity. Carriers of the deletion may have abnormal brain size, and decreased cortical thickness occurs only in male carriers. The deletion region contains 30 protein coding genes. The authors examined neuroanatomical phenotypes on knock-out mice for 20 of these genes.

To assess neuroanatomical features the authors obtained coronal sections from specific locations (Bregma +0.98 mm and Bregma -1.34 mm) for each brain examined allowing a robust comparison of each analyzed feature.

The authors analyzed males and females separately and found significant differences, with the females being less affected across all genotypes. Out of all the genes examined, Mvp which encodes for the major vault protein showed the strongest effect. The authors then created a double knock out of Mvp and Mapk3. The double knock out rescued the neuroanatomical phenotype observed in the Mvp mutant, suggesting that Mvp is an ERK-signaling repressor.

The paper is well written, and the findings are of interest to the neurodevelopmental disorders community. The analysis of sex differences in neurodevelopmental outcomes is often overlooked and should be highlighted. In addition, the role of the vault organelle in the pathogenesis of neurodevelopmental disorders is also novel and could be relevant to several other disease risk genetic variants.

Comments:

The authors do not specify whether the mutant mice used for neuroanatomical phenotyping were heterozygous or homozygous. They do mention that for Mvp the heterozygotes had similar alterations compared to homozygous, but for the heatmaps shown on Extended figure 2 and 3 it is not clear if they are +/- or -/-. Given that the deletion in human carriers is heterozygous, the phenotypes of the heterozygous mice will be more meaningful than those of a homozygous deletion that will likely be more deleterious.

It would be informative to show the same neuroanatomical analysis on the full deletion mouse. It would be especially interesting to compare the phenotype of the 16p11.2 deletion mouse model to that of the single Mvp mutant and the double Mvp/Mapk3. If Mvp is the single most relevant gene responsible for the neuroanatomical phenotype, then the measurements for the deletion mouse should be similar to the Mvp mutant (male).

The authors examined brain size at different timepoints and found that microcephaly happens between P10 and P45 and they conclude that microcephaly is acquired rather than primary. Congenital microcephaly has been associated to deficits in neural progenitor cells (NPCs) in embryonic stages of brain development, while acquired microcephaly is less well understood but has been associated to deficits in neuronal migration and maturation and myelination deficits. The authors should explore both possibilities by performing immunohistochemical staining on the Mvp knock-out mouse at embryonic stages to look at NPCs and at early postnatal stages to identify potential defects in oligodendrocyte generation or neuronal migration defects.

The authors describe performing "transcriptomic analysis" in the cingulate gyrus of the Mvp knock-out mouse. They report no differentially expressed genes. There is no mention in the methods section of which bioinformatic analysis was done for this dataset.

In the discussion, the authors discuss the implications of there being no differentially expressed genes in the cingulate gyrus of Mvp KO vs WT. In this context they mention that the vault organelle could be implicated in the transport of mRNA to the synapses for local translation. This hypothesis could be tested by performing RNA-sequencing on the synaptosomal fraction of Mvp KO mouse brains.

**Authors Response**

**Point-by-point responses to the reviewers’ comments:**

**Reviewer #1**

This study used an impressive number of mouse models of mutations in 20 different genes within the syntenic region of human 16p11.2 and performed extensive neuroanatomical phenotyping. They identify the major vault protein as a major contributor to microcephaly in males, despite no apparent differences in behavior. They also attempt to make a connection to Mapk3 which is another gene in the locus, but the results shown are not particularly convincing. Overall, the study is potentially important for others in the 16p11.2 field, but as presented the results are confusing and do not substantiate the claims made in the abstract.
***Response****: We thank Reviewer #1 for his/her careful reading of the manuscript and thoughtful suggestions. To improve the clarity of the manuscript, we have now edited the manuscript substantially throughout the entire text including the Abstract, and as a result, we believe that the flow is significantly improved and easier to follow, and that our results fully substantiate the claims in the Abstract.*

*Detailed responses of each of the concerns raised by Reviewer #1 are provided in the following pages.*

Major concerns :

1. Neither Figure 2 nor the legend mention the sex of the mice in the figure and the Results section related to Fig 2 does not either, despite starting with "we thought to determine the origin of Mvp sex-specific differences in brain size." This appears to leave a major question in this study unanswered, namely, is there a sex difference in the level of Mvp protein or transcript in any of the brain regions where it is expressed? There is a statement in the Results claiming "no apparent sex differences were detected in the pattern of MVP/vault expression," but there is no data showing this conclusion.

***Response****: We thank Reviewer #1 for his/her comment. We apologize as we missed adding this important piece of information in a systematic manner. A color code was used instead but we can’t agree more with the Reviewer this wasn’t explicit enough so we now clearly added the sex of the mice in: (i) Figure 2, (ii) the legend of Figure 2, and (iii) the corresponding text. We did the same for all the Figures of the paper and clearly added sex information explicitly in the Figures and associated texts throughout the manuscript and the supplementary information files.*

*To comment on the expression studies, we systematically quantified in both male and female WT mice the levels of Mvp transcript and protein. This is now made clearer:*

*·	For qRT-PCR, we provide side-by-side plots of male and female Mvp expression (liver, cerebellum, hippocampus and cortex) at the top of Figure 2 (based on n=3 animals per group per sex).*

*·	For immuno-fluorescence assays, we focused on MVP co-expression with neurons based on previous literature reporting MVP expression in the cytoplasm, neurites and growth cone of neurons (Paspalas et al. 2009 Cerebral Cortex). Quantitative analysis was done using 3 male and 1 female WT mice (derived from the Mvp-mouse line; genetic background C57BL/6N) and MVP expression was scored in neurons as mild (+), moderate (++) or strong (+++) across 36 positive brain regions in both sexes throughout the entire tissue block. These 36 regions are listed in Extended Table 3 and some are illustrated in the new Figure 3. Considering that the data was similar between the female and the three male mice assessed, we report the findings in a joint summary table in Extended Table 4. Based on these quantifications, we found no detectable differences in the neuronal populations in the level of MVP expression levels in any of the brain regions where it is expressed between males and females. As an example of a positive brain region, we show IF images of male and female somatosensory cortex in the new Figure 3, side-by-side to facilitate comparison.*

*We cannot however exclude possible sex differences in the levels of Mvp expression in other cell types of the brain that will require further investigations. We made adjustments to the text to reflect this limitation of our current study. For example, we added the following paragraph to the end of the second Result section (entitled: The major vault protein is expressed in the limbic system in both sexes):*

*“To sum up, our data show no apparent sex differences in the pattern of neuronal MVP/vault expression that could explain the sex-differences in NAPs. We cannot however exclude possible sex differences in the levels of MVP expression in other cell types of the brain that will require further investigations.”*

*For future investigation, we will take advantage of the emerging studies applying cutting-edge single-cell transcriptomic technologies at unprecedented scales to better understand the mammalian brain where brain cells can be profiled in specific regions or in targeted cell types. Sex-specific changes are currently overlooked in these large-scale expression studies, the authors are almost always picking one sex to work on. Very interestingly, this new study from Jin et al. (bioRxiv) provides a brain-wide transcriptomic cell-type atlas of the entire mouse brain both in males and the females. We contacted the authors to retrieve Mvp brain cell expression data in both sexes and the data will become available at the Neuroscience Multi-omic Data Archive (NeMO) and the visualization resources at NeMO analytics (*[*https://nemoanalytics.org/*](https://nemoanalytics.org/)*) at a later stage (when their paper will be published).*

Furthermore, the potentially very important conclusion that the male Mvp-/- mice were unique in their acquired microcephaly is not shown in Figure 2 because there is no direct comparison of sex differences in the same experiment.

*R****esponse****: We added the analysis of the brain size for female mice in the Figure where male Mvp-KO mice are shown to be unique in their microcephaly phenotype (see new Figure 3 Panel E). This now allows a direct comparison of sex differences in the same experiment.*

2. The same problem occurs in Fig 4 in which there is no mention of sex as a biological variable in the behavioral assays.

***Response****: We added the sex of the mice in the behavioral assays in the new Figure 5.*

The results graphed in Fig A and B are examining sex stratified results, but the % phosphorylated neurons is too variable and the n is too low for there to be an informed conclusion about the relationship between Mvp and Mapk3 alleles. Panel C is not informative because the statistics were not performed to examine sex and genotype effects, including all genotypes of the litter.

***Response****: We thank Reviewer #1 for his/her comment and for his/her careful review, we are grateful. The generation of the double knockout Mvp;Mapk3(DKO) mice was tedious and started at IGBMC Strasbourg where we aimed at collecting brain samples for both male and female mice across the four genotypes of the litter for various experiments including (i) the measurement of phosphorylated neurons (done by experts in ERK signalling in Cardiff), (ii) the neuroanatomical phenotypes, and (iii) the behavioral assays. For (i), we aimed for at least 5 animals per group, so we agree the n is sometimes low for some of the groups (Panels A and B). For (ii), the experiments required a different preparation protocols for the brain samples, so we could not use the same samples than for (i) and had to generate additional breedings.*

*For (ii) here are the number of brain samples we collected for neuro-anatomical phenotyping: for males DKO n=6 wtwt, 1 wthet, 3 hetwt and 4 hethet; for females DKO n=0 wtwt, 3 wthet, 4 hetwt and 3 hethet. In 2019, the lab moved from Strasbourg to Dijon where we imported all of our mice aiming to complete the groups with low n. Due to lockdowns, and instructions we had received to keep our mouse colonies to the strict minimum, these experiments were finally not possible anymore as we unfortunately lost the Mapk3 line during the pandemic. Because we didn’t have any female wtwt animals, we did not have the female controls to include in Panel C. As for the males, the n was low for wthet and hetwt mice, explaining why only the wtwt and hethet groups were plotted in Panel C.*

*Although some of the results are interesting, we agree with Reviewer #1, we can’t properly conclude on the relationships between alleles, anatomy, sex, and measurements of phosphorylated neurons and will need to consolidate these initial findings. For these reasons, we moved the results as preliminary to the end of the Supplementary Information document and have made changes to the corresponding text, notably we added this sentence at the end of the Result section (right before the Discussion):*

*“Finally, preliminary findings, where we examined ERK activity in the cortex of our various mouse models in males and females, indicate that ERK activity is not associated with NAPs or behavioral phenotypes presented in this study (****Extended Fig. 12M-P*** *and* ***Supplementary Results****)”.*

Overall, the results in this figure are confusing, mostly not sex stratified and are not supportive of the conclusion in the abstract "the double hemideletion Mvp::Mapk3 rescues the brain size phenotype of Mvp deficient male mice, suggesting that MVP and ERK are involved in the same signalling pathway in vivo with MVP possibly acting as an upstream regulator for ERK signalling controlling brain size".

***Response****: We agree with Reviewer #1. As mentioned above, we agree the data presented in Panels A, B and C are preliminary and not sufficiently investigated. We moved these Panels as Supplementary Figures and presented the data as preliminary in the Supplementary Results section, leaving the behavioral studies in the main Figures as the n was good (12 animals per group) and the work extensive. We made changes in the text of the Result section related to this figure and tuned down our conclusions in the Abstract. In addition, we moved the corresponding Mvp^-/-^ and Mapk3^+/-^ behavioral data in the main figure to facilitate comparison in the behavioral studies between the various alleles.*

*We believe these changes will make the figure and the associated Result section clearer.*

*Here are the main paragraphs we added at the start of the Result section that relates to Behavior:*

*“To determine if NAPs in male Mvp^-/-^ mice lead to specific behavioral phenotypes, we assessed a broad range of sixteen behavioral tests evaluating eleven core behaviors (anxiety, depression, anhedonia, memory, locomotion, coordination, motricity, sociability, schizophrenia, autism and epilepsy). The behavioral pipeline is described in details in the* ***Supplementary Methods*** *and the raw data available in* ***Datasets 4-6****. Intriguingly, we found no major behavioral anomalies in male Mvp^-/-^ mice (****Fig. 5*** *and* ***Extended Fig. 10****). We thought to test whether introducing an additional stressor might underscore behavioral implication. To explore this idea, we generated and characterized the behavior of double male KOs of Mvp and Mapk3 (****Extended Fig. 12*** *and* ***Supplementary Methods****) since studies have consistently shown MVP-mediated regulation of ERK signalling pathway^31–33^, which is known to be one of the main pathways disrupted at the 16p11.2 locus^23^.”*

*We report sex stratified results in all figures except in this last figure, limited to males. We choose not to study behavior in females considering that we did not find any neuroanatomical phenotypes in the female Mvp^-/-^ and Mapk3^+/-^ mouse brains.*

3. At a level of significance there is the concern that if the Mvp deficient phenotype is only at the level of brain size but not any detectable behavioral phenotypes.

***Response****: We thank Reviewer #1 for his/her comment. We understand why the lack of correlation between neuroanatomical and behavioral phenotypes in the Mvp-deficient mice might cause concern.*

*One possible explanation could be due to the high sensitivity of genetic factors (such as Mvp deficiency) to environmental stressors (i.e. gene by environment interactions) such as those reported in autistic patients (Hessl et al. 2001 Pediatrics) as well as in mouse models of autism spectrum disorders (Petroni et al. 2022 Frontiers in Cellular Neuroscience; Petroni et al. 2022 Scientific Reports). Interestingly, in this latter paper, the behavioral deficits in Fmr1-KO male mice were induced by chronic stress exposure, without exacerbating the behavioral phenotype of mutant females.*

*Based on these models, it could be that we did not detect any behavioral differences, simply because we strictly limited the stress in the environment where the mice were phenotyped (for example, we gave several days of rest between experiments, we made sure that the same investigator was handling the mice etc). We thus hypothesize that environmental adversity might be interacting with the Mvp-genetic mutation potentiating behavioral concerns. This will require further investigation where Mvp-deficient mice are exposed to stress and phenotyped using the same protocols as those presented in the current study.*

*Furthermore, assessment of brain sections usually relies on qualitative evaluation by the human eye. Our quantitative and ultra-standardized approach allows us to detect many more features and more subtle neuroanatomical phenotypes such as the one we report in this study. We are very confident about the sex-dependent neuroanatomical phenotypes in our Mvp-deficient mice. However, we must say that compared to some of our previous studies, for example on Eml1-deficient mice that showed absence of the corpus callosum, severe subcortical heterotopia and double cortex (Collins et al. 2019 Journal of Anatomy), the brain anatomical changes we found in Mvp-deficient mice remain consistent but overall subtle. Therefore, another possible explanation might be that the behavioral phenotypes are subtle too which would have required a higher n to be able to detect.*

*Nevertheless, in the presence of a second perturbation (i.e. gene by gene interactions), Mvp-deficiency in conjunction with Mapk3-deficiency (Mvp;Mapk3 DKO) presented behavioral phenotypes revealing a complex genetic interaction between the two genes.*

There is some novelty in the male-specific brain anatomical differences, but it is not clear how this contributes to the phenotype of 16p11.2 deletion phenotypes.

***Response****: We thank Reviewer #1 for his/her comment. We assumed the reviewer was referring to the human 16p11.2 phenotype. If so, the male-to-female ratio in 16p11.2 deletion patients with intellectual disability is 1.6 to 1 (Polyak et al. 2015 Genome Medicine). Another human study assessing gender-by-gene dosage interaction demonstrated that some of the brain anatomical effects assessed by MRI were driven by decreased volumes in male deletion carriers (Maillard et al. 2015 Molecular Psychiatry). To clarify this point, we added the following sentences to the Background section:*

*“The human 16p11.2 locus is susceptible to a 600 Kb deletion (OMIM #611913) which is among the most frequent known etiologies of ASDs^13^ with a male-female ratio of about two^14^. This 16p11.2 deletion usually arises de novo and partly associates with abnormal brain size and other structural defects including a decreased cortical thickness restricted to male carriers^15,16^.”*

*4. The 16p11.2del/+ mouse model was mentioned as being studied in the methods, but I could not find any results associated with this model. The inclusion of this mouse model as a comparison is important for the study's significance.*

*R****esponse****: We agree with Reviewer #1 and have not completed these analyses for inclusion in the paper. In fact, we had acquired brain samples from two 16p11.2 deletion mouse models (Del/+) engineered on different genetic backgrounds, pure C57BL/6N and mixed C57BL/6N x C3B (Arbogast et al. 2016 Plos Genetics). Single-gene mouse mutants being all generated on the C57BL/6 genetic background, we focused on the 16p11.2 deletion mouse model made on the C57BL/6N genetic background.*

*We completed these neuroanatomical studies and have now added to Figure 1 four new Panels (Panels D-G). We made additions throughout the text in the main and supplementary documents. Notably we added the following paragraph to the Result section:*

*“Overall, the neuroanatomical profile associated with the male Del/+ mice indicated a decreased size of the majority of regions assessed but only the somatosensory cortex reached significance level and was reduced in size by 10% (p<0.05) (****Fig. 1D****). We asked whether alterations in specific cortical layers may contribute to this phenotype and found that it stemmed from the upper II-IV cortical layers that were thinner by 10% (p<0.0091) (****Fig. 1F****). We did not detect any significant NeuroAnatomical Phenotypes in the female Del/+ mice (****Fig. 1E****,****G****). ”*

*To our knowledge, this is the first report of specific sex differences in the layers II-IV of the somatosensory cortex between male and female Del/+ mice. While the majority of previous studies* *reporting brain anatomical changes in 16p11.2 deletion mice have not looked at sex differences (i.e. Portmann et al. 2014 Cell Reports, Pucilowska et al. 2015 Journal of Neuroscience and Pucilowska et al. 2018 Journal of Neuroscience), one study described fiber tract changes in males but not in female (Kumar et al. 2018 Translational Psychiatry).*

*We also added the following Discussion point that relates to these new findings:*

*“We found multiple single-gene drivers of a plethora of NAPs with opposite effect within the 16p11.2 interval that likely undergo a combination of additive and synergistic interactions as reported in this region^42–44^ and other loci^45^ (reviewed in ^46^). Our neuroanatomical study of the Del/+ mice confirms complex relationships between the genes as we only found one significant phenotype pertaining to the somatosensory cortex, suggesting that interactions between genes at the locus can hide NAPs driven by individual genes.”*

Minor concerns :

1. The writing and organization of the manuscript was confusing overall. The wording should be more precise in referring to mouse genotypes with appropriate nomenclature and in describing groups being compared for specific phenotypes.

***Response****: We thank Reviewer #1 for his/her constructive remark. We revised our wording to be more precise and used the appropriate nomenclature using the MGI guidelines (https://www.informatics.jax.org/mgihome/nomen/strains.shtml), for example, we replaced 16p11.2^+/Del^ mouse by B6N Del(7Sult1a1-Spn)6Yah (short named as Del/+).*

*As for the organization of the manuscript, we believe that the changes we made throughout the text have significantly improved the flow.*

There are some specific places that could be improved as well:

a. Background (p 5): the section describing the importance of sex differences in ASD and the developing brain is woefully under-referenced. There are many more publications on sex differences in the early brain than the single one referenced.

***Response****: We thank Reviewer #1 for his/her comment. We added key publications on the topic of sex differences in autism and 16p11.2 (human and mice) and edited the first (and second) paragraphs of the Background section.*

*The sentences “There are two main theories for the male predominance in ASDs. The extreme male brain theory, first proposed in 1997^5^, relates to the idea that ASDs could be extreme manifestations of the male brain and male-typical traits^6^ that may be leading to a male ascertainment bias^7^. The female protective theory highlights the female’s brain being able to carry higher levels of genetic damage than the male’s brain without getting ASDs^8–11^. Apart from a study on the impact of hormones on human neural stem cells^12^, the underlying sex-specific biology remains largely unknown. ” have been added to the first paragraph.*
b. Background (p. 6): there are two paragraphs introducing the major vault protein, but the order is confusing. The first paragraph could be anatomical localization and expression and the second one on function. Any known sex differences or interaction with sex hormones should be discussed under Mvp function. The reference to drug delivery seems disconnected to anything else.

***Response****: We thank Reviewer #1 for his/her thoughtful suggestion on the paragraphs that introduce the major vault protein. We re-organized the content of the two paragraphs introducing the major vault protein. As suggested by the reviewer, the first paragraph introduced the anatomy of MVP/vault, localization and expression:*

*“Described in 1986 by Leonard H Rome, the vault is the biggest ribonucleoprotein organelle present in many eukaryotic cells^24^, three times the size of a ribosome. MVP is the main constituent of the vault organelle and is sufficient to give the vault its hollow barrel-like shape^25^. Tanaka et al. have resolved the structure of the rat vault at 3.5 Å resolution as a particle made of 78 identical MVP^26^. MVP is not visible as free monomers at any given time in cells but instead appears only as vault particles^27^. MVP/vault is expressed in the cytoplasm, neurites and growth cone of rat neurons^28^. ”*

*The second paragraph introduced the known function of MVP/vault:*

*“Despite its discovery more than 30 years ago, the function of the vault organelle remains largely elusive. In vivo, MVP/vault associated with cortical plasticity in a monocular deprivation mouse model^29^. In vitro, the vault organelle binds to microtubules^30^, MVP interacts with ERK through epidermal growth factor signalling^31,32^ and associates with Aurora-A to enhance synapse formation and activity by the regulation of ERK signalling^33^. Interestingly, MVP has been coimmunoprecipitated and colocalized with steroid hormone receptors in human cancer cells^34,35^ and is involved in cellular trafficking for the modulation of Src-dependent ERK signal transduction induced by sex-steroid hormones^31^. ”*

*There is hardly no literature describing a link between MVP/vault and sex hormones. We only found two papers and added them in the Background section:*

*·	Abbondanza and colleagues reported an interaction between vault particles and estrogen receptor in the MCF-7 breast cancer cell back in 1998.*

*·	More recently, Nunes-Xavier and co-workers found a correlation between expression of the major vault protein and the androgen receptor in prostate cancer.*

*We removed the reference to drug delivery. We agree with Reviewer #1 as not being connected to the present study.*

c. Results (p 12, line 232): the sentence is confusing as written, should likely be "we performed subsequent studies specifically comparing Mvp-/- to Mvp +/+.

***Response****: We thank Reviewer #1 for his/her comment. We corrected the sentence as suggested by the reviewer.*

d. Results (p 13, line 270-271): the conclusion sentence needs a qualifier, like "suggesting that Mvp has no major role on transcriptional regulation that could explain the phenotypes at the time point sampled."

***Response****: We thank Reviewer #1 for his/her comment. We added the qualifier as suggested by the reviewer.*

e. Discussion: Why the authors claim that their study offers insights into mechanisms of sex differences in ASD, there is actually not discussion of sex-specific mechanisms in the manuscript. A more thorough introduction and discussion of how hormonal differences may interact with Mvp is warranted.
*R****esponse****: We thank Reviewer #1 for his/her comment. We changed the wording and tuned down our claim. There are only two papers describing a link between MVP/vault and sex hormones. We added them in the Background section. In fact, the overall topic of sex-specific biological mechanisms is largely understudied. In future studies, we will need to gain more knowledge on the molecular and cellular mechanisms of hormonal differences and how they interact with MVP/vault biology to modulate brain anatomy.*

**Reviewer #2**

The authors used mouse models to characterize the effects of single genes withing the 16p11.2 recurrent CNV region to delineate the contribution of each gene to neuroanatomical features. This deletion is among the most frequent causes of ASD and has a high degree of variable expressivity. Carriers of the deletion may have abnormal brain size, and decreased cortical thickness occurs only in male carriers. The deletion region contains 30 protein coding genes. The authors examined neuroanatomical phenotypes on knock-out mice for 20 of these genes.

 To assess neuroanatomical features the authors obtained coronal sections from specific locations (Bregma +0.98 mm and Bregma -1.34 mm) for each brain examined allowing a robust comparison of each analyzed feature.

The authors analyzed males and females separately and found significant differences, with the females being less affected across all genotypes. Out of all the genes examined, Mvp which encodes for the major vault protein showed the strongest effect. The authors then created a double knock out of Mvp and Mapk3. The double knock out rescued the neuroanatomical phenotype observed in the Mvp mutant, suggesting that Mvp is an ERK-signaling repressor.

The paper is well written, and the findings are of interest to the neurodevelopmental disorders community. The analysis of sex differences in neurodevelopmental outcomes is often overlooked and should be highlighted. In addition, the role of the vault organelle in the pathogenesis of neurodevelopmental disorders is also novel and could be relevant to several other disease risk genetic variants.

*R****esponse****: We thank Reviewer #2 for his/her high-quality and constructive reviews of our manuscript for improvements, and careful reading of our manuscript and thoughtful suggestions.*

Comments:

The authors do not specify whether the mutant mice used for neuroanatomical phenotyping were heterozygous or homozygous. They do mention that for Mvp the heterozygotes had similar alterations compared to homozygous, but for the heatmaps shown on Extended figure 2 and 3 it is not clear if they are +/- or -/-. Given that the deletion in human carriers is heterozygous, the phenotypes of the heterozygous mice will be more meaningful than those of a homozygous deletion that will likely be more deleterious.

***Response****: We thank Reviewer #2 for his/her comment. We agree with the reviewer that the phenotypes of the heterozygous mice are more meaningful than those of the homozygous in the context of the deletion in human carriers that occurs de novo. In fact, the heatmaps are reporting the heterozygous (HET) mice at the exception of a few cases when we could only acquire homozygous mice. In Extended Figures 2 and 3, the allele type (HET) is indicated in the allele names along the vertical axis at the end of the name. This was added to the legend of both Extended Figures so it becomes explicit.*

*To facilitate comparisons, we also added a new Panel at the top of the new Figure 4 to show the phenotypes of the heterozygous Mvp mice above the existing Panel showing the homozygous Mvp mice. In Panel A (Mvp^+/-^ mice), there are a total of 14 brain regions showing significant brain anatomical changes when compared to WT mice as seen by the number of colored bar plots. In panel B (Mvp^-/-^ mice), 17 brain regions are altered when compared to WT mice. We made the following changes to the text:*

*“At P120, het male Mvp^+/-^ mice showed the same reduction in total brain size (-9%, p=0.016) when compared to hom male Mvp^-/-^ mice (-9%, p=0.046) (****Fig. 4A-B****). Consistently, both Mvp^+/-^ and Mvp^-/-^ male mice showed small brain nuclei associated to the limbic system such as the cingulate gyrus (Mvp^+/-^: -13%, p=0.0016; Mvp^-/-^: -8%, p=0.007), the somatosensory cortex (Mvp^+/-^: -12%, p=0.0025; Mvp^-/-^: -12%, p=0.0016) and the hippocampus (Mvp^+/-^: -20%, p=0.023; Mvp^-/-^: -24%, p=0.038)”.*

*In the context of Mvp function, we performed studies specifically comparing Mvp homozygous KO mice to WT mice (Figure 4C-G and Figure 5), to respect the 3R guidelines (Replacement, Reduction, and Refinement).*

It would be informative to show the same neuroanatomical analysis on the full deletion mouse. It would be especially interesting to compare the phenotype of the 16p11.2 deletion mouse model to that of the single Mvp mutant and the double Mvp/Mapk3. If Mvp is the single most relevant gene responsible for the neuroanatomical phenotype, then the measurements for the deletion mouse should be similar to the Mvp mutant (male).

***Response****: We thank Reviewer #2 for his/her comment. Adding the full deletion mouse to the paper was also requested by Reviewer #1. We agree with both reviewers and have now added this complete piece of work (see our reply to Point 4 of Reviewer #1 above).*

*The assumption made by Reviewer #2 is correct. If Mvp is the main driver for the neuroanatomical phenotype, then the measurements for the 16p11.2 deletion mouse should be similar to the Mvp mutant (male). However, overall, this is not what we found. Instead, we identified multiple main drivers for neuroanatomical phenotypes (see Figure 1H-J) in which the phenotypes are the result of multiple gene effects* *with complex relationships, referred to as the “complex cis-epistatic model” in the review paper from Dr. Christelle Golzio and colleagues (Golzio et al. 2013 Current Opinion in Genetics and Development). These complexities in the underlying genetic architecture have also been reported in a previous study for craniofacial measurements (Qiu et al. 2019 Cell Reports) including synergistic effects between MVP, MAPK3 and KCTD13 (three genes in the locus).*

*Based on our findings, we can say that: (i) Mvp is the top driver (but not a single driver) for the neuroanatomical phenotypes at the autism-associated 16p11.2 locus, and (ii) Mvp is implicated in brain neuroanatomy in a sex-dependent manner with Mvp-inactivation predominantly affecting the male sex. We made small changes to the title of the paper to reflect on this comment.*

*Our new title is “Dissecting the autism-associated16p11.2 locus identifies* ***multiple*** *drivers in neuroanatomical phenotypes and unveils a* ***male-specific*** *role for the major vault protein.”*

The authors examined brain size at different timepoints and found that microcephaly happens between P10 and P45 and they conclude that microcephaly is acquired rather than primary. Congenital microcephaly has been associated to deficits in neural progenitor cells (NPCs) in embryonic stages of brain development, while acquired microcephaly is less well understood but has been associated to deficits in neuronal migration and maturation and myelination deficits. The authors should explore both possibilities by performing immunohistochemical staining on the Mvp knock-out mouse at embryonic stages to look at NPCs and at early postnatal stages to identify potential defects in oligodendrocyte generation or neuronal migration defects.

***Response****: We thank Reviewer #1 for his/her thoughtful suggestion. The distinction between congenital (primary) and acquired (secondary) is a very interesting topic. In our case, the brain size phenotype is detected postnatally upon Mvp-deficiency between P10 and P45 (male). The cellular deficits that cause this postnatal phenotype is unlikely to stem from deficits in embryonic neurodevelopment. We are highlighting the reasons below:*

*·	First, based on an existing resource of expression profiles across multiple organs and developmental stages (Cardoso-Moreira et al. 2019 Nature), Mvp is not expressed before birth in the human and before E16.5 in the mouse brain (see Figures below). This is corroborated by this single-cell transcriptomic altas of the developing neocortex that did not detect any Mvp expression at E14.5 (Telley et al. 2016 Science). The peak of expression is postnatal between P28 and P63 in the mouse. It is therefore unlikely that the deficits lie in neural progenitor cells which peak at around E14.5.*

#### *Human - ENSG00000013364 (Adapted from Cardoso-Moreira et al. 2019 Nature)*

#### *Mouse - ENSMUSG00000030681(Adapted from Cardoso-Moreira et al. 2019 Nature)*

*·	Second, white matter structures (such as the fimbria, the anterior commissure, the internal capsule) rich in oligodendrocytes were not affected in Mvp-deficient mice instead the regions affected pertain to the limbic system (such as cingulate cortex, hippocampus, piriform cortex) where the number of oligodendrocytes is much smaller. Furthermore, the amount of myelin, which we measured by Luxol staining, was not altered in our P45 and P120 cohorts, therefore defects in oligodendrocytes generation is unlikely.*

*·	Third, heterotopia (cells being misplaced) was systematically examined by two independent investigators. We verified our internal database where this information is recorded, and there was no indication of heterotopia. We can therefore exclude severe neuronal migration disorders like we have seen previously in other genes from previous studies utilizing the same standard operating procedures that the ones used in the current study. For example, in our Eml1-deficient mouse study, we found a massive heterotopia surrounded by fiber tracts and cell dispersion in the CA3 layer of the hippocampus (Collins et al. 2019 Journal of Anatomy). Another example is our work on Kif2a-knockin mouse work where we identified hippocampal heterotopia in the CA1 region with an additional layer of cells present in the stratum oriens (Gilet et al. 2020 Human Molecular Genetics).*

*·	Finally, the studies we conducted at the different time points (E18.5, P10, P45, P120) were not limited to the measurement of the size of the brain but to 41 additional measurements (listed in Extended Table 5 and illustrated in Supplementary Information Figure 3). None were affected at E18.5. Furthermore, cellular features were quantified at the various timepoints using automated plugins we developed in Fiji to measure the number of cells as well as the averaged cell area of each cell. The phenotypes at the cellular level were all consistent and showed no phenotype at the level of cell counts meaning that the deficits are not proliferation-related but a decrease size of the cell soma (detectable from P45 onwards), pointing towards deficits in the regulation of the maintenance of cell homeostasis.*

*For all of these reasons, we don’t think that studies at embryonic stages were necessary for this current study.*

The authors describe performing "transcriptomic analysis" in the cingulate gyrus of the Mvp knock-out mouse. They report no differentially expressed genes. There is no mention in the methods section of which bioinformatic analysis was done for this dataset.

*R****esponse****: We thank Reviewer #2 for his/her comment. We added a paragraph in the Methods section explaining how we carried out the transcriptomic analysis and added the following paragraph:*

*“RNA sequencing libraries were generated from 200 ng of total RNA from mouse cingulate gyrus using TruSeq Stranded mRNA LT Sample Preparation Kit (Illumina, San Diego, CA), according to manufacturer's instructions. The details are provided in the* ***Supplementary Methods****. The libraries were sequenced on the Illumina Hiseq 4000 as Single-end 50 base reads following Illumina’s instructions. RNA sequencing analysis and base calling were performed using RTA 2.7.3 and bcl2fastq 2.17.1.14. Reads were preprocessed using cutadapt 1.10^64^ in order to remove adapter, polyA and low-quality sequences (Phred quality score below 20), reads shorter than 40 bases were discarded for further analysis. Reads mapping to rRNA and spike-in sequences were also discarded (this mapping was performed using bowtie 2.2.8^65^). Reads were then mapped onto the mm10 assembly of Mus musculus genome using STAR^66^ version 2.5.3a (--twopassMode Basic). Gene expression was quantified using htseq-count 0.6.1p1^67^ and gene annotations from Ensembl release 91. Statistical analysis was performed using R 3.3.2 and DESeq2 1.16.1 Bioconductor library^68^.”*

In the discussion, the authors discuss the implications of there being no differentially expressed genes in the cingulate gyrus of Mvp KO vs WT. In this context they mention that the vault organelle could be implicated in the transport of mRNA to the synapses for local translation. This hypothesis could be tested by performing RNA-sequencing on the synaptosomal fraction of Mvp KO mouse brains.

***Response****: We acknowledge the reviewer’s suggestion to explore RNA-sequencing on the synaptosomal fraction of Mvp-deficient mouse brains. We have a close collaboration with Pr Toshiyuki Araki and his team in Japan who identified that small noncoding vault RNA modulates synapse formation by amplifying ERK signaling (Wakatsuki et al. 2021 The Journal of Cell Biology). Our grant is under review and will allow to investigate local translation in the synaptosome fractions of our mice. This will be our follow-up study to the current work.*

**Second round of review**

**Reviewer 1**

The authors have appropriately responded to past critiques and the manuscript is much improved.

**Reviewer 2**

The authors thoroughly addressed all my comments and concerns. The revised manuscript is significantly clearer that the original manuscript. I appreciate the edit made to the title and re-structuring of the abstract which make the contributions of this work clearer. The added experiments on the full deletion mouse are a welcome addition to this work and improve the delineation of MVP's role on neuroanatomical phenotypes of the deletion.

I have no further comments or concerns.
